# Reactome from a WikiPathways Perspective

**DOI:** 10.1371/journal.pcbi.1004941

**Published:** 2016-05-20

**Authors:** Anwesha Bohler, Guanming Wu, Martina Kutmon, Leontius Adhika Pradhana, Susan L. Coort, Kristina Hanspers, Robin Haw, Alexander R. Pico, Chris T. Evelo

**Affiliations:** 1 Department of Bioinformatics—BiGCaT, Maastricht University, Maastricht, The Netherlands; 2 Ontario Institute for Cancer Research, MaRS Centre, Toronto, Ontario, Canada; 3 Maastricht Centre for Systems Biology (MaCSBio), Maastricht University, Maastricht, The Netherlands; 4 Department of Pharmacy, Faculty of Science, National University of Singapore, Singapore, Republic of Singapore; 5 Gladstone Institutes, San Francisco, California, United States of America; The Krasnow Institute for Advanced Studies, UNITED STATES

## Abstract

Reactome and WikiPathways are two of the most popular freely available databases for biological pathways. Reactome pathways are centrally curated with periodic input from selected domain experts. WikiPathways is a community-based platform where pathways are created and continually curated by any interested party. The nascent collaboration between WikiPathways and Reactome illustrates the mutual benefits of combining these two approaches. We created a format converter that converts Reactome pathways to the GPML format used in WikiPathways. In addition, we developed the ComplexViz plugin for PathVisio which simplifies looking up complex components. The plugin can also score the complexes on a pathway based on a user defined criterion. This score can then be visualized on the complex nodes using the visualization options provided by the plugin. Using the merged collection of curated and converted Reactome pathways, we demonstrate improved pathway coverage of relevant biological processes for the analysis of a previously described polycystic ovary syndrome gene expression dataset. Additionally, this conversion allows researchers to visualize their data on Reactome pathways using PathVisio’s advanced data visualization functionalities. WikiPathways benefits from the dedicated focus and attention provided to the content converted from Reactome and the wealth of semantic information about interactions. Reactome in turn benefits from the continuous community curation available on WikiPathways. The research community at large benefits from the availability of a larger set of pathways for analysis in PathVisio and Cytoscape. The pathway statistics results obtained from PathVisio are significantly better when using a larger set of candidate pathways for analysis. The conversion serves as a general model for integration of multiple pathway resources developed using different approaches.

## Author Summary

Biological pathways are descriptive diagrams that describe biological processes, i.e. interactions between genes, proteins, and metabolites. Pathways can therefore be used to integrate and visualize molecular measurements of genes, proteins, and metabolites in different biological conditions, e.g. healthy state *vs*. diseased state. This helps researchers investigate a disease. For instance, the low expression of a certain gene might in turn lead to the low abundance of a certain protein which might prevent the breakdown of a certain metabolite, the accumulation of which contributes to disease progression. High throughput “omics” technologies produce vast quantities of biological measurement data. Biological pathways provide an intuitive knowledge-based scaffold for integrating these data WikiPathways and Reactome are two commonly used pathway databases. Reactome pathways are centrally curated with periodic input by domain experts, while WikiPathways is a community-based platform where pathways are created and continually curated by any interested party. As part of an ongoing collaboration between Reactome and WikiPathways, we have added the Reactome pathways to WikiPathways and made them available from the Reactome portal on WikiPathways. Here, we demonstrate how such an integration is advantageous to both the Reactome and WikiPathways communities and to the general research community at large.

## Introduction

Pathway diagrams are a common way to represent a wealth of information about biological molecules, interactions and processes. Currently, the Pathguide collection lists 45 freely available pathway databases with human data, out of which only 14 provide the data in a machine readable format [1, 2]. Even fewer of these provide a pathway diagram that can be used for data visualization and downloaded for further analysis and conversion into other formats (S1 Table). Notable among them are WikiPathways and Reactome, each with its unique user base, contributors, and curation cycle [3].

WikiPathways is an open, collaborative platform for drawing, curating, and sharing biological pathways, built using the same MediaWiki software underlying Wikipedia. WikiPathways leverages community curation to grow and maintain its pathway collection beyond the capabilities of an internal curation team. Anybody can register at WikiPathways to create new pathways and curate existing ones. WikiPathways provides a JavaScript-based viewer for interactively navigating and highlighting pathway elements and a Java based editor for creating and curating pathways. It makes use of BridgeDb web services [4] to provide identifier resolution and links to primary data sources. Pathways can be tagged for classification and quality control, e.g. pathways with the tag “curated” are regularly checked by a dedicated curation team and are deemed suitably annotated for analysis [5]. Pathways can also be tagged with various ontology tags from various pre-existing established ontologies, such as the Pathway ontology [6] and Disease Ontology [7]. Pathways from WikiPathways can be used to integrate, visualize, and analyze system-wide transcriptomics, proteomics, and metabolomics measurements using the open source pathway analysis tool PathVisio [8]. Pathways can also be analyzed as networks in Cytoscape [9], using the WikiPathways app to convert the pathways into networks [10]. WikiPathways pathways are also used by several other tools, such as GO-Elite [11] and SNPLogic [12]. Domain experts often curate specific subsets of pathways in WikiPathways, which are made available in portals e.g. the plant portal [13, 14], CIRM portal [15], exRNA portal [16]. In addition, WikiPathways data is available in RDF (Resource Description Framework) format, which is incorporated into the Open PHACTS Discovery platform, which integrates pharmacological data from a variety of information resources and provides tools and services to question this integrated data to support pharmacological research [17, 18].

Like WikiPathways, Reactome is an open-source, open access pathway database with a substantial collection of diverse pathway models [19, 20]. However, it differs from WikiPathways as the pathway annotations are annotations are curated by the Reactome editorial staff in collaboration with external experts in the research community. Reactome provides an intuitive website to navigate pathway knowledge and a suite of data analysis tools to support the pathway-based analysis of complex experimental and computational data sets. Similar to WikiPathways, visualization of Reactome pathways is facilitated by the Pathway Browser that supports zooming, scrolling, and highlighting, and can show detailed information about entities in the pathway. It makes use of PSICQUIC web services [21] to overlay molecular interaction data from the Reactome Functional Interaction Network [22] and external interaction databases, including IntAct [23], and ChEBI [24]. Pathways in Reactome are explicitly constructed in terms of biochemical reactions and drawn in accordance with the community standard Systems Biology Graphical Notation (SBGN) [25]. Reactome also provides pathway analysis tools which can be used to perform ID mapping, pathway assignment, and over-representation or enrichment analysis with user-supplied datasets.

The integration of Reactome content in WikiPathways provides Reactome with the power of community curation and broader format availability, including the semantic format using the WikiPathways RDF generator [26]. At the same time, WikiPathways benefits from the additional content and curation attention from the Reactome team. A connection between Reactome and WikiPathways was first proposed in 2008, using either the EBI created CSV format and a novel converter, or the BioPAX format and Cytoscape [27]. However, neither of these routes was very successful in preventing loss of data. Therefore, these generic methods of conversion were abandoned for a more specific format conversion. Pathways in WikiPathways are stored using the Graphical Pathway Markup Language (GPML) format, while pathways in Reactome are stored in a relational database organized by the Reactome data model with their diagrams stored in the database as XML strings with other related information [28, 29]. We created a converter to convert pathways directly from the relational database into the GPML format.

In this manuscript, we describe the newly developed format converter to convert Reactome content for inclusion in WikiPathways. The addition of the Reactome pathways to the analysis collection of pathways available from WikiPathways improves the coverage of gene ontology biological process terms of the analysis collection to 90%. The converted Reactome pathways can be analyzed with several new analysis tools, such as the pathway analysis tool PathVisio and network analysis tool Cytoscape. As a pedagogic example, we perform pathway analysis using a publicly available transcriptomics dataset and the combined collection of pathways from WikiPathways and Reactome.

## Results

We developed a Java based format converter to convert Reactome pathways into the WikiPathways format (see Methods). The converter was used to convert the human pathways from Reactome. 431 pathways were converted from version 54 of Reactome, tagged as “reactome_approved”, and made available from the Reactome portal [30]. The same converter was also used to convert pathways from Plant Reactome. 102 pathways were converted and added to the Plant portal in WikiPathways [14].

### Pathway view: WikiPathways vs. Reactome

A pathway in WikiPathways consists of data nodes, interactions, and graphical elements, e.g. cellular compartments. Data nodes can be of the following types: *gene product*, *protein*, *RNA*, *metabolite*, *pathway*, *complex*, and *unknown*. *Gene product* is the default data node that can be used for all products of genes such as transcripts, proteins, RNAs, and genes. By default, these are represented as open rectangular boxes with black labels and borders. The more specific data node types such as *protein* and *RNA* can be used in the specific cases instead of a generic *gene product* node. The *protein* node is visually the same as the *gene product* node while RNAs are represented in purple. The *metabolite* node represents metabolites, drugs, or other small molecules; it is represented in blue. The *pathway* data node is used to denote a connection to another pathway, and represented in green without a border. The *complex* data node represents two types of complexes either a set of proteins represented as a brown rounded rectangle or a set of interacting proteins represented by a brown hexagon. Data nodes of type *unknown* are represented the same as the generic *gene product* node. Interactions describe the relationship between two data nodes. Currently, two collections of interactions are available in the drawing palette: basic interactions and Molecular Interaction Map (MIM) interactions [31]. Arrows can be used to describe basic interactions like conversion, translocation, activation, binding, and modification. T-bars denote inhibition. The MIM interaction palette can be used for more formal and easier machine-readable descriptions of Binding, Conversion, Catalysis, Stimulation and Necessary Stimulation, and Transcription/Translation. Graphical elements can be used to provide contextual meaning to the pathways. Graphical Shapes, lines, and labels can for instance be used to annotate a biological process and generally to make things visually clearer to biologists. Similarly, graphical cellular compartments such as mitochondria, endoplasmic reticulum, nucleus and cell walls can be also added to the pathway as predefined shapes for a richer diagram.

Reactome uses a comparable but graphically slightly different method to describe pathway content. In Reactome, the core unit of the data model is the reaction. Entities (nucleic acids, proteins, complexes, and small molecules) participate in reactions. These reactions form a network of biological interactions and are grouped into pathways. Reactome uses the SBGN Process Description format [17] to draw pathway diagrams. Small Molecules are represented by a green oval, proteins by a green rounded rectangle, and complexes by blue hexagons. A group of entities playing the same roles in a reaction is annotated as EntitySet in Reactome, which is displayed as rounded rectangle with a double line border. Organelles are represented by orange rectangles with double line borders for membranes or single line borders for non-membrane organelles. The following reaction types can be represented: Transition/Process, Association/Binding, Catalysis, Inhibition, Dissociation, Omitted, and Uncertain. Stoichiometry, catalysis, positive and negative regulation, and other types of reaction attributes can also be represented in pathway diagrams based on SGBN.

**Fig 1:**
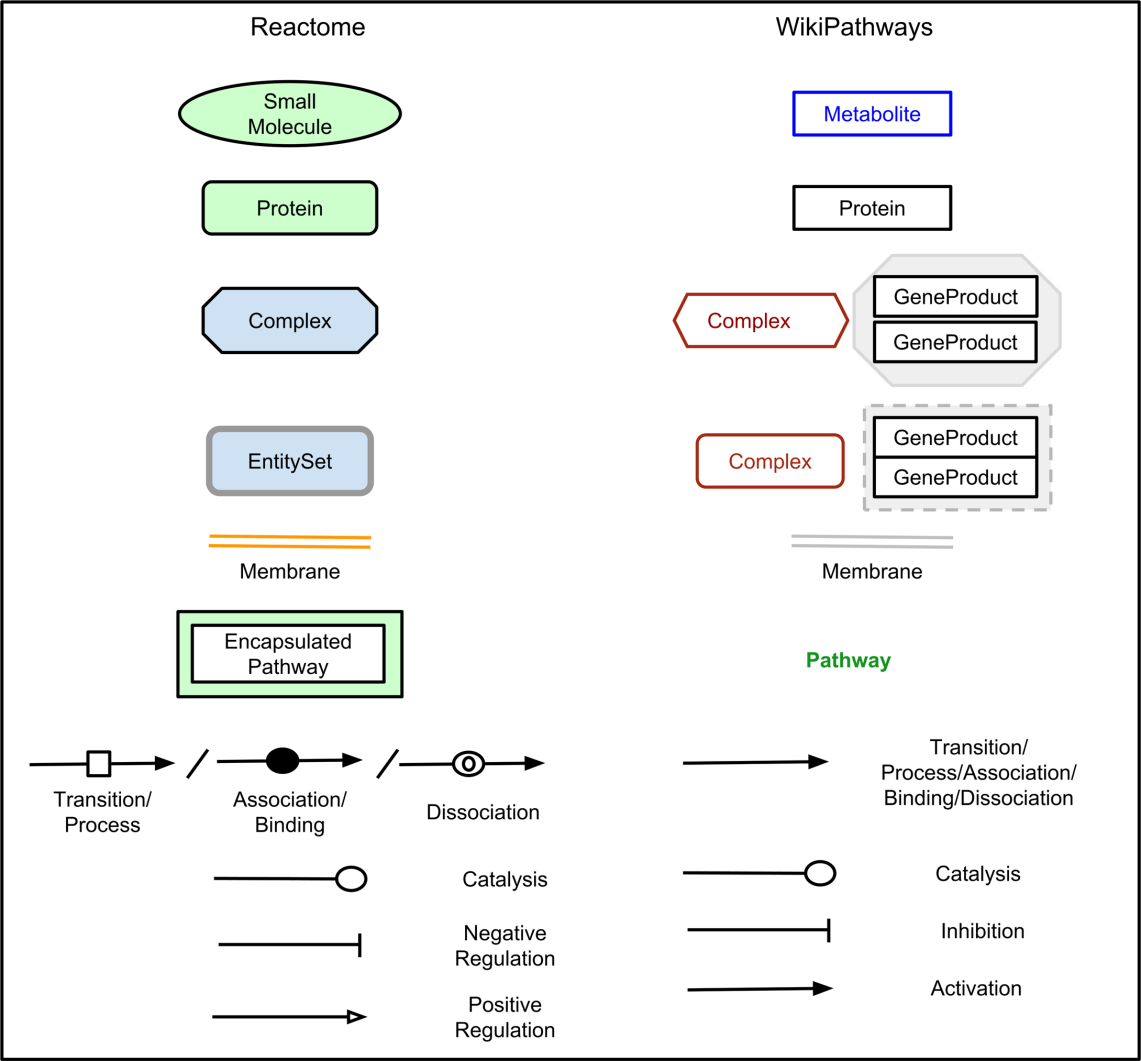
Mapping Reactome pathways elements to WikiPathways pathway elements. This diagram shows the symbols used to represent different biological entities in Reactome and the corresponding symbol used to represent the same biological entity in WikiPathways.

Fig 1 shows how the pathway elements from the Reactome pathways were represented in the converted pathway.

**Fig 2:**
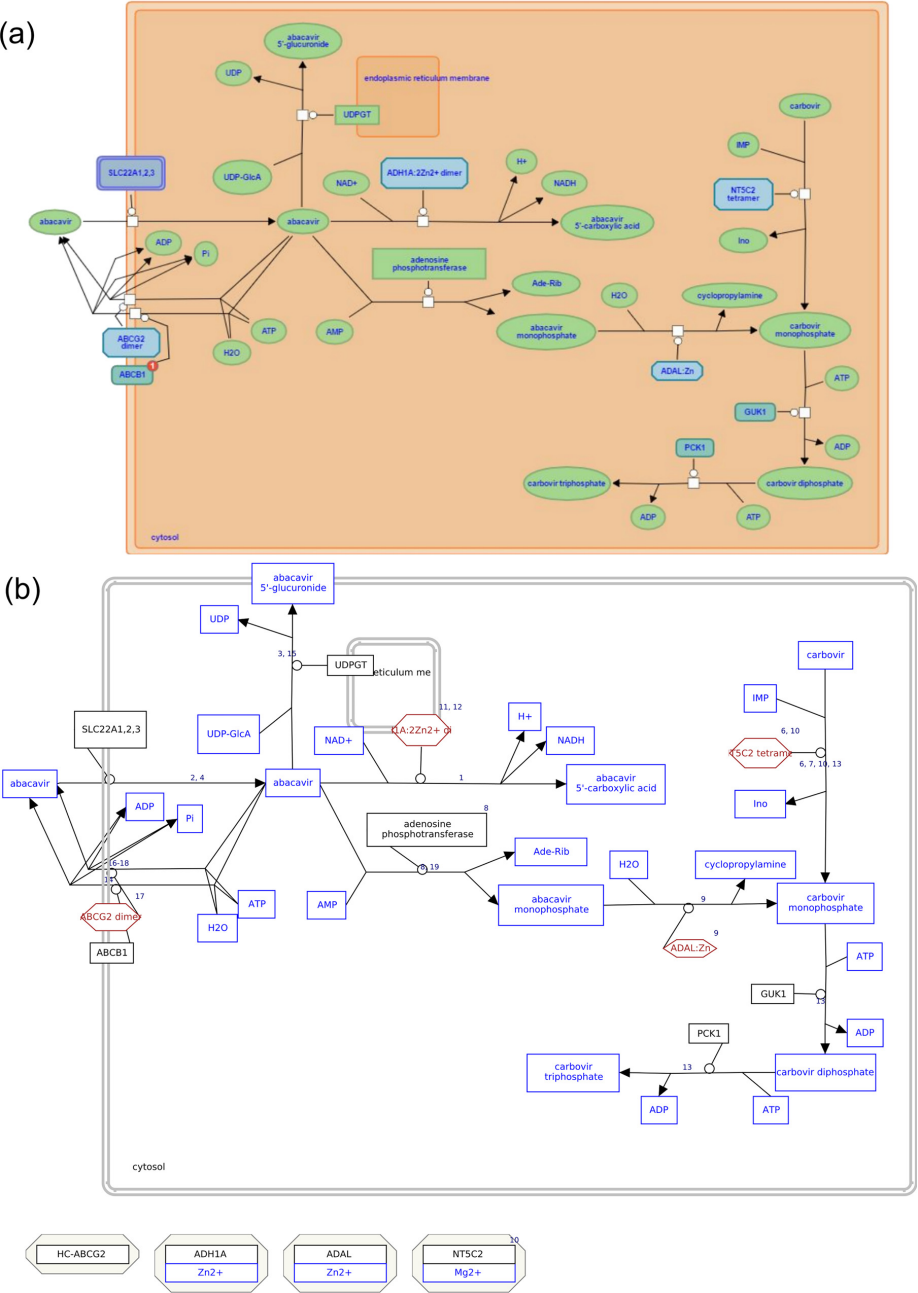
A comparative view of the Abacavir transport and metabolism pathway for Homo sapiens from Reactome Database (version 54). (a) Reactome View of Abacavir transport and metabolism (Homo sapiens) [32] and (b) Pathway view on WikiPathways(WP2712_r83598) [33].

Each element of the pathway can be annotated using database identifiers for data analysis and also annotated with literature references. As an example of the conversion, the Abacavir transport and metabolism pathway is shown here (Fig 2), example of a larger pathway is provided (S1 Fig). In addition to converting the elements of the Reactome pathway diagram, the converter also draws the components of the complexes and entity sets at the bottom of the pathway. This helps with data visualization and gives better results for pathway analysis. Because all the complex and entity set members are also present in the same pathway diagram, they are also taken into consideration by the pathway statistics algorithm for determining the importance of the pathway for the given dataset in the given condition.

The ComplexViz plugin enables the user to highlight the components on the bottom of the pathway belonging to the complex selected in the pathway diagram or vice versa.

### Reactome content improves human biological entity coverage in WikiPathways

#### Coverage of Gene Ontology terms

Gene Ontology (GO) terms provide structured vocabularies for annotating the molecular function, biological process, and cellular location of gene products in a highly systematic way [34]. Here, we analyze the coverage of all GO terms together and the biological process, molecular function, and cellular compartment GO classes separately by the genes and proteins of the curated and reactome_approved collection pathways from WikiPathways.

**Fig 3:**
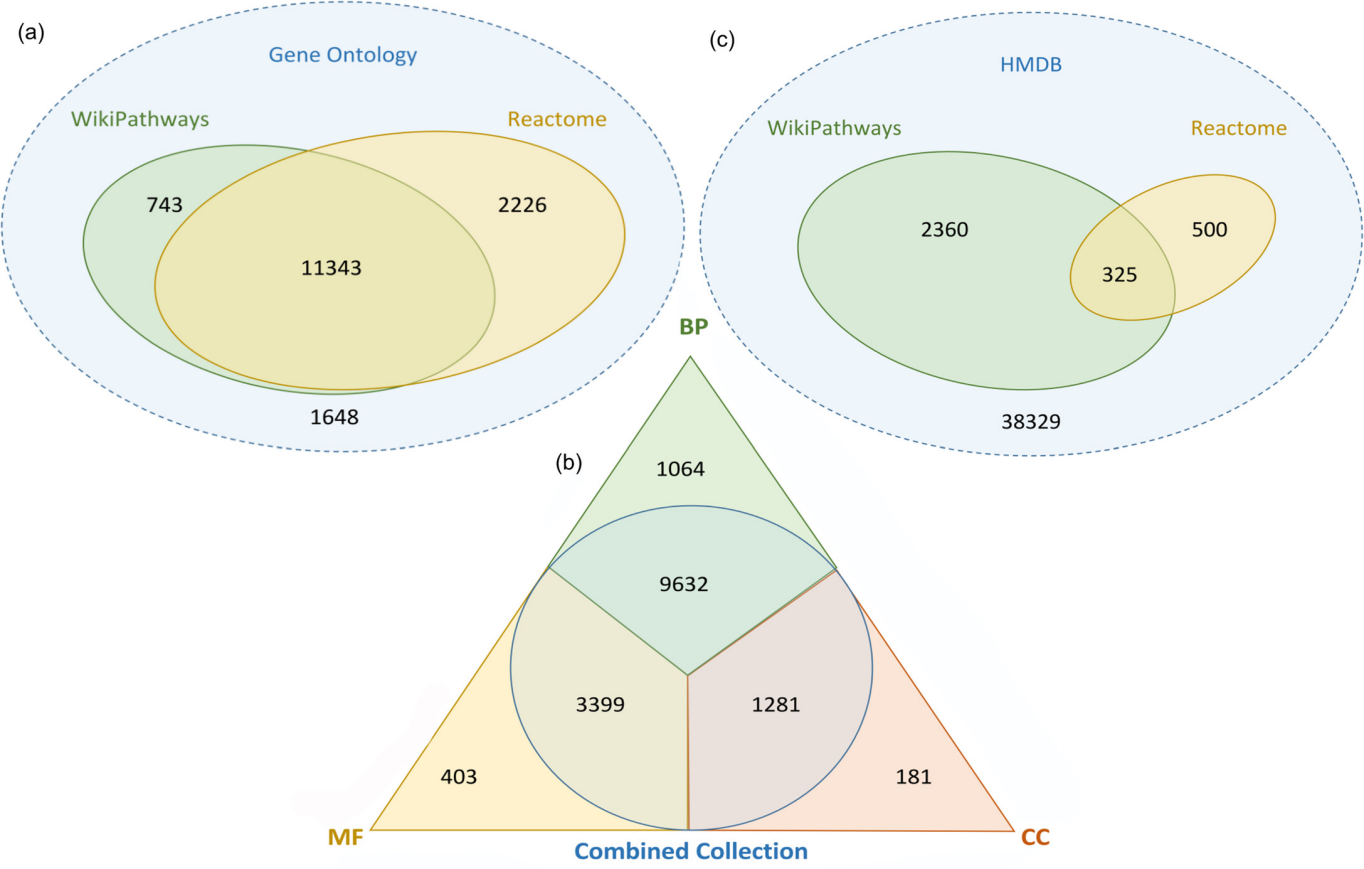
Venn diagrams showing coverage of other external databases by WikiPathways and Reactome. (a) Venn Diagram showing coverage of Gene Ontology Terms by Gene Products of WikiPathways and Reactome, (b) Venn Diagram showing coverage of Biological Process (BP), Molecular Function (MF), and Cellular Compartment (CC) Gene Ontology Terms by Gene Products of curated and reactome_approved collections of WikiPathways pathways, and (c) Venn Diagram showing coverage of the Human Metabolome Database (HMDB) by metabolites curated and reactome_approved collections of WikiPathways pathways.

Out of the 15960 human GO terms 90% are now covered by the combined curated and reactome_approved collection pathways available for analysis from WikiPathways (*i*.*e*. at least one gene in one of the pathways is annotated with the term). 11343 (71%) GO terms are covered by both collections. The curated collection of WikiPathways includes an additional 5% and the reactome_approved collection an additional 14% to bring the total coverage of GO terms up to 90% (Fig 3A). The coverage of gene ontotogy terms by the two pathway collections is shown in Table 1. The coverage of each gene ontology branch, biological process, molecular function, and cellular compartment, by the combined set of pathways from the curated and Reactome approved collections is shown in Fig 3B Venn Diagrams showing coverage of each gene ontology branch by curated collection, the reactome approved collection, their overlap, and terms not yet covered is provided (S2 Fig).

**Table 1:** Summary of the Gene Ontology Term Coverage.

Category	Total terms	Coverage by the different collections			
		Only WikiPathways	Only Reactome	Combined	Overlap
All GO terms	15960	743 (5%)	2226 (14%)	14312 (90%)	11343 (71%)
BP terms	10696	555 (5%)	1191 (11%)	9632 (90%)	7886 (74%)
MF terms	3802	147 (4%)	825 (22%)	3399 (89%)	2427 (64%)
CC terms	1462	41 (3%)	210 (14%)	1281 (88%)	1030 (70%)

GO, Gene Ontology; BP, Biological Process; MF, Molecular Function; CC, Cellular Compartment

#### Metabolome coverage

The Human Metabolome Database (HMDB) is currently the most complete and comprehensive curated collection of human metabolites [35]. Here, we analyze the coverage of HMDB by the curated and reactome_approved collection pathways from WikiPathways. (Fig 3C). 41515 unique metabolites have been reported in the current version (3.6) of HMDB. 2685 of these metabolites are covered by WikiPathways, of which 325 metabolites are covered by Reactome as well. The inclusion of Reactome pathways contributed 500 new metabolites to the WikiPathways collection.

### Plant pathways converted from Plant Reactome

The Reactome converter was also used to convert plant pathways from the Plant Reactome database freely available at http://plantreactome.gramene.org/. Pathways for the species *Oryza sativa*, *Zea mays*, and *Arabidopsis thaliana* were converted. The pathways for rice are manually curated, the pathways for the other species are computationally inferred from the rice pathways. These pathways have been made available in the plant portal at WikiPathways [14].

### WikiPathways infrastructure to analyze Reactome data

#### Pathway analysis and visualization

The conversion of pathways from Reactome has contributed 431 new manually curated pathways to the WikiPathways analysis set. In addition to the 293 pathways originally available in the curated collection. This combined set of 724 pathways are now available for analysis from WikiPathways, essentially doubling the pathway quantity. To evaluate the effect of this content enrichment on pathway analysis of genomics datasets we performed pathway analysis with the combined set.

Polycystic ovary syndrome (PCOS) is a common heterogeneous endocrine disorder characterized by irregular menses, hyperandrogenism, and polycystic ovaries [36]. Its clinical manifestations may include: menstrual irregularities, signs of androgen excess, and obesity. Insulin resistance and elevated serum Luteinizing Hormone levels are also common features in PCOS. PCOS is associated with an increased risk of type 2 diabetes and cardiovascular events [37]. A study by Kaur *et al* investigates PCOS using granulosa cells of 40 women discordant for PCOS undergoing in vitro fertilization [38]. Granulosa cell gene expression profiling was accomplished using Affymetrix Human Genome-U133 arrays. The samples were analyzed for differences in their transcript profile between PCOS and normal ovulatory women. Here, we obtained the raw data from GEO (GSE34526) and performed quality control and normalization using the Affymetrix quality control and pre-processing module of arrayanalysis.org [39]. All arrays were determined to be of sufficient quality for inclusion in further analysis. The quality control report generated by arrayanalysis.org has been provided (S1 Text). Statistical analysis was also performed in arrayanalysis.org using the statistical analysis module [40]. The gene level statistics have been provided (S2 Table). Over-representation analysis of the gene level statistics was performed in PathVisio using the combined collection of curated and reactome_approved pathways from WikiPathways. The Z score was calculated using the criterion absolute log2 fold change > 1 and P. value < 0.05. This criterion is often used for detecting differentially expressed genes in genomics datasets for the explicit purpose of exploratory pathway enrichment analysis [41–43].

**Fig 4:**
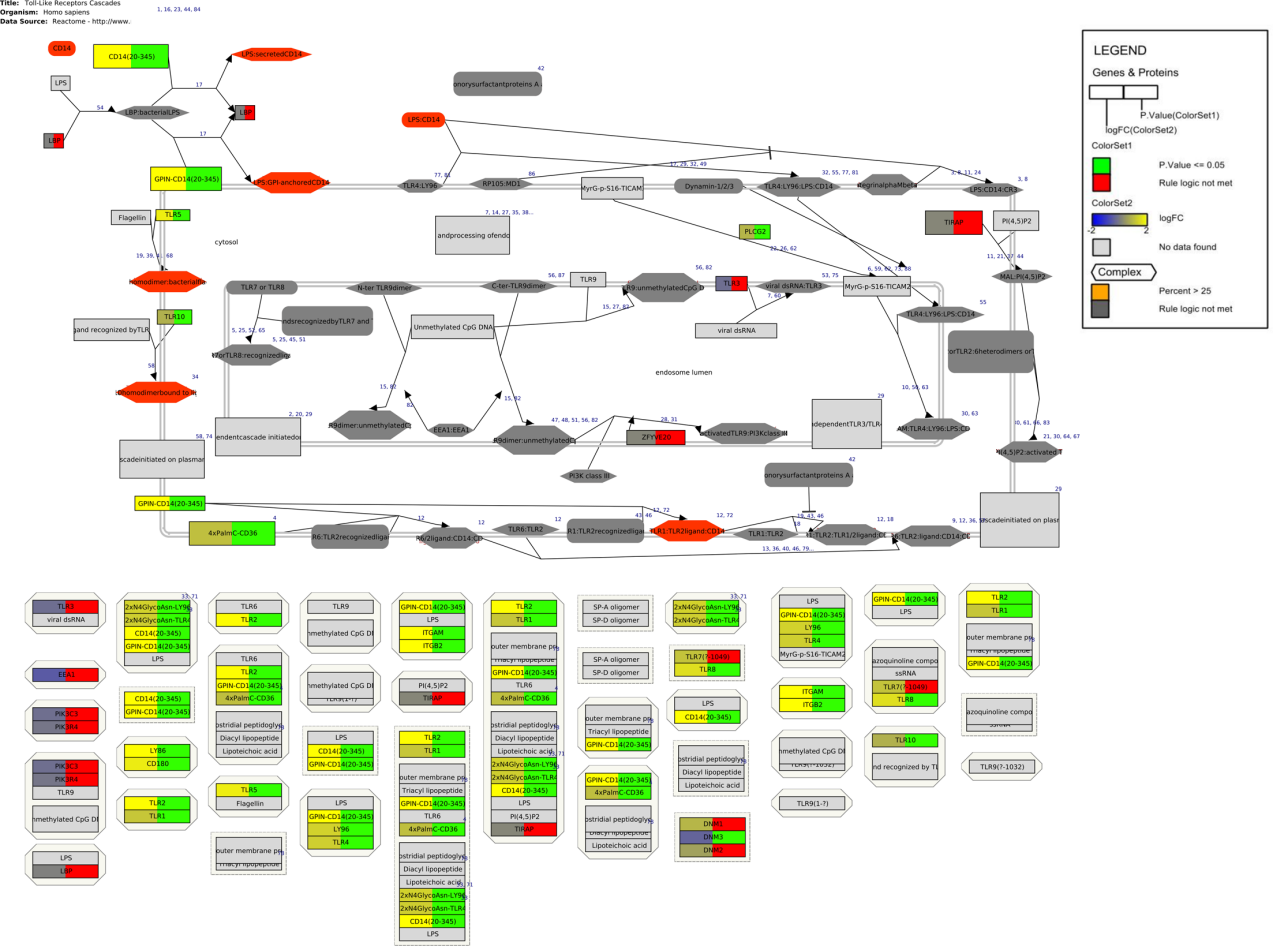
Human granulosa cells gene expression in normal ovulatory versus PCOS women visualized on the Toll-Like Receptors Cascades pathway (WP2775_r83597) in PathVisio [44]. Human granulosa cells were isolated from ovarian aspirates of normal ovulatory and PCOS women undergoing IVF. For each sample, RNA was extracted and hybridized to an Affymetrix Gene Chip. Genes not measured appear in gray. The log fold change (logFC) is depicted with a blue to yellow color gradient corresponding to the values -2 to 2. Significant genes with a P.value < 0.05 are marked in green and the rest in red. Significant complexes with a score > 25 are marked in orange and the rest in dark gray.

Table 2 shows the top ten pathways obtained through pathway analysis. 2 pathways from WikiPathways and 8 from Reactome show up in the list. The Toll-Like Receptors Cascades pathway (Fig 4) from Reactome shows up as the most affected pathways with a Z score of 7.25. Subsequently, the gene statistics were visualized on the pathway. The logFC values were visualized using a color gradient: blue to yellow, corresponding to the value -2 to 2. The P.value was visualized using a color rule, the genes with “P.value < 0.05” were marked in green and the rest red. The ComplexViz plugin was used to score the complexes on the pathway to highlight the complexes of interest. The same criteria “P.value < 0.05” was used to calculate the percentage scores for the complexes. The scores were then visualized on the pathway. Complexes with a percent score higher than 25 were marked in orange and the rest were colored dark grey.

**Table 2:** Table showing the top ten pathways obtained performing over-representation analysis in PathVisio.

Pathway	Z score	Source
Toll-Like Receptors Cascades	7.25	Reactome
Cell surface interactions at the vascular wall	7.09	Reactome
Immunoregulatory interactions between a Lymphoid and a non-Lymphoid cell	7	Reactome
IL1 and megakaryotyces in obesity	6.71	WikiPathways
Interferon gamma signalling	6.3	Reactome
Signal regulatory protein (SIRP) family interactions	5.17	Reactome
Costimulation by the CD28 family	4.82	Reactome
IL-4 Signaling Pathway	4.33	WikiPathways
Interleukin-3, 5 and GM-CSF signalling	4.25	Reactome
Syndecan interactions	4.07	Reactome

### Network analysis and visualization in Cytoscape

The biological entities in pathways and their relationships can be represented as nodes and edges in abstract biological networks. This opens up a large variety of network analysis methods to further extend, analyze and visualize biological pathways.

The incorporation of the WikiPathways and ReactomeFIViz apps in the Cytoscape framework allows further investigation of biological pathways using a wide variety of Cytoscape apps for network analysis and visualization. The visualization of an example Reactome pathway with both apps is provided (S3 Fig).

## Discussion

The WikiPathways project has developed a suite of pathway visualization and editing tools for users to view and edit pathways, and established a dynamic community to continuously crowd source updates and novel pathway content. The contents in Reactome are created by select domain experts in target fields of research with Reactome editorial staff. Including Reactome content has significantly expanded the coverage of pathway information at WikiPathways. Likewise, incorporating community edits from the WikiPathways versions of Reactome content significantly expands their pool of contributors, helping them produce more frequent updates and create links to outside databases. In the current implementation, we use a notification mechanism developed in WikiPathways to send edits from WikiPathways to Reactome. However, such an approach cannot be scaled up if many edits occur in the WikiPathways web site. We plan to develop a robust round-trip software approach in the Reactome curator tool so that edits in WikiPathways for Reactome pathways can be imported into the Reactome database easily. Such a tool will find new edits, and then present them to Reactome curators in graphical user interfaces so that curators can decide whether or not these edits should be committed into the Reactome database. We believe a true round-trip approach between Reactome and WikiPathways will benefit both projects, and set an example for other projects to collaborate with each other.

The conversion of Reactome pathways to the GPML format enables the analysis and data visualization of Reactome pathways in PathVisio. PathVisio is a widely used pathway analysis software, preferred due to its excellent data visualization capabilities as demonstrated by its use in numerous academic publications [45–49]. PathVisio allows multiple data points to be visualized on one node using colors and color gradients permitting easy visualization of time series data. The data visualized images can then be exported as images for further publication or in html format as a mini-website to easily maneuver the uploaded data on the pathway image. The new ComplexViz plugin simplifies analysis of the converted Reactome pathways. As Reactome pathways typically contain numerous complexes, the plugin enables highlighting complex components on the bottom of the pathway diagram and the other way around. It also enables browsing complex components in a side panel and visualizing data uploaded for the complex components on the parent complex node. This highlights the complexes of interest, which can then be further studied. The complex component diagram on the side panel also displays the data uploaded thereby making it simpler to look at them without having to look for them on the bottom of the pathway.

The pathway analysis case study presented here with a transcriptomics dataset comparing women with normal ovulatory physiology with those with PCOS shows that addition of the Reactome pathway set clearly improves pathway analysis results. The list of top ten most affected pathways feature pathways from both the curated and reactome_approved collection of pathways from WikiPathways. More pathways appear from the reactome_approved collection, which is expected since the collection is manually curated. The reactome_approved collection adds 4417 new gene products and 500 new metabolites. However, the curated collection still contains 1414 unique gene products and 2360 unique metabolites. There are 3438 gene products and 325 metabolites in common between the two collections. Therefore, the conversion adds content without much overlap.

The toll like receptor (TLR) cascades pathway, which shows up as the most changed pathway in this condition is from the Reactome collection. TLRs are an important family of pattern recognition receptors (PRR) involved in innate immunity. The innate immune system initiates an inflammatory response after recognizing pathogens by PRRs [50]. Emerging evidence suggests that PCOS is associated with systemic inflammation [51, 52]. Furthermore, various studies have reported that TLRs are expressed in the female reproductive tract[53]. Therefore, this pathway is clearly interesting for PCOS. In addition, the Cell surface interactions at the vascular wall pathway, which is the second most highly affected pathway is also from the Reactome collection. This pathway is annotated with the Gene Ontology biology process term, leukocyte migration. Since PCOS is associated with elevated levels of circulating leukocytes [54], this pathway is clearly of interest. Both pathways, are from the newly converted collection of pathways from Reactome and clearly add biological knowledge, as illustrated with the case study for PCOS.

The availability of Reactome pathways in WikiPathways allows the analysis of the pathways with several new analysis tools. Besides the analysis of pathways in PathVisio, users can also use the WikiPathways app for Cytoscape to analyze the pathways as biological networks. While the ReactomeFIViz app focuses on functional gene interaction networks, the WikiPathways app creates a representative network of the pathway including metabolites and other pathway elements. Consequently the created network provides a new analysis tool that allows the integrated analysis of different omics datasets.

## Methods

### Reactome converter

A Java based format converter has been developed to convert pathways from the Reactome database to the WikiPathways format. Pathways in Reactome are stored in a relational database with their diagrams encoded in XML strings, while pathways in WikiPathways are stored as GPML, which is an XML based file format. In addition, both repositories have Java based APIs according to which the pathway files can be read and written. These internal data models of the two databases are used to read and write the pathways obtained from them. This allows the converter to remain flexible and backwards compatible as long as the data models themselves are. This also makes the converter stable through version updates of pathways as long the pathways are organized according to the same model. The conversion is done in the following steps: (i) Creating a GPML pathway and adding pathway attributes, (ii) Converting the pathway elements, and (iii) Annotating the pathway and pathway elements. These steps are described further below. The converter is open source and the code is available from the GitHub repository [55].

#### Step (i): Creating a GPML pathway

A Reactome pathway combined with its rendering information is read from the database via the Reactome Java API [56]. A new GPML pathway is created by instantiating the pathway class and pertinent information is added to the GPML. This information consists of the data source (Reactome), the Reactome version, the organism for which the pathway has been drawn, e.g. Homo sapiens. Biologists who have drawn the pathway are added as authors, the Reactome team members who have edited the pathway are added as maintainers, along with their email addresses.

#### Step (ii): Converting the pathway elements

**Fig 5:**
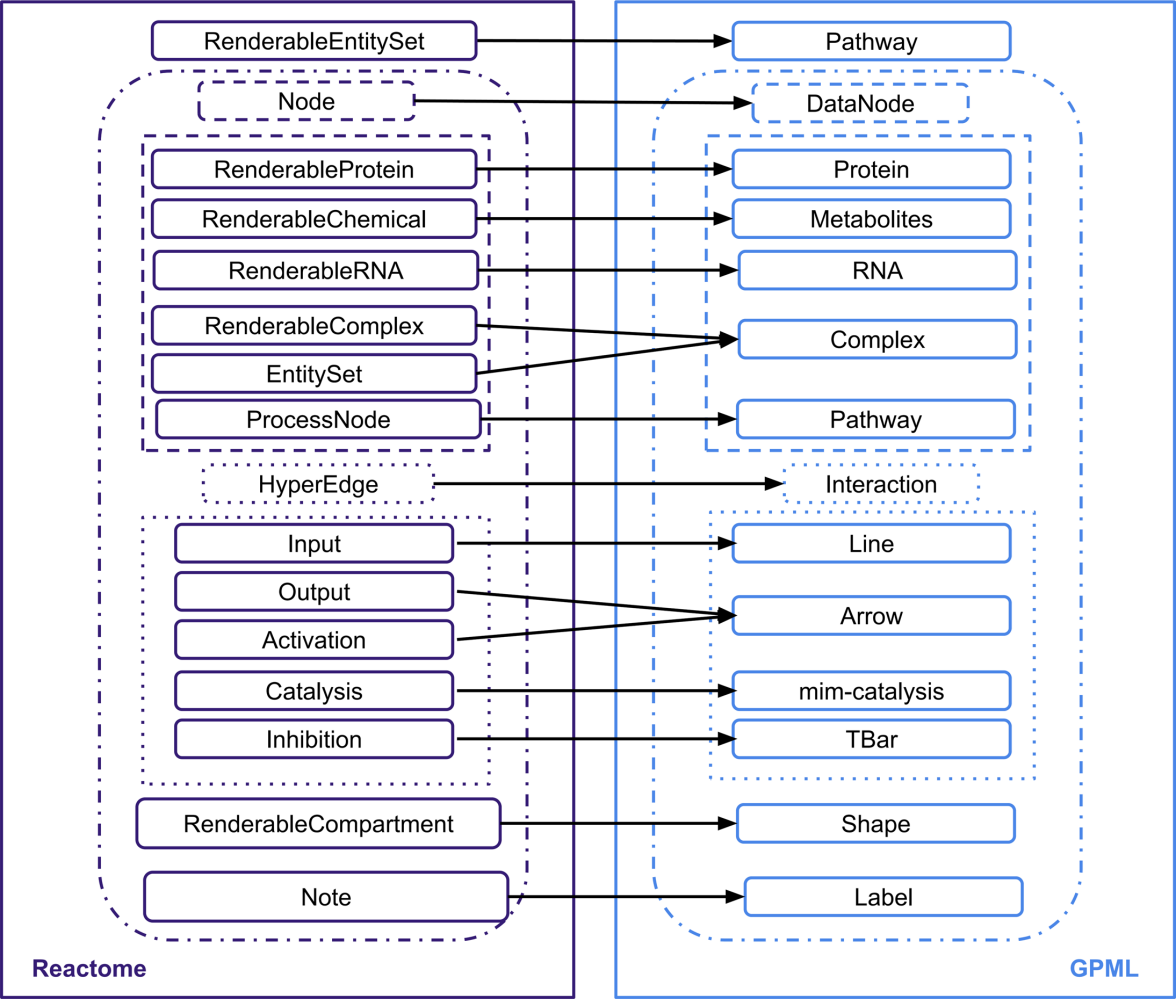
Conversion of Reactome Java data classes to corresponding WikiPathways Java data classes.

Each entity in the Reactome pathway is converted to the corresponding WikiPathways element. Fig 5 presents an overview of mapping of the elements of a Reactome pathway to the corresponding WikiPathways pathway elements. Nodes are converted to DataNodes. Individual node types, such as Proteins, Small molecules, RNAs, Process Nodes are converted to the corresponding WikiPathways elements, namely Protein, RNA, Metabolite, and Pathway. Complexes and entity sets in Reactome are converted to Group in WikiPathways, with the styles “complex” and “group” respectively. The components of the complexes and entity sets are obtained and these are added to the bottom of the pathway diagram. Duplicates are not displayed on the pathway diagram for keeping the pathway diagram concise but are maintained in the GPML and showed in the “Properties” side panel of PathVisio. Compartments from the Reactome Pathway are converted into a group in WikiPathways. Notes in the Reactome pathway are converted to Labels.

**Fig 6:**
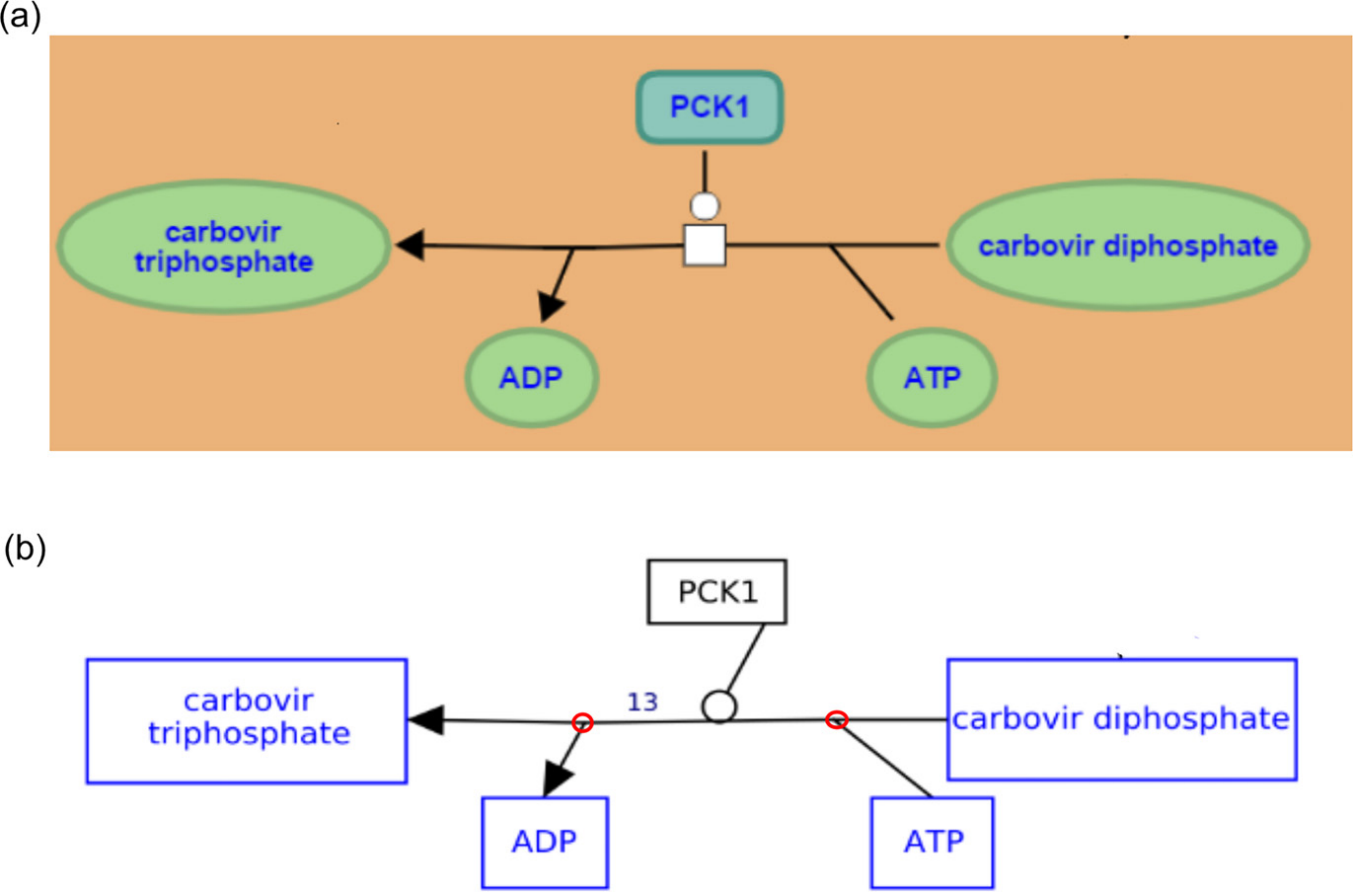
A comparative view of hyperedges in a Reactome pathways and how they are converted in WikiPathways. A hyperedge from the Abacavir transport and metabolism pathway is shown. (a) Reactome View (b) WikiPathways view, the anchors are highlighted.

In Reactome, the reactions known as hyper edges are modeled such that there is a backbone reaction to which the inputs, outputs, catalysts, activators, and inhibitors are connected. Each branch of the hyper edge (inputs, outputs, catalysts, inhibitors, activators) is converted into a GPML interaction and connected to a backbone interaction using anchors; this achieves the same SBGN compliant reaction view for all substrates, products, enzymes, activators, and inhibitors in GPML (Fig 6).

#### Step (iii): Annotating the pathway and pathway elements

Subsequently, the Reactome pathway object is mined for annotations for the different elements. Preferably, the proteins are annotated with UniProt identifiers and the metabolites with ChEBI identifiers, in absence of the preferred annotation Reactome identifiers are used. Interactions, Complexes, and Pathways are annotated with Reactome identifiers. All pathway elements are also annotated with literature references using PubMed identifiers.

### Calculating coverage of biological entities

Human GO Terms were downloaded from UniProt-GOA [57]. Scripts in Java were written to parse the document to obtain the GO identifiers and identifiers of the terms for the three structured ontologies that describe gene products in terms of their associated biological processes, cellular components and molecular functions. The current release version 3.6 of HMDB was downloaded to obtain the superset of all human metabolites. Additional scripts in Java were written to map all gene products in the two pathway collections to Ensembl and all metabolites to HMDB. All the scripts used are available from GitHub [58]. The Ensembl gene identifiers were mapped to GO terms using Ensembl BioMart [59], to obtain the total GO term coverage of the two pathway collections and also individual coverage of each GO category. The R package gplots was then used to create Venn diagrams showing GO and HMDB coverage [60]. The Venn diagrams were manually updated in PowerPoint.

### ComplexViz plugin

The newly developed plugin improves visualization of data on complexes and their components. The plugin can be installed in PathVisio using the plugin manager and adds a side panel “Components”. The top half of this panel displays the components of the complex that is clicked as a mini pathway diagram. Imported data is visualized both on the main pathway diagram and on the “Components” side panel containing the complex component diagram. Clicking on the buttons in the side panel next to the mini pathway diagram, displays the cross-references and expression data available for that data node on the bottom half of the panel. The plugin also adds the submenu item “Complex Visualization” to the Data menu. Clicking it opens a dialog box for setting visualization options for complexes. Three visualization options have been implemented. These methods allow changing the border color of complex and components, coloring complex nodes according to a calculated ratio, and drawing the complex label. Users can select a border color for complexes and their components to indicate which complex and components belong together. Complexes can be colored based on the percentage of complex components that qualify the user defined criterion. This percentage is calculated for all complexes on the pathway. Color gradients or rules can be used to visualize the score on the complexes. Text labels can be drawn on the Complexes after data has been visualized, the font and size of text of the label can be changed. The plugin is open source and the code is available from the GitHub repository [61]. A detailed user guide is provided (S2 Text). An up-to-date copy will be maintained at the GitHub wiki [62].

## Supporting Information

S1 Table Pathway databases in PathGuide. Access and data availability.
(XLSX)

S2 Table Gene level statistics. Obtained from the statistical analysis module of arrayanalysis.org.
(XLSX)

S1 Fig A comparative view of the GPCR ligand binding pathway for Homo sapiens from Reactome Database (version 54). (a) Reactome View of GPCR ligand binding pathway (http://www.reactome.org/PathwayBrowser/#DIAGRAM=500792) and (b) Pathway view on WikiPathways (http://wikipathways.org/instance/WP1825).
(PDF)

S2 Fig Venn diagrams showing the coverage of the three GO categories biological process, cellular component, and molecular function by WikiPathways and Reactome individually. (PDF)

S3 Fig Visualization of a Reactome pathway in Cytoscape. (a) using the WikiPathways app and (b) using the ReactomeFIViz app.
(PDF)

S1 Text Quality control report. Obtained from the Affymetrix quality control and pre-processing module of arrayanalysis.org.
(PDF)

S2 Text User guide for the ComplexViz plugin. This guide describes the functionalities of the ComplexViz plugin and how it can be used in PathVisio.
(PDF)
