# Supplementary figures and images for: Reactome from a WikiPathways Perspective
Source: PLoS Comput Biol. 2016 May 20;12(5):e1004941. doi: 10.1371/journal.pcbi.1004941 (PMC4874630; doi:10.1371/journal.pcbi.1004941)

(A) GPCR ligand binding (<http://www.reactome.org/PathwayBrowser/#DIAGRAM=500792>)

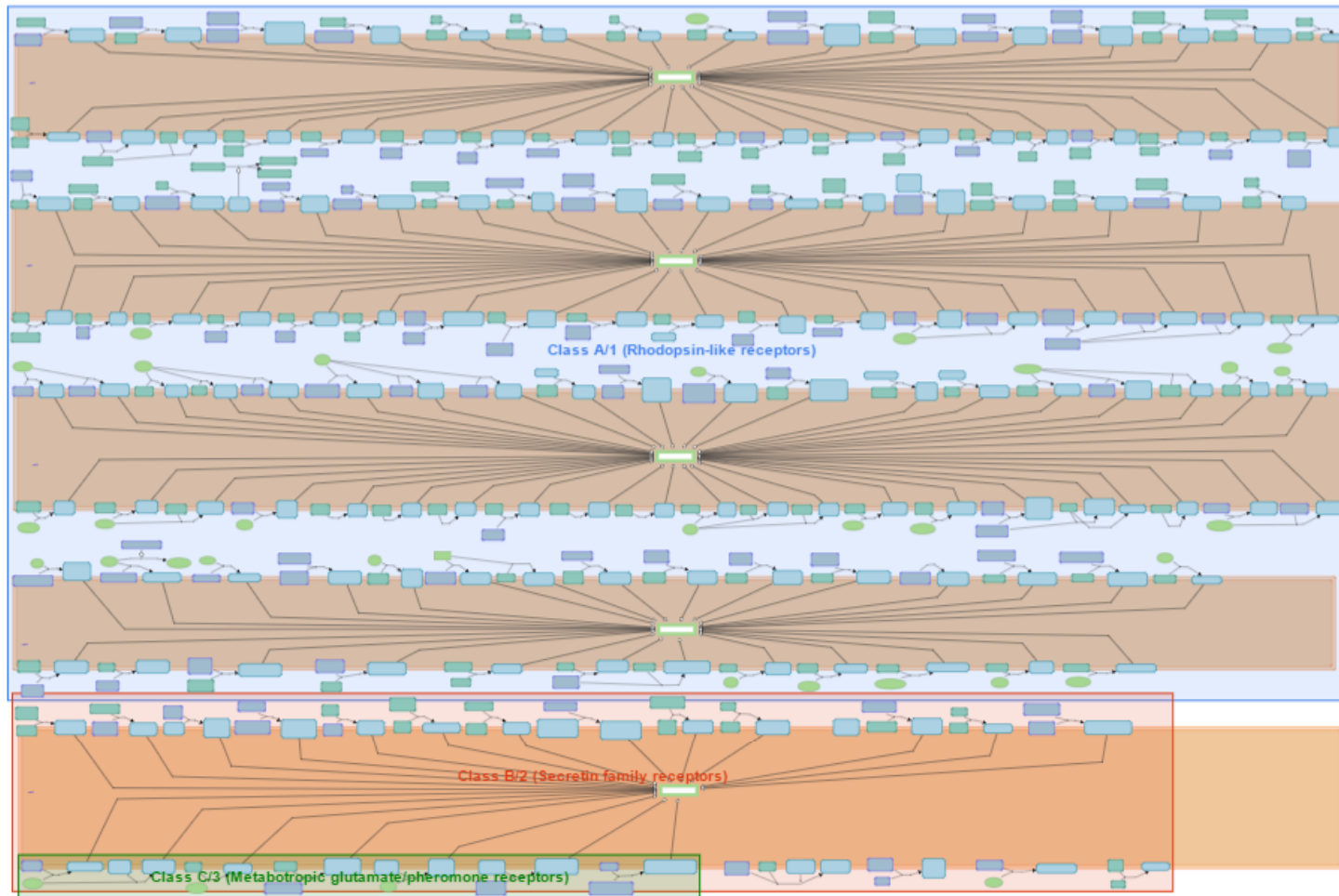

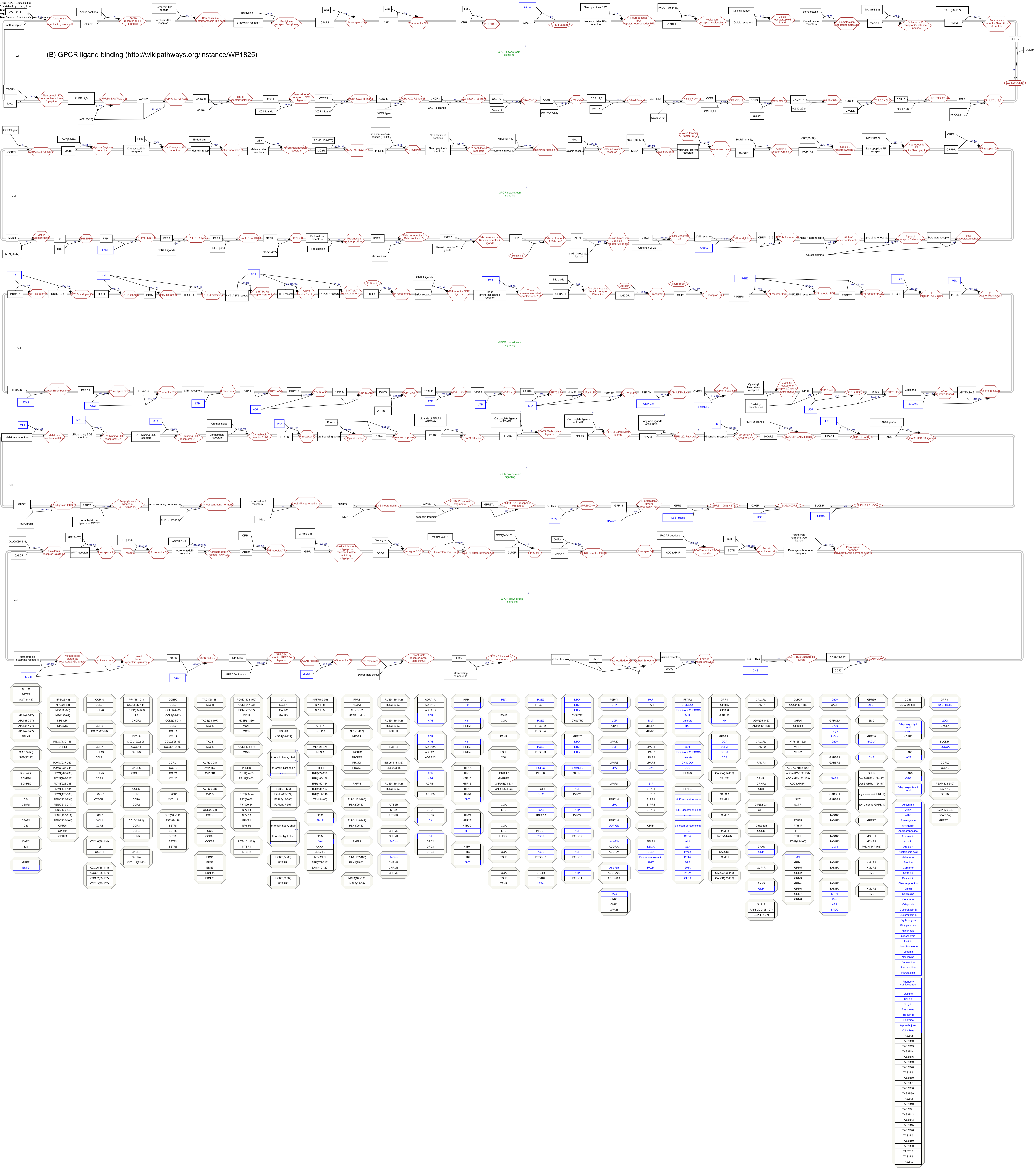

Supplement: S1 Fig — (a) Reactome View of GPCR ligand binding pathway (http://www.reactome.org/PathwayBrowser/#DIAGRAM=500792) and (b) Pathway view on WikiPathways (http://wikipathways.org/instance/WP1825). (PDF) [file pcbi.1004941.s003.pdf]
