# Supplementary material for: Reactome from a WikiPathways Perspective
Source: PLoS Comput Biol. 2016 May 20;12(5):e1004941. doi: 10.1371/journal.pcbi.1004941 (PMC4874630; doi:10.1371/journal.pcbi.1004941)
Supplement: S2 Fig — (PDF) [file pcbi.1004941.s004.pdf]

This figure has been included in the main manuscript as Fig.3(a)

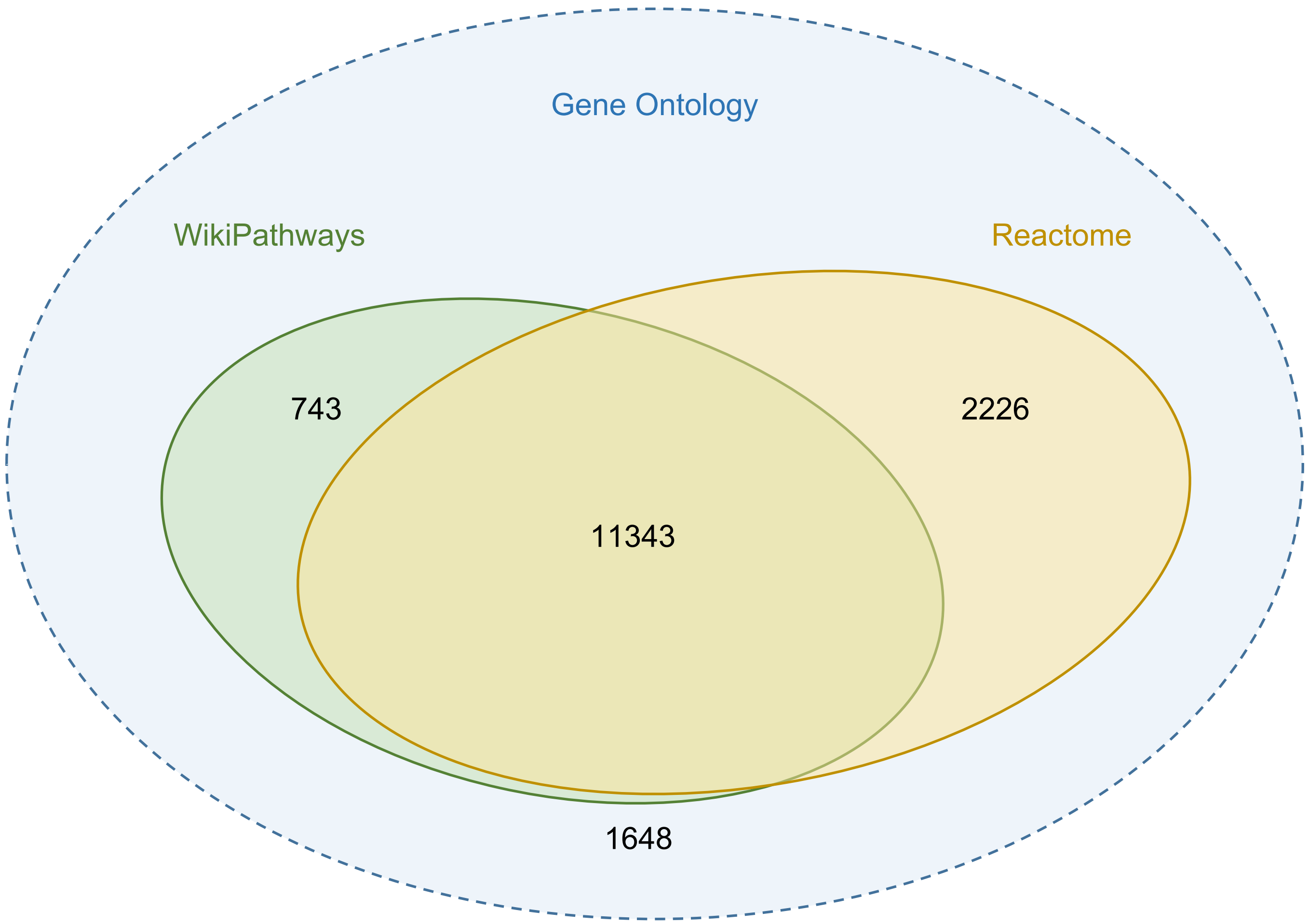


**Fig.1.**Venn Diagram showing coverage of Gene Ontology Terms by gene products of curated collection and reactome_approved collection of pathways from WikiPathways.

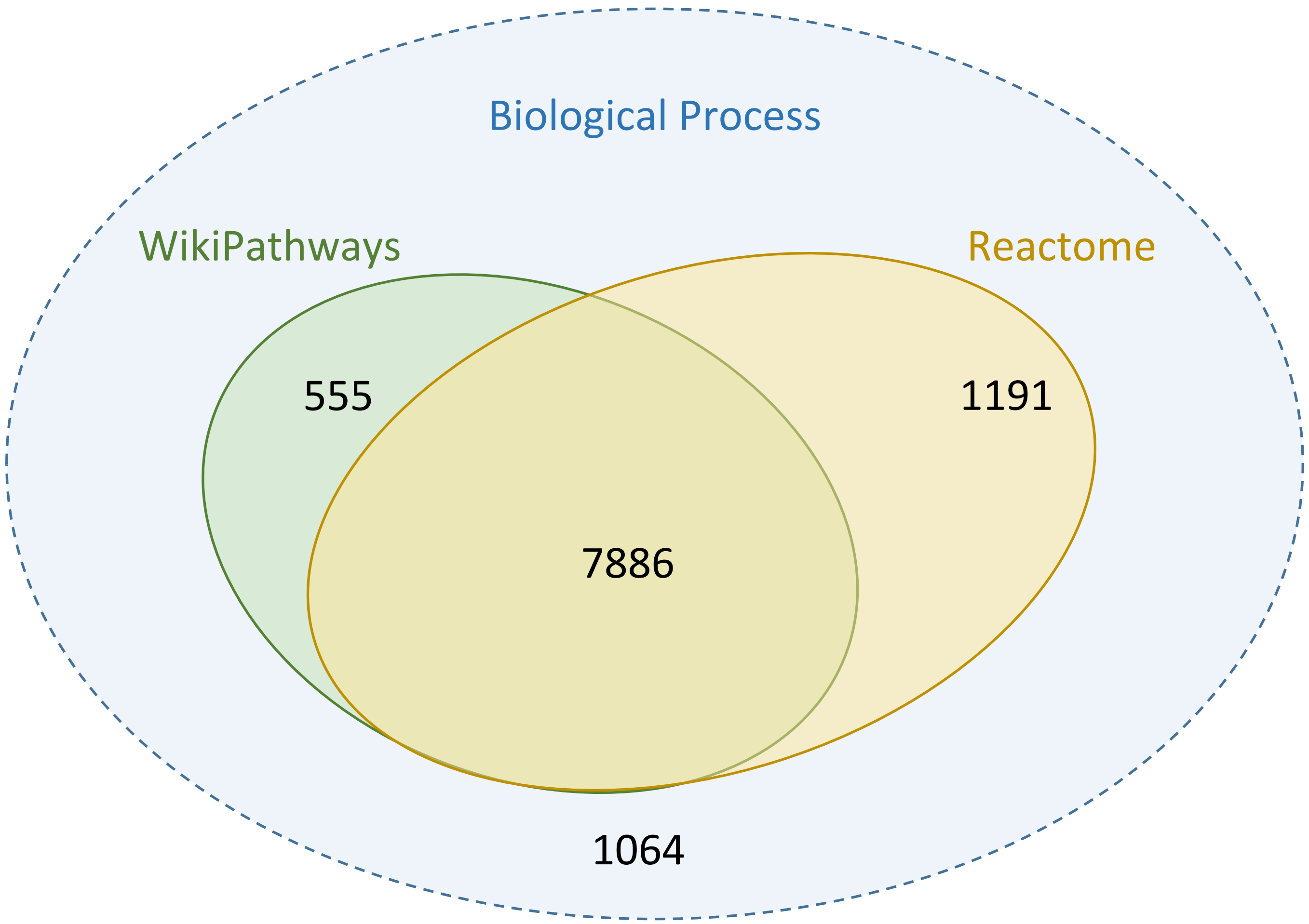


**Fig.2.** Venn Diagram showing coverage of terms from the Biological Process branch of Gene Ontology by gene products of curated collection and reactome_approved collection of pathways from WikiPathways.

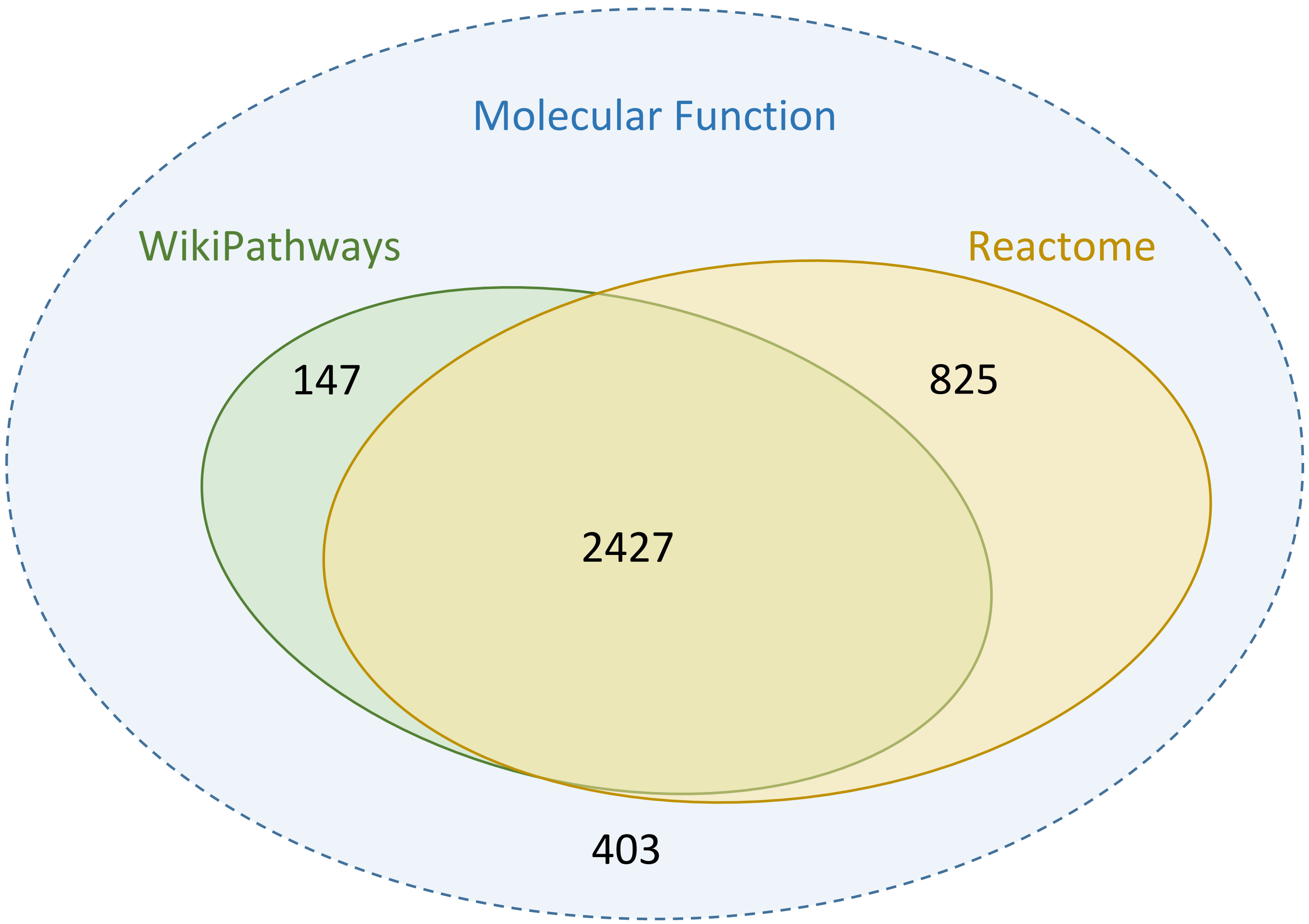


**Fig.3.** Venn Diagram showing coverage of terms from the Molecular Function branch of Gene Ontology by gene products of curated collection and reactome_approved collection of pathways from WikiPathways.

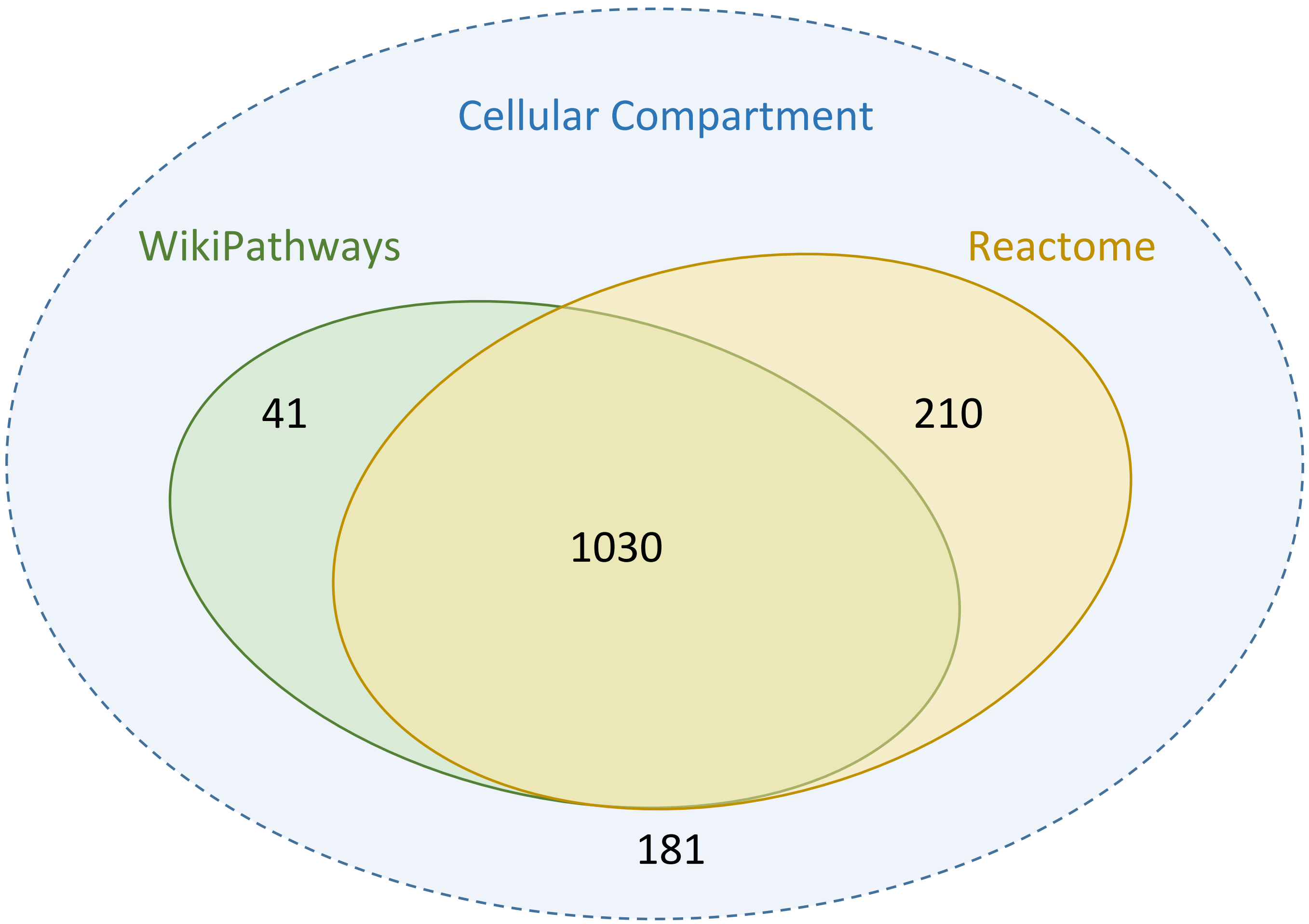


**Fig.4.** Venn Diagram showing coverage of terms from the Cellular Compartment branch of Gene Ontology by gene products of curated collection and reactome_approved collection of pathways from WikiPathways.

This figure has been included in the main manuscript as Fig.3(b)

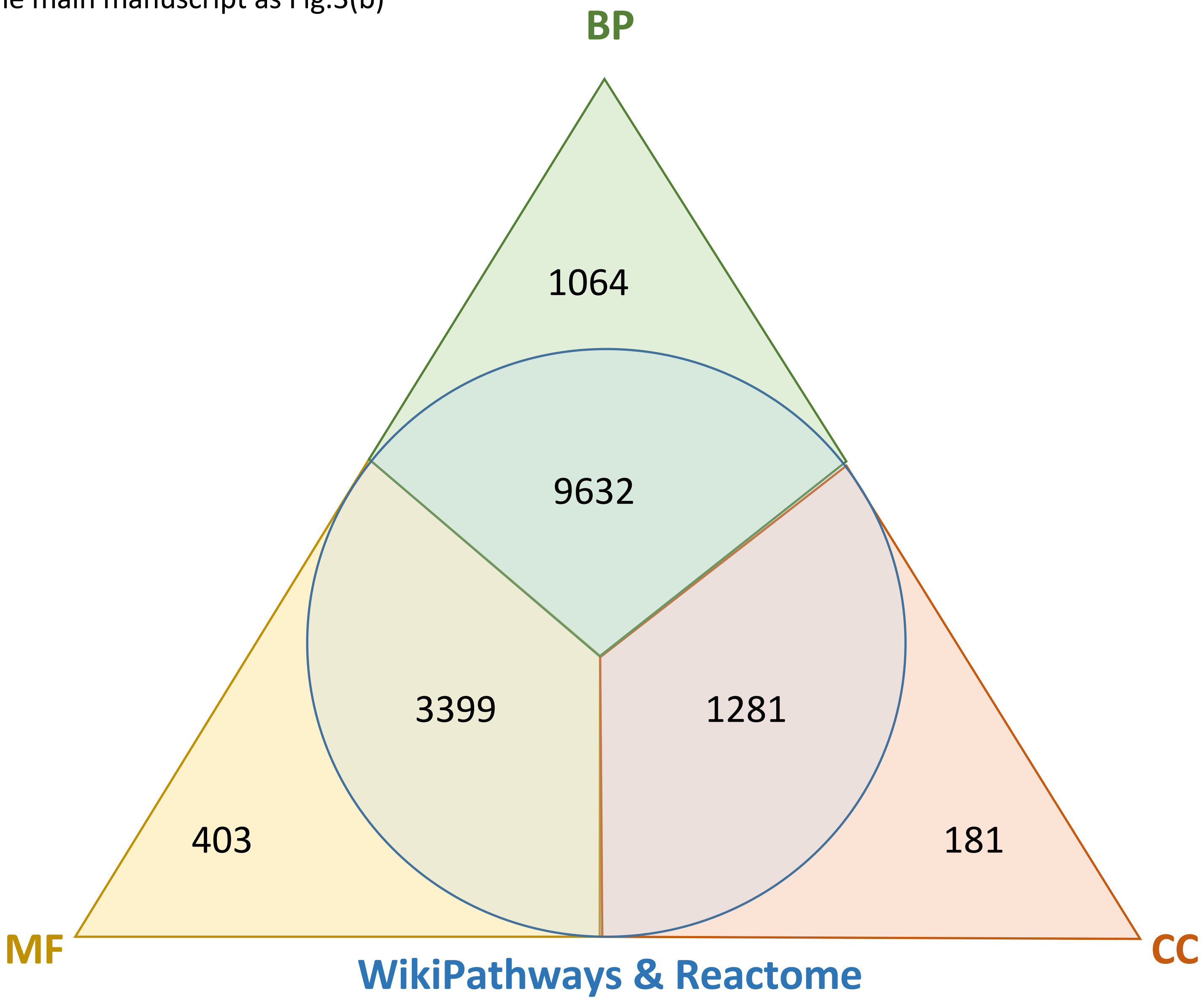


**Fig.5.** Venn Diagram showing coverage of terms from the Biological Process (BP), Molecular Function (MF), and Cellular Compartment (CC) branches of Gene Ontology by gene products of curated collection and reactome_approved collection of pathways from WikiPathways.

This figure has been included in the main manuscript as Fig.3(c)

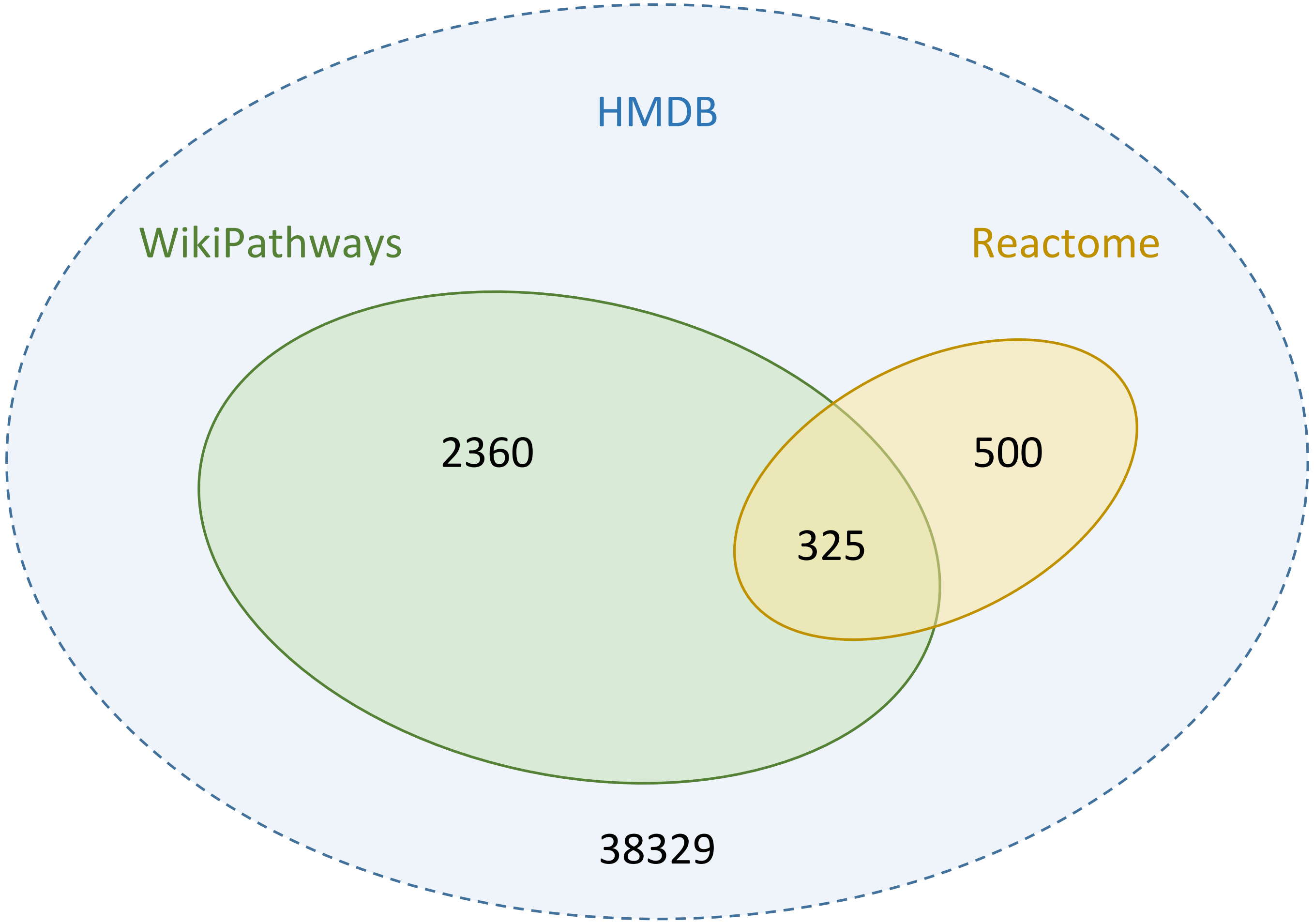


**Fig.7.**Venn Diagram showing coverage of the Human Metabolome Database (HMDB) by metabolites of curated collection and reactome_approved collection of pathways from WikiPathways.
