# Supplementary material for: Reactome from a WikiPathways Perspective
Source: PLoS Comput Biol. 2016 May 20;12(5):e1004941. doi: 10.1371/journal.pcbi.1004941 (PMC4874630; doi:10.1371/journal.pcbi.1004941)
Supplement: S3 Fig — (a) using the WikiPathways app and (b) using the ReactomeFIViz app. (PDF) [file pcbi.1004941.s005.pdf]

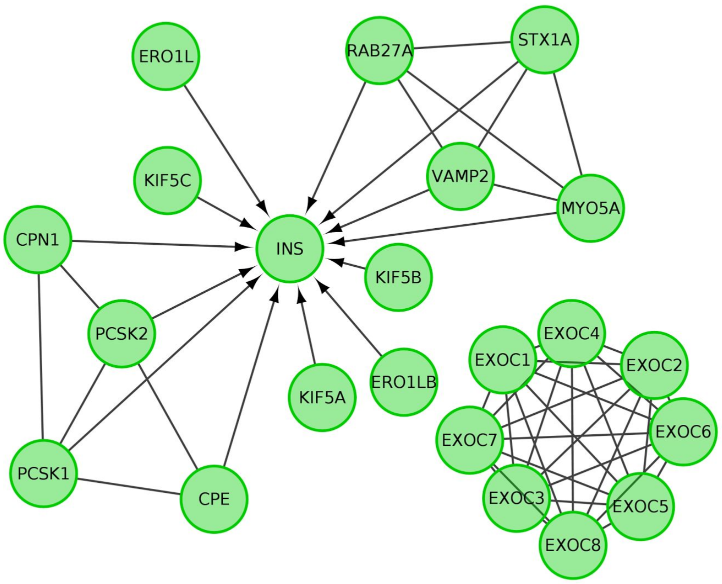

S3 Fig A: *ReactomeFIViz app for Cytoscape* view of the "Insulin Processing" pathway (http://wikipathways.org/instance/WP2736_r81765) - Functional Interaction (FI) network created using ReactomeFIViz app (http://apps.cytoscape.org/apps/reactomefiplugin) - version 4.2.0beta

---

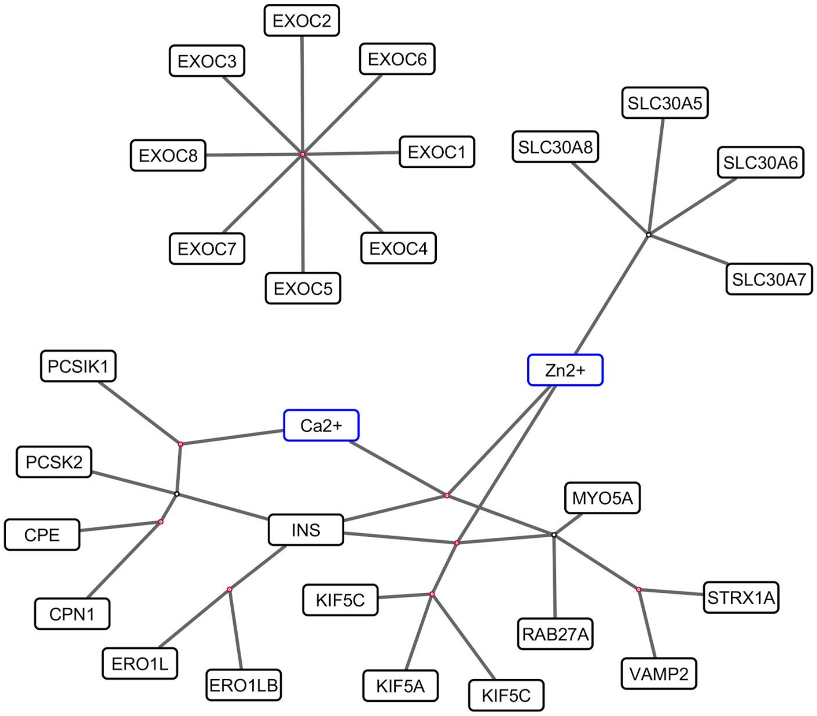

S3 Fig B: *WikiPathways app for Cytoscape* view of the "Insulin Processing" pathway (http://wikipathways.org/instance/WP2736_r81765) - network view. WikiPathways app (http://apps.cytoscape.org/apps/wikipathways) - version 3.3.0beta
