# Supplementary material for: Reactome from a WikiPathways Perspective
Source: PLoS Comput Biol. 2016 May 20;12(5):e1004941. doi: 10.1371/journal.pcbi.1004941 (PMC4874630; doi:10.1371/journal.pcbi.1004941)
Supplement: S1 Text — Obtained from the Affymetrix quality control and pre-processing module of arrayanalysis.org. (PDF) [file pcbi.1004941.s006.pdf]

Quality Control & Pre-processing Evaluation

of
CEL_files_0

REPORT

Array names and grouping

ArrayDataFile	SourceName	FactorValue
GSM850527_RSN-11.CEL.gz	Array1	Control
GSM850528_RSN-12.CEL.gz	Array2	Control
GSM850529_RSN-13.CEL.gz	Array3	Control
GSM850530_RSN-10.CEL.gz	Array4	PCOS
GSM850531_RSN-14.CEL.gz	Array5	PCOS
GSM850532_RSN-16.CEL.gz	Array6	PCOS
GSM850533_RSN-17.CEL.gz	Array7	PCOS
GSM850534_RSN-2.CEL.gz	Array8	PCOS
GSM850535_RSN-4.CEL.gz	Array9	PCOS
GSM850536_RSN-5.CEL.gz	Array10	PCOS

Summary of raw data quality indicators

blue = "within" / red = "out of" recommended cut-off

	3'/5' beta-actin (cutoff=3)	3'/5' GAPDH (cutoff=1.25)	Hybridization BioB<BioC<BioD<CreX	Hybridization BioB=Present	Percent Present spread<=10%	Background spread<=20%	Log Scale Factor spread<=3
Array1	1.43	**1.57**	T	P	**36 %**	39	1.76
Array2	1.2	1.07	T	P	49 %	38	0.17
Array3	1.2	1.08	T	P	46 %	41	0.48
Array4	1.11	1.16	T	P	43 %	43	0.83
Array5	1.15	1.1	T	P	50 %	36	0.24
Array6	1.08	1.05	T	P	45 %	45	0.89
Array7	1.1	1.08	T	P	49 %	40	0.43
Array8	1.08	1.04	T	P	42 %	32	2.39
Array9	1.04	1	T	**A**	**35 %**	47	2.01
Array10	1.04	1	T	P	**35 %**	49	2.21

(Bold = shown in red, "out of" recommended cut-off; all other values shown in blue.)

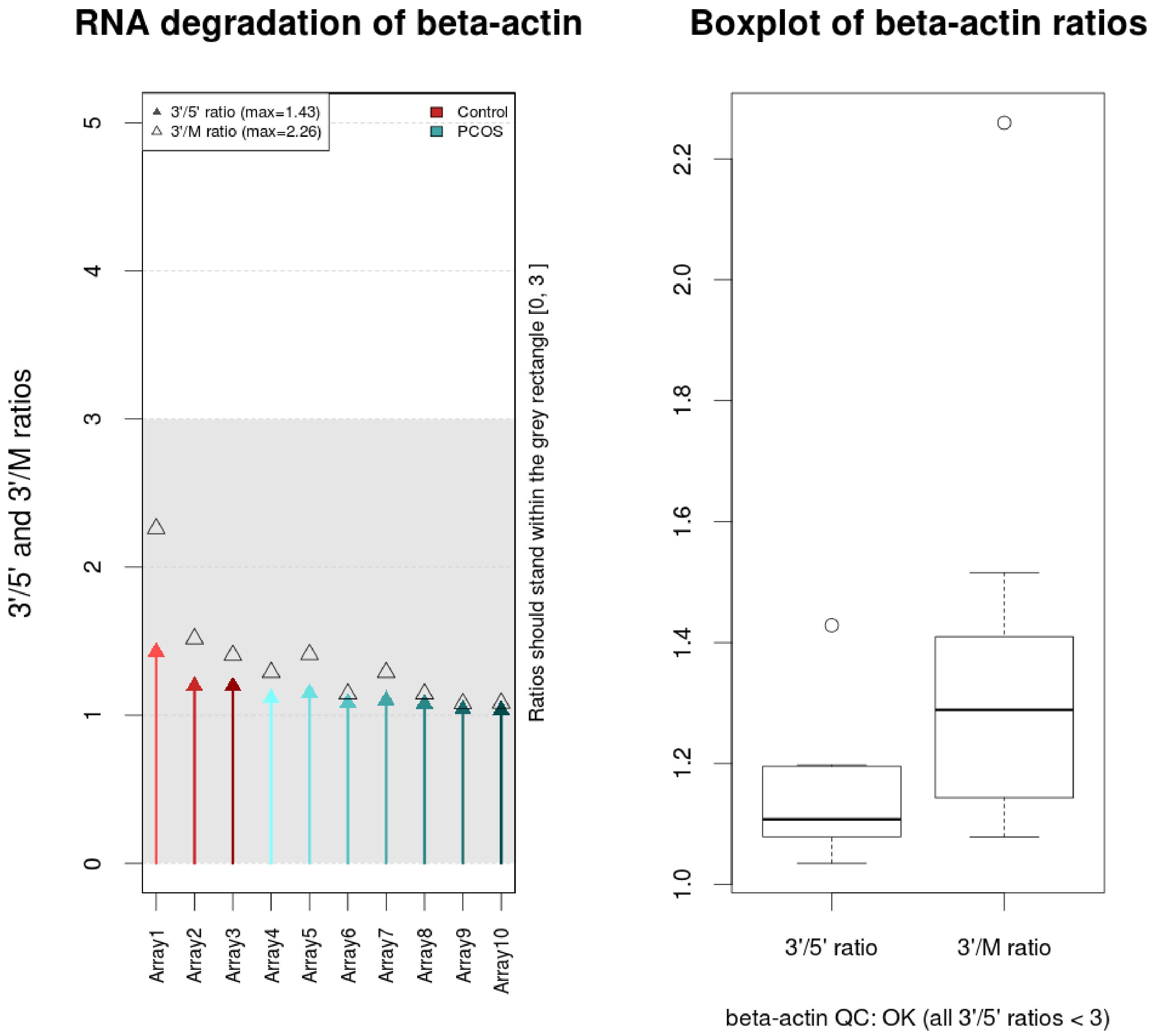
RNA degradation of beta-actin
Boxplot of beta-actin ratios
3'/5' ratio (max=1.43)
3'/M ratio (max=2.26)
Control
PCOS
3'/5' and 3'/M ratios
Ratios should stand within the grey rectangle [0, 3]
0
1
2
3
4
5
Array1
Array2
Array3
Array4
Array5
Array6
Array7
Array8
Array9
Array10
1.0
1.2
1.4
1.6
1.8
2.0
2.2
3'/5' ratio
3'/M ratio
beta-actin QC: OK (all 3'/5' ratios < 3)

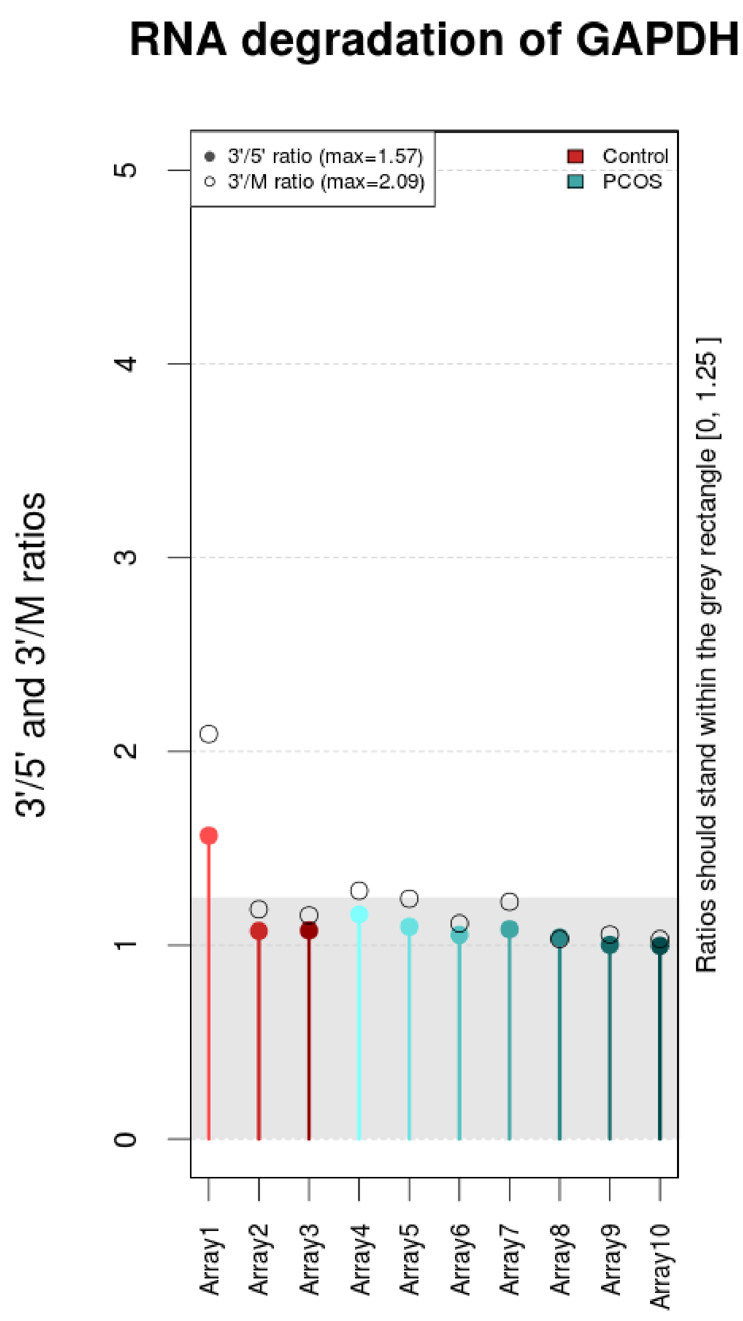

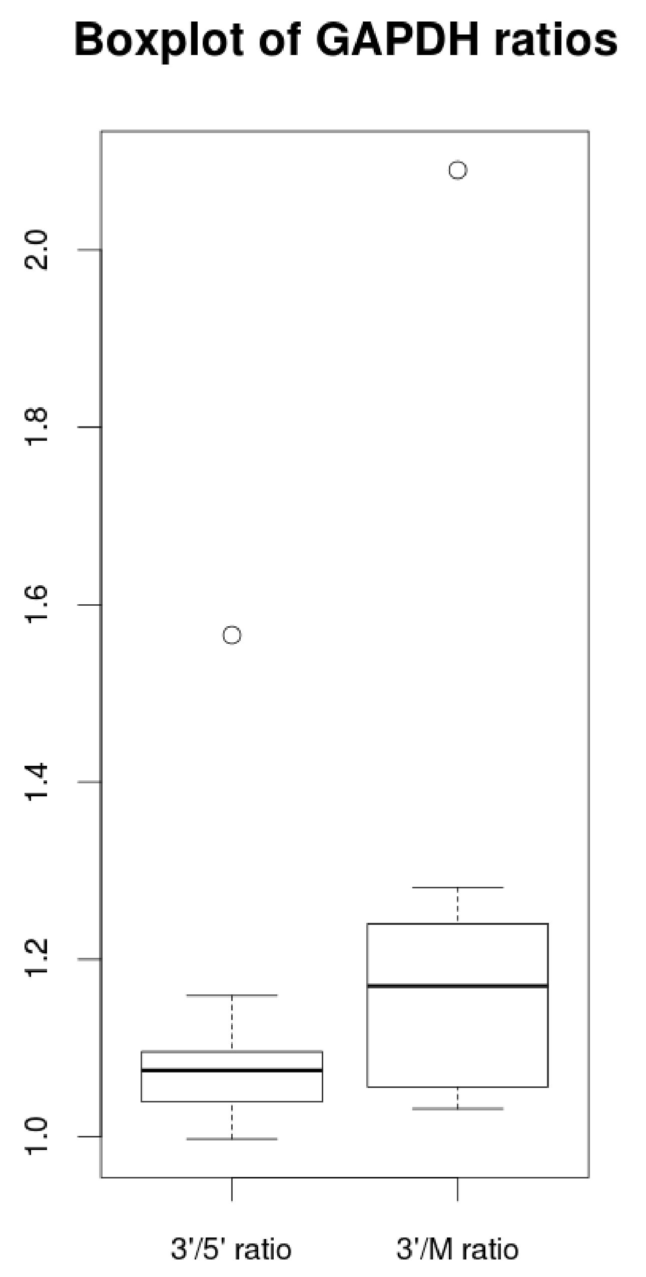

GAPDH QC: not OK (some 3'/5' ratios >1.25)
Note that the threshold of 1.25
was determined for Homo Sapiens.

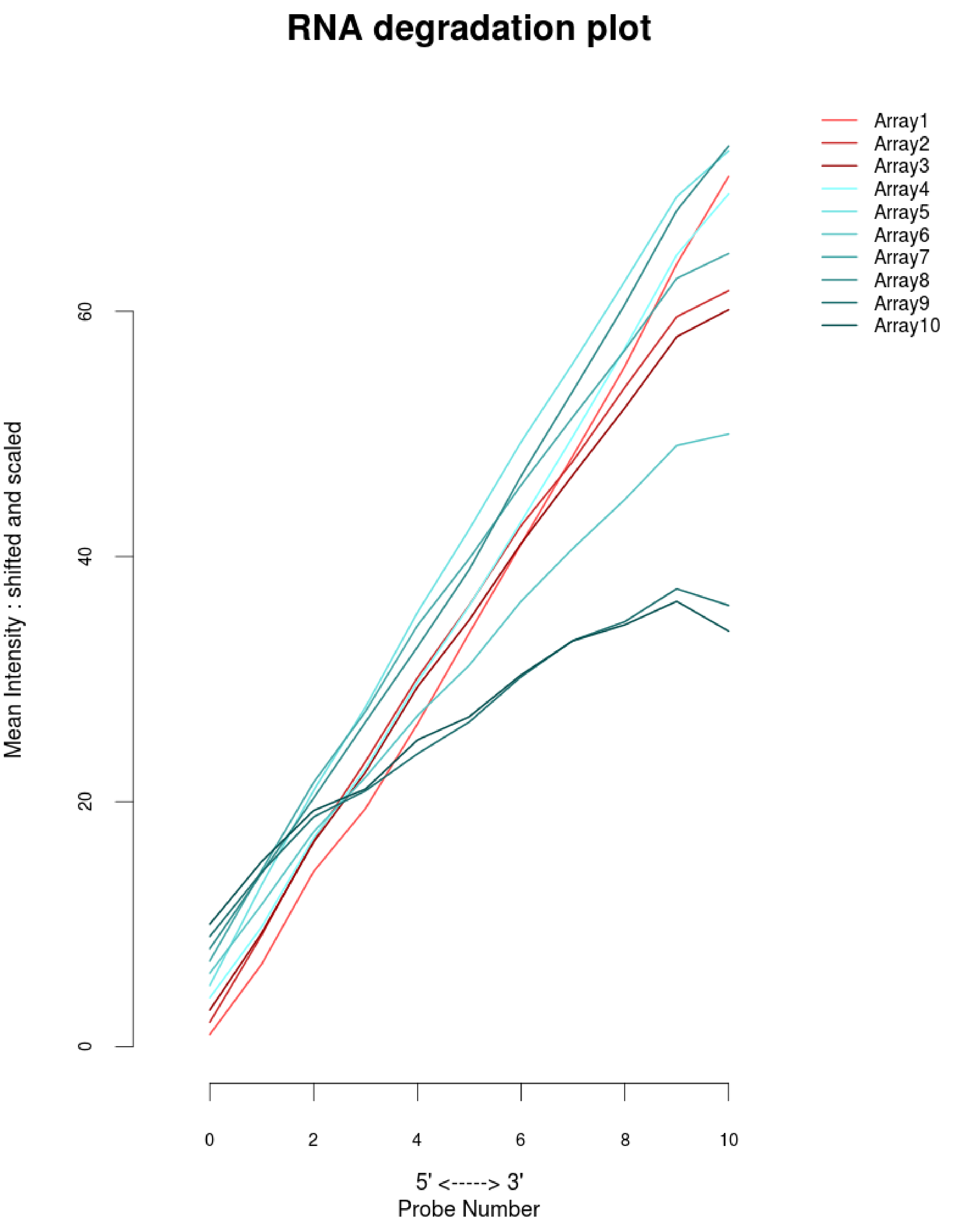
RNA degradation plot
Array1
Array2
Array3
Array4
Array5
Array6
Array7
Array8
Array9
Array10
Mean Intensity : shifted and scaled
0
20
40
60
0
2
4
6
8
10
5' <-----> 3'
Probe Number

Spike-in Hybridization controls intensities and calls

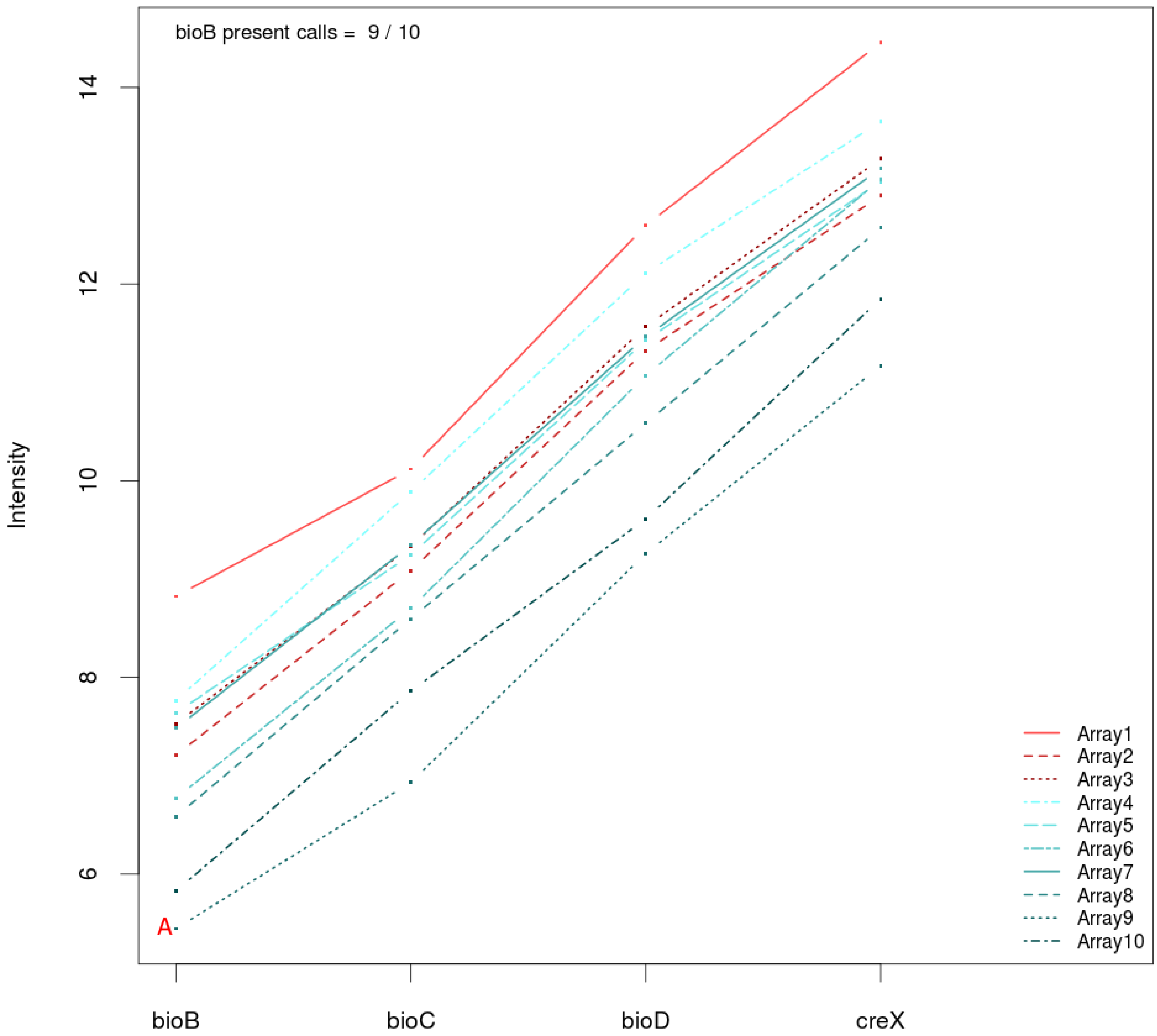

Intensities: OK (bioB < bioC < bioD < creX for all arrays)

BioB present calls: not OK (1 / 10 bioB not called present)

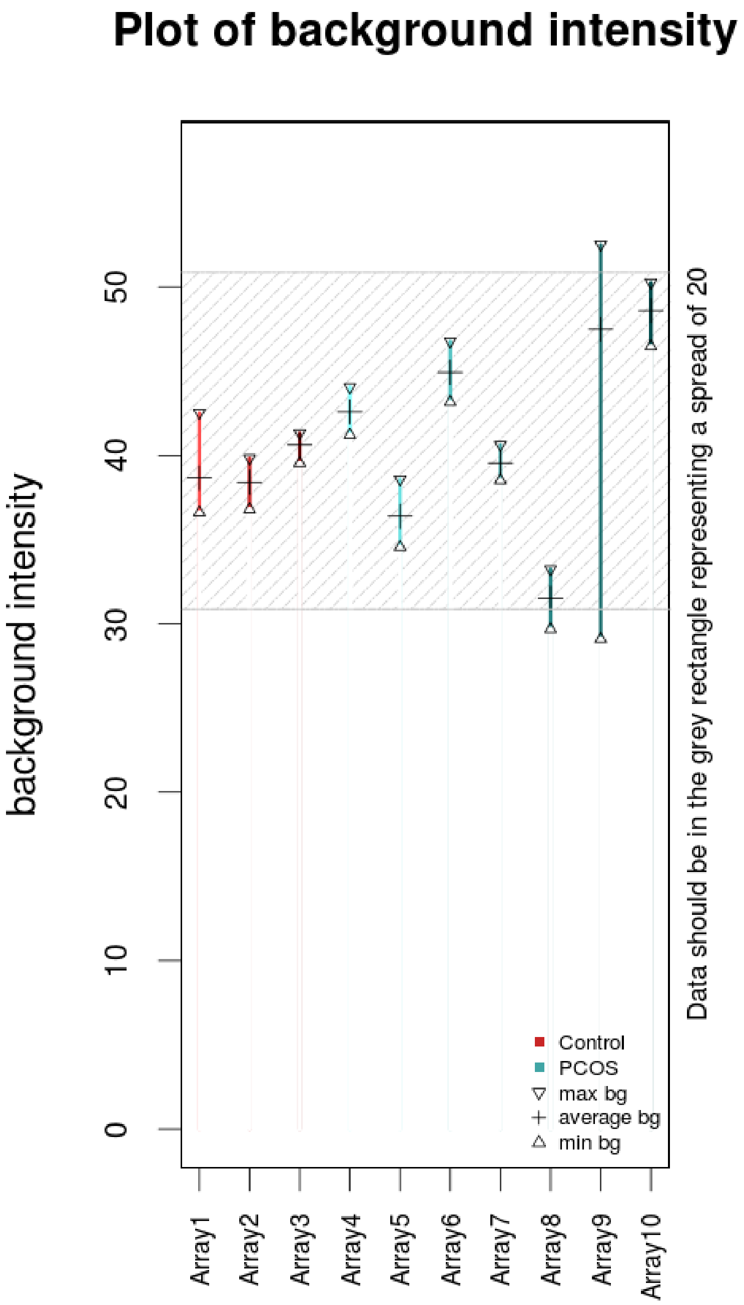

Plot of background intensity
background intensity
0
10
20
30
40
50
Data should be in the grey rectangle representing a spread of 20
Control
PCOS
max bg
average bg
min bg
Array1
Array2
Array3
Array4
Array5
Array6
Array7
Array8
Array9
Array10

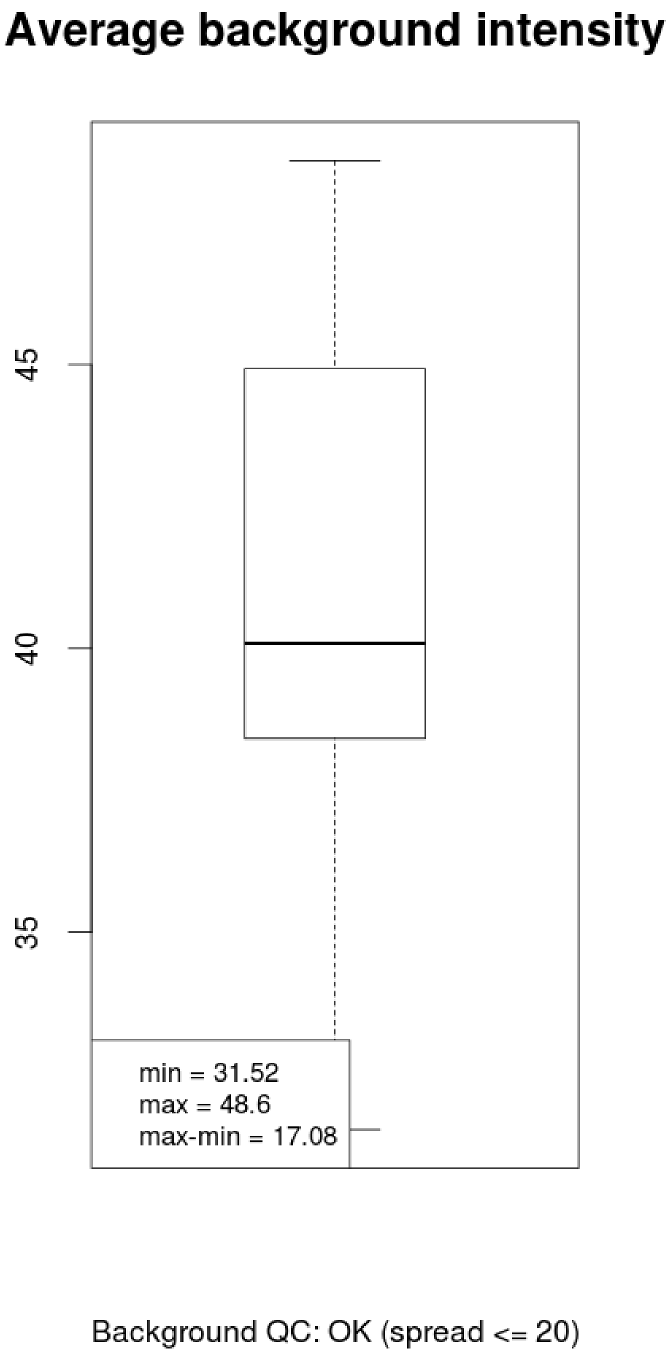

Average background intensity
35
40
45
min = 31.52
max = 48.6
max-min = 17.08
Background QC: OK (spread <= 20)

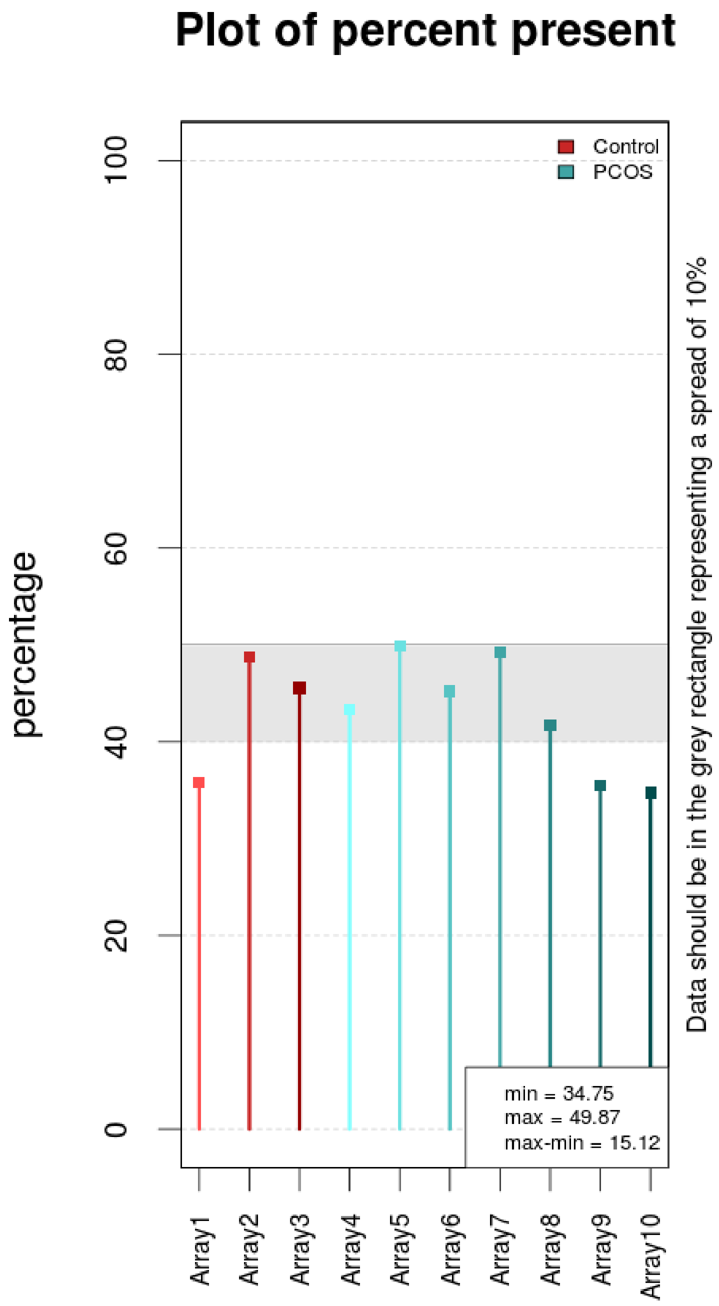

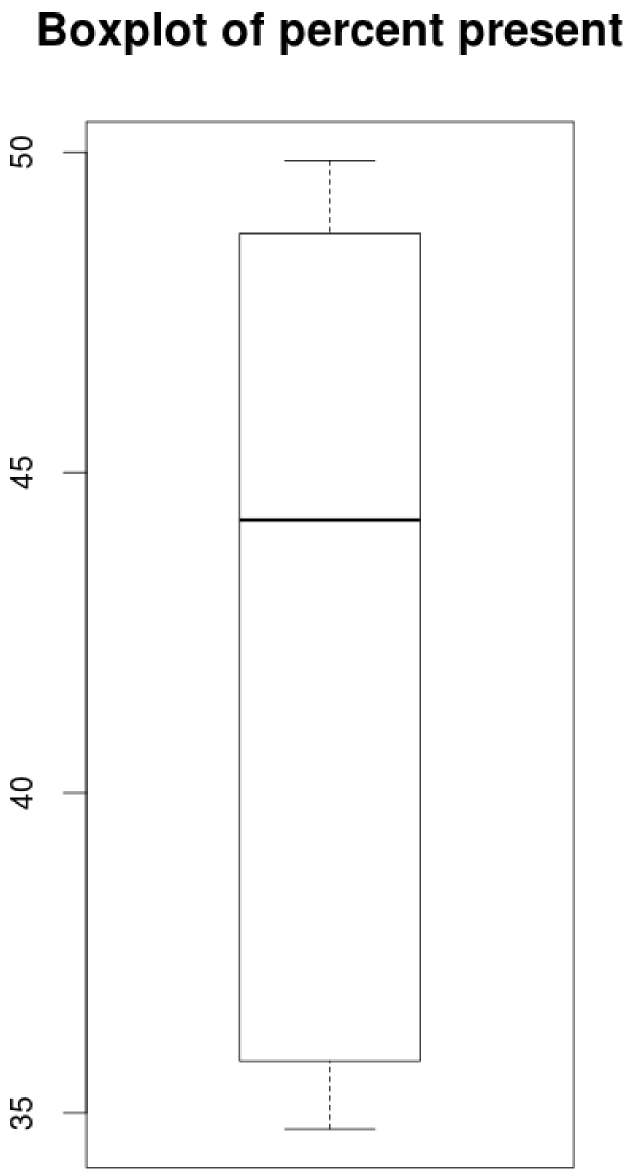

Percent present QC: not OK (spread > 10%)

affx control profiles

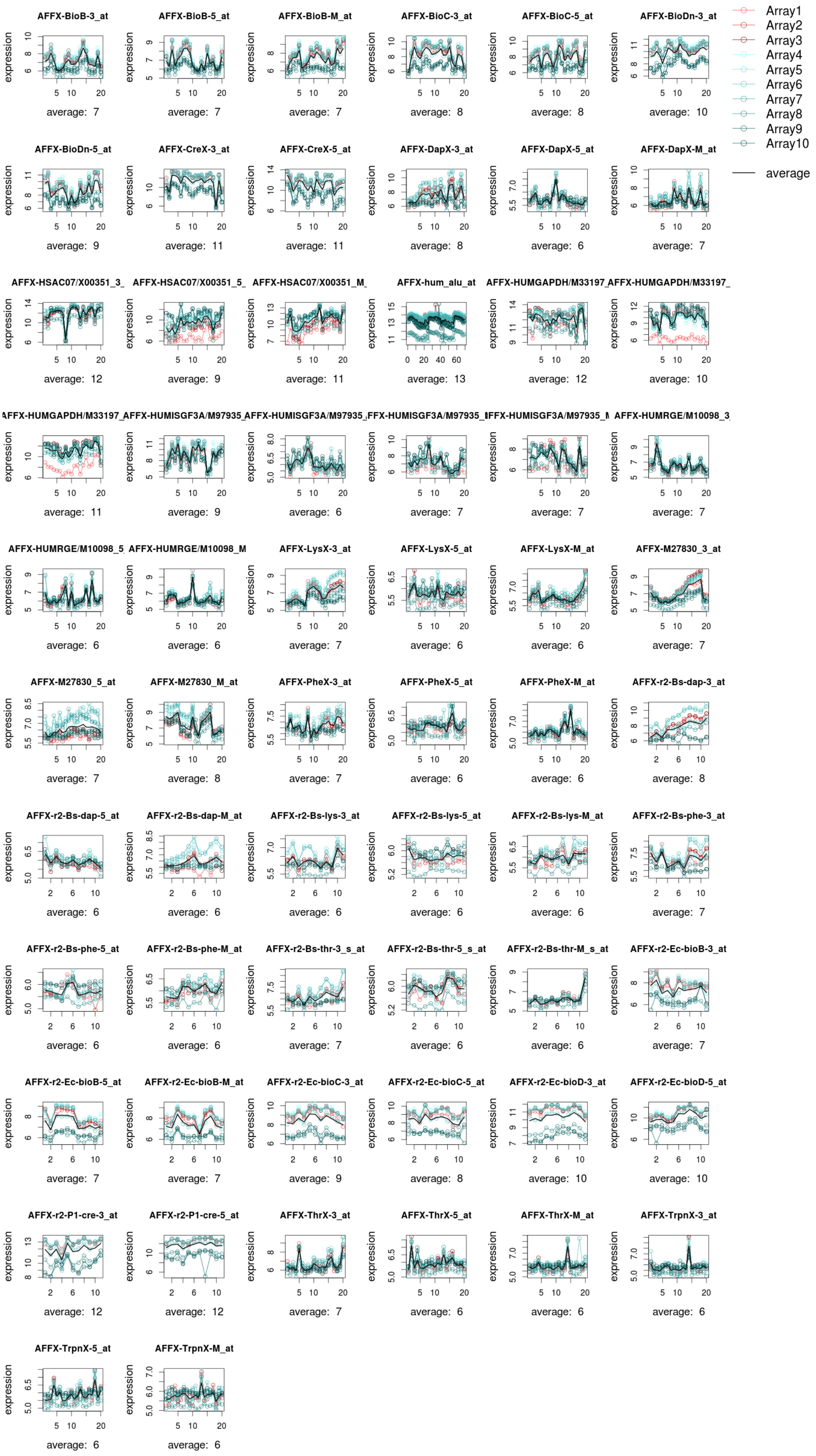

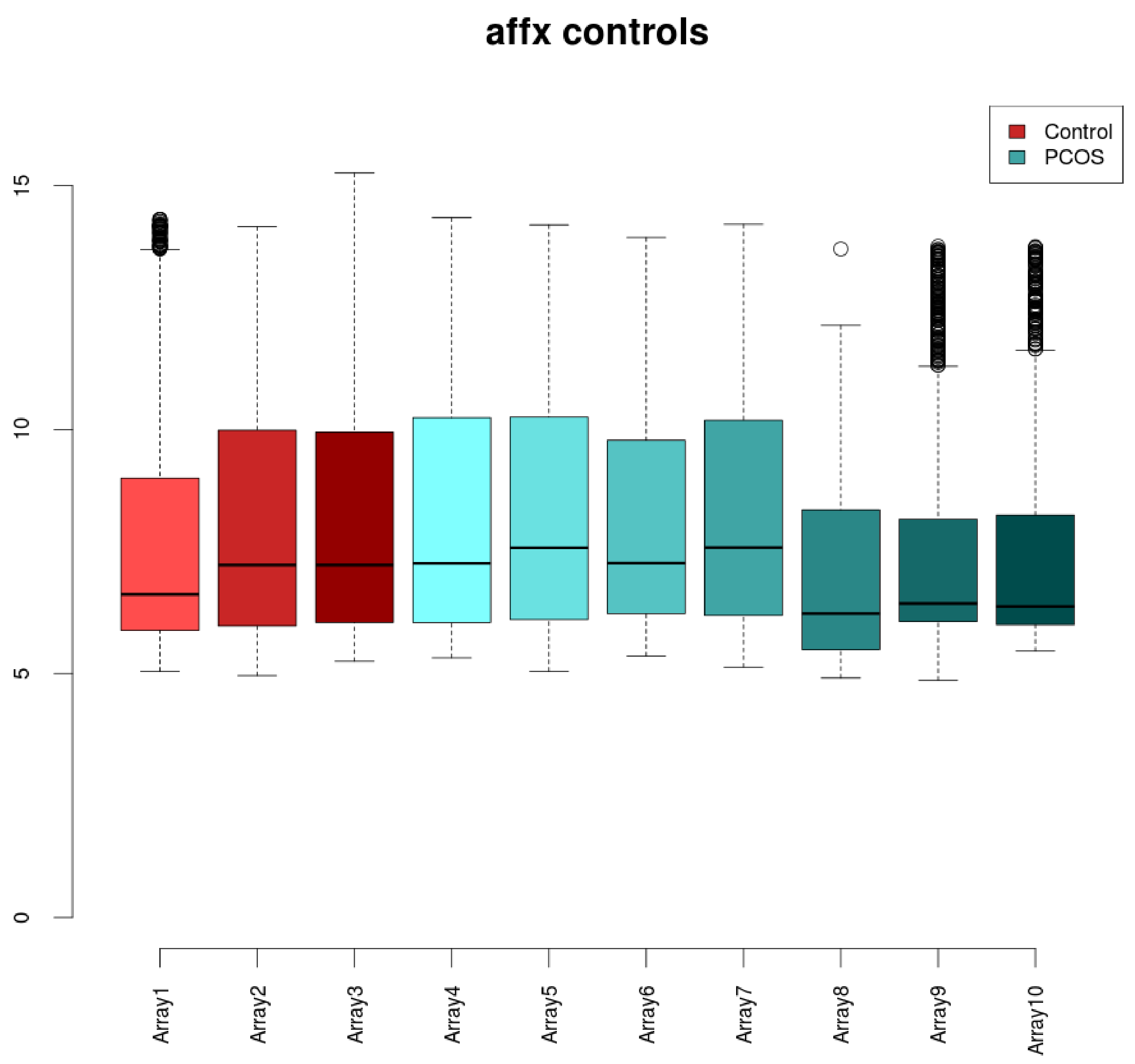
affx controls
Control
PCOS
0
5
10
15
Array1
Array2
Array3
Array4
Array5
Array6
Array7
Array8
Array9
Array10

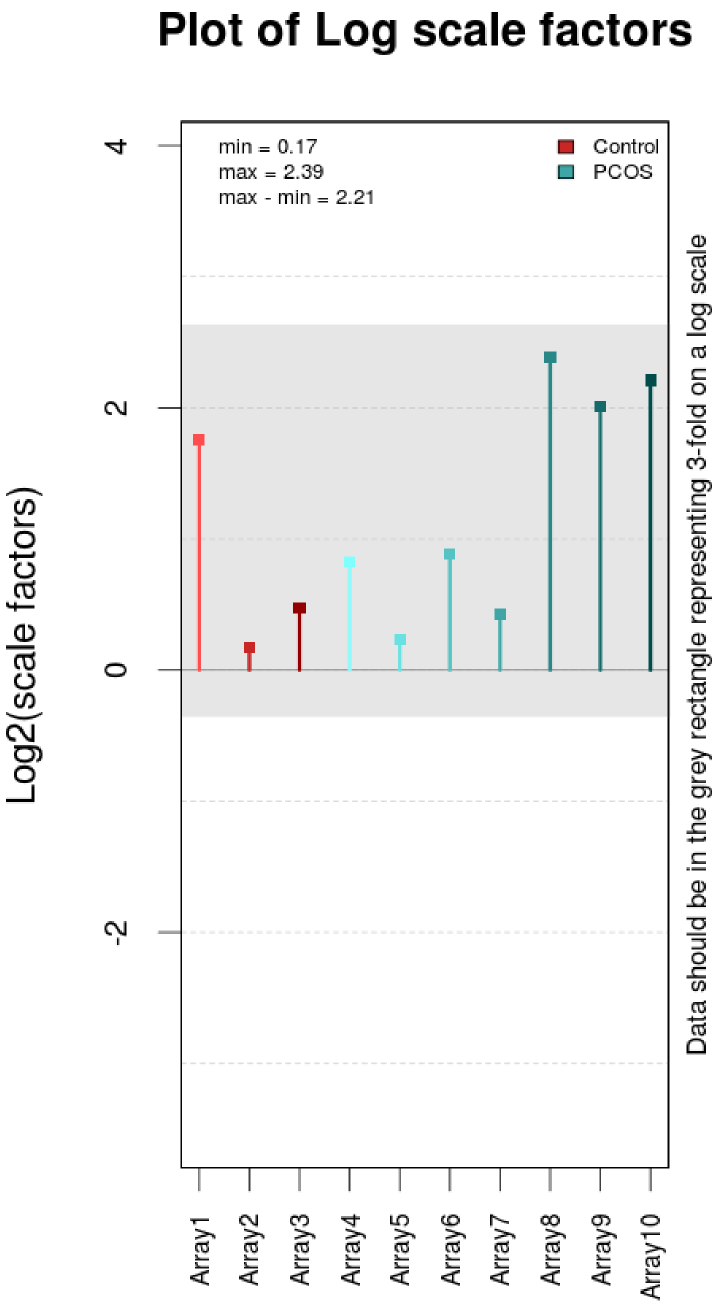
Plot of Log scale factors
min = 0.17
max = 2.39
max - min = 2.21
Control
PCOS
Log2(scale factors)
Data should be in the grey rectangle representing 3-fold on a log scale
4
2
0
-2
Array1
Array2
Array3
Array4
Array5
Array6
Array7
Array8
Array9
Array10

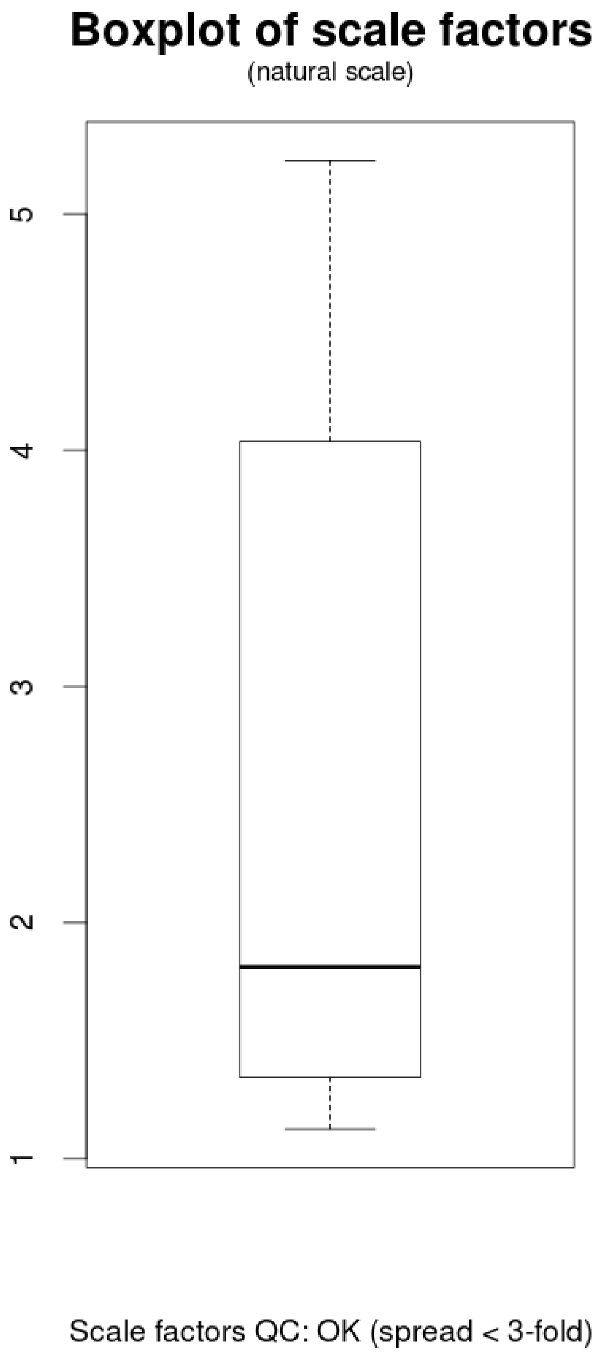
Boxplot of scale factors
(natural scale)
5
4
3
2
1
Scale factors QC: OK (spread < 3-fold)

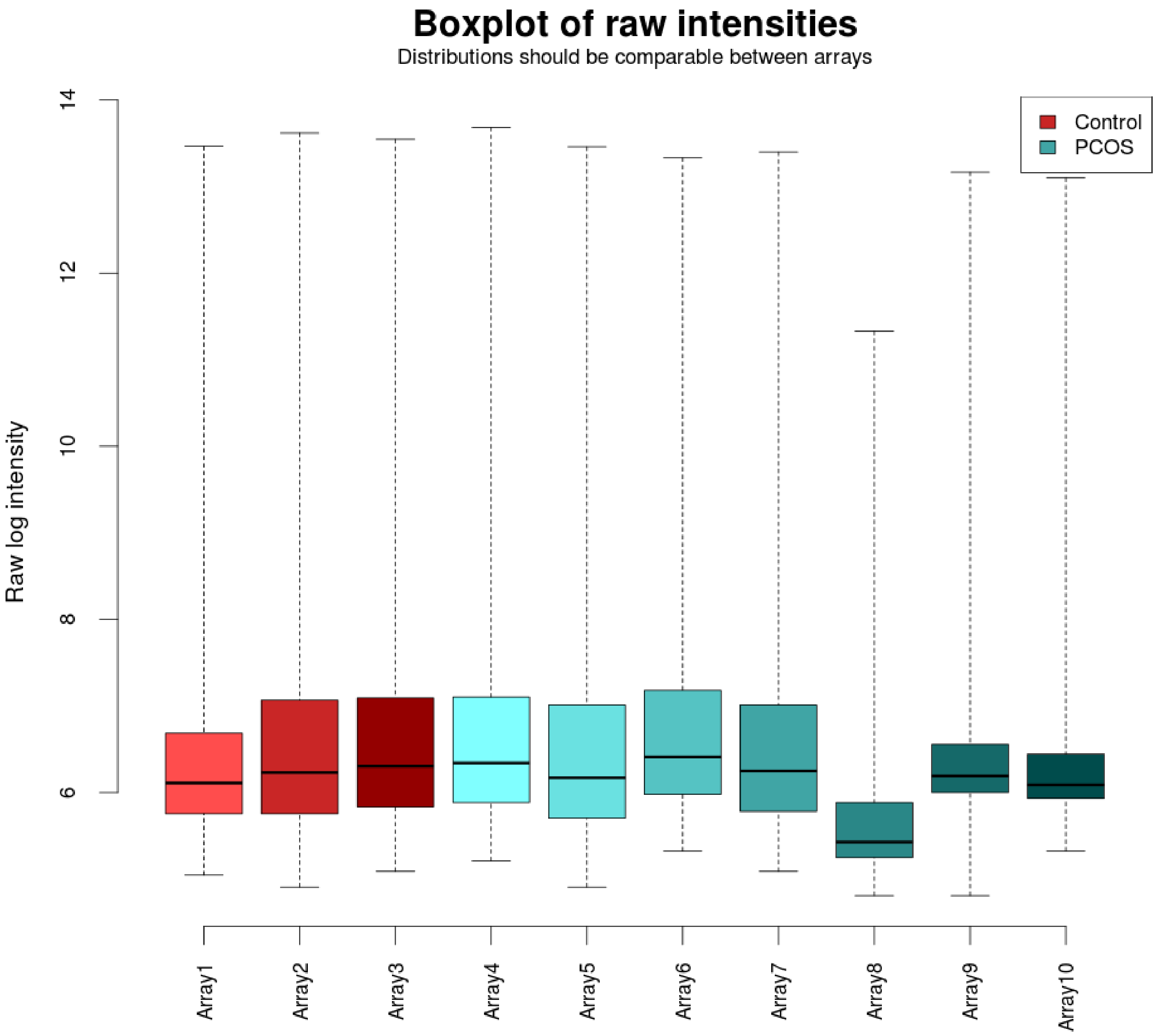

Boxplot of raw intensities
Distributions should be comparable between arrays
Raw log intensity
14
12
10
8
6
Control
PCOS
Array1
Array2
Array3
Array4
Array5
Array6
Array7
Array8
Array9
Array10

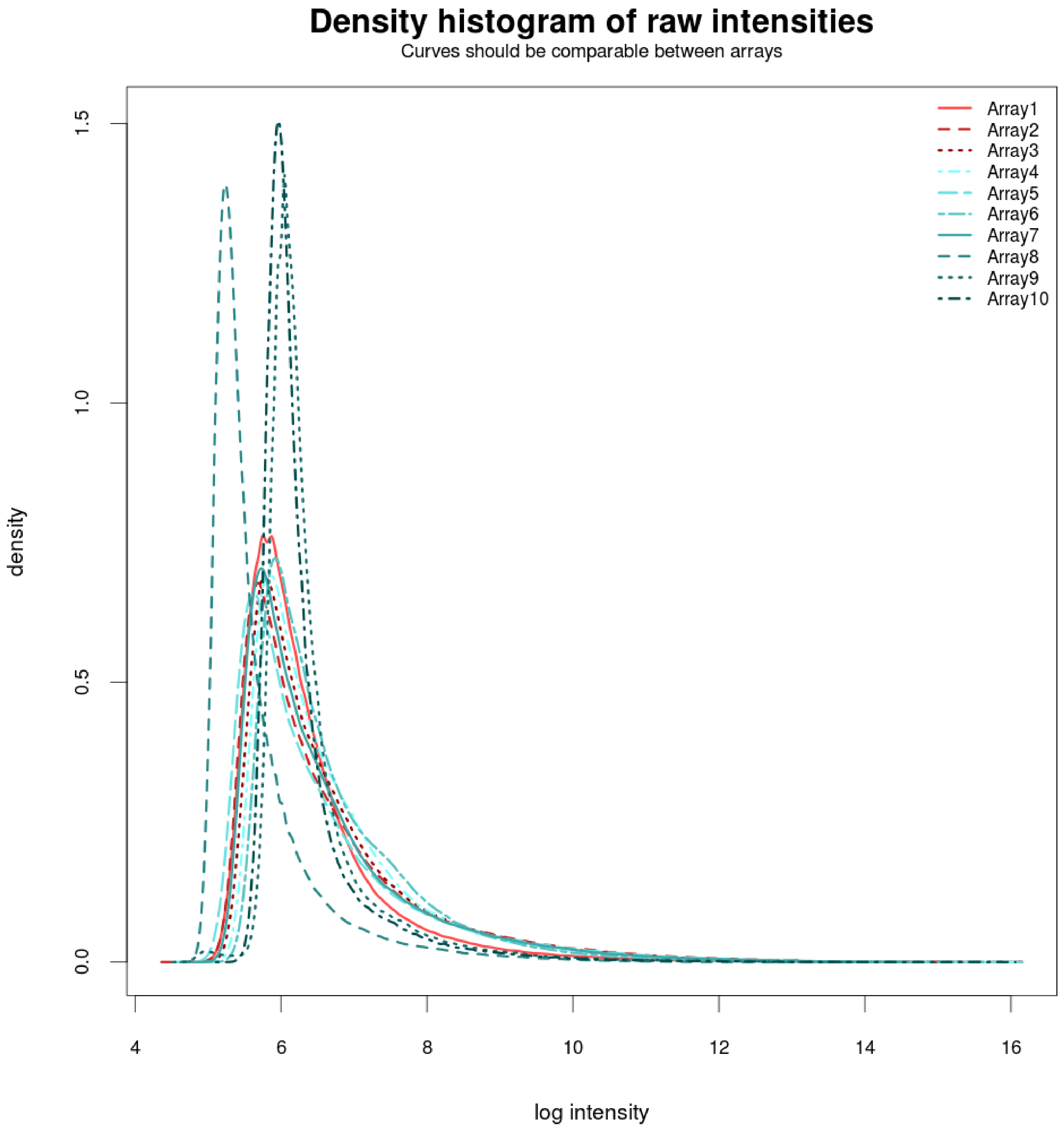
Density histogram of raw intensities
Curves should be comparable between arrays
density
log intensity
0.0
0.5
1.0
1.5
4
6
8
10
12
14
16
Array1
Array2
Array3
Array4
Array5
Array6
Array7
Array8
Array9
Array10

MA plots of raw data 1 / 2

Array1 vs pseudo-median reference chip

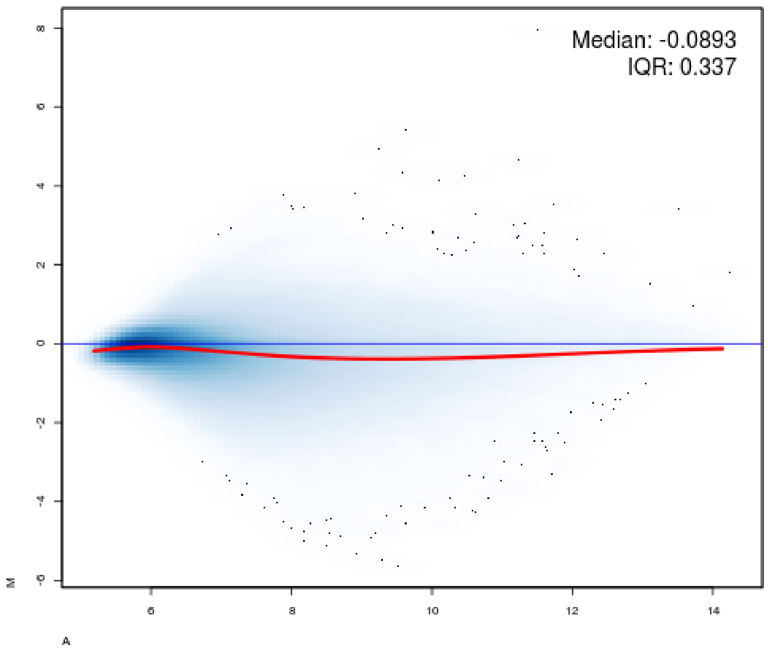

Array2 vs pseudo-median reference chip

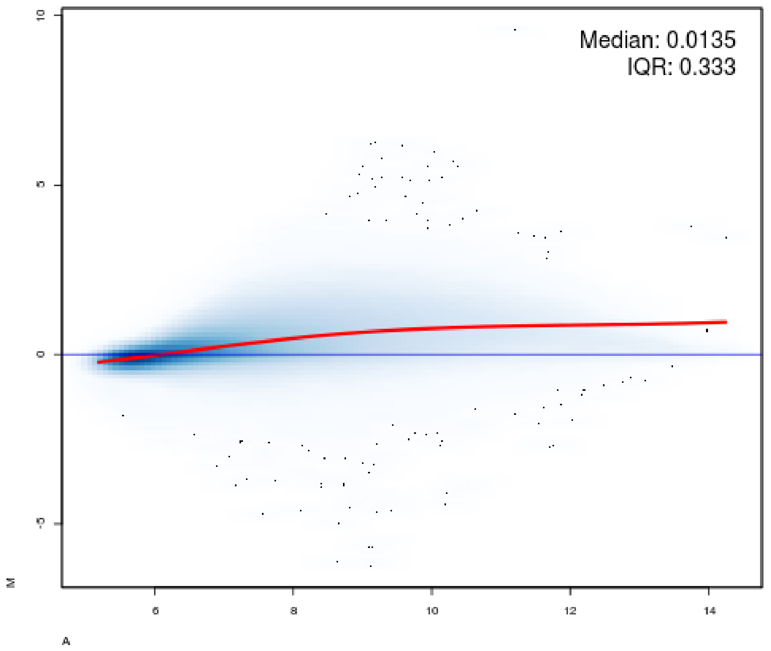

Array3 vs pseudo-median reference chip

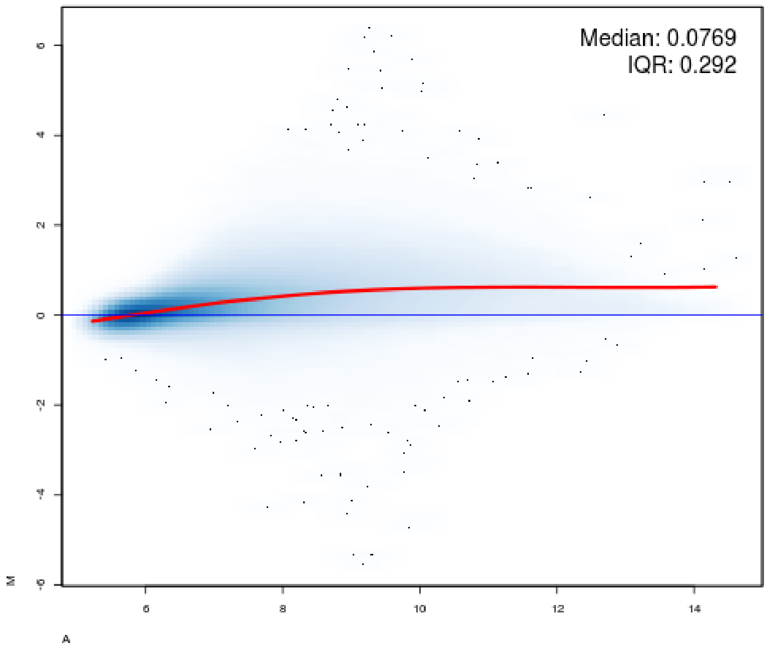

Array4 vs pseudo-median reference chip

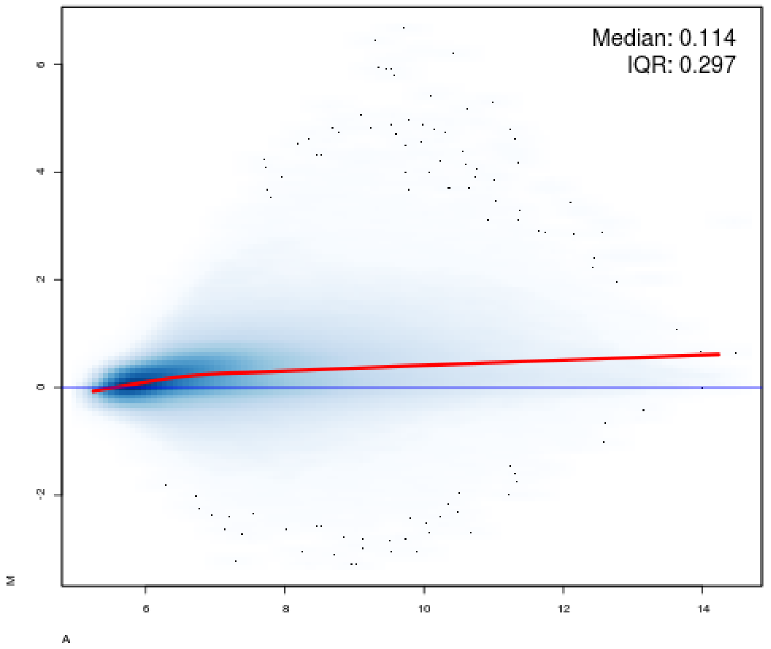

Array5 vs pseudo-median reference chip

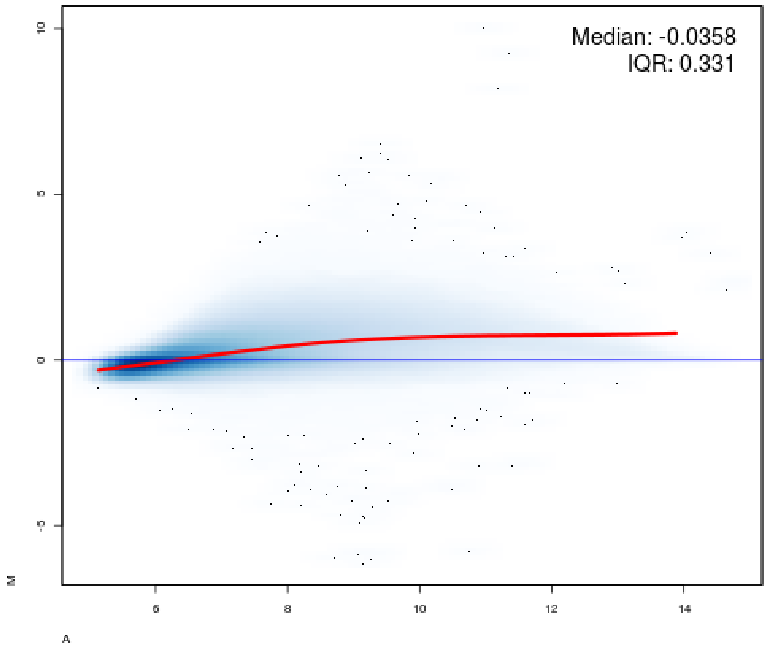

Array6 vs pseudo-median reference chip

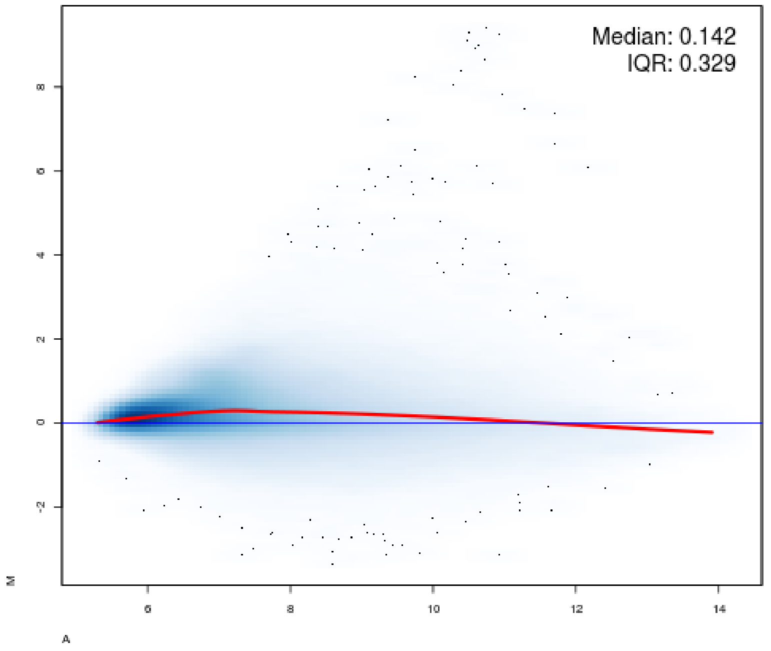

MA plots of raw data 2 / 2

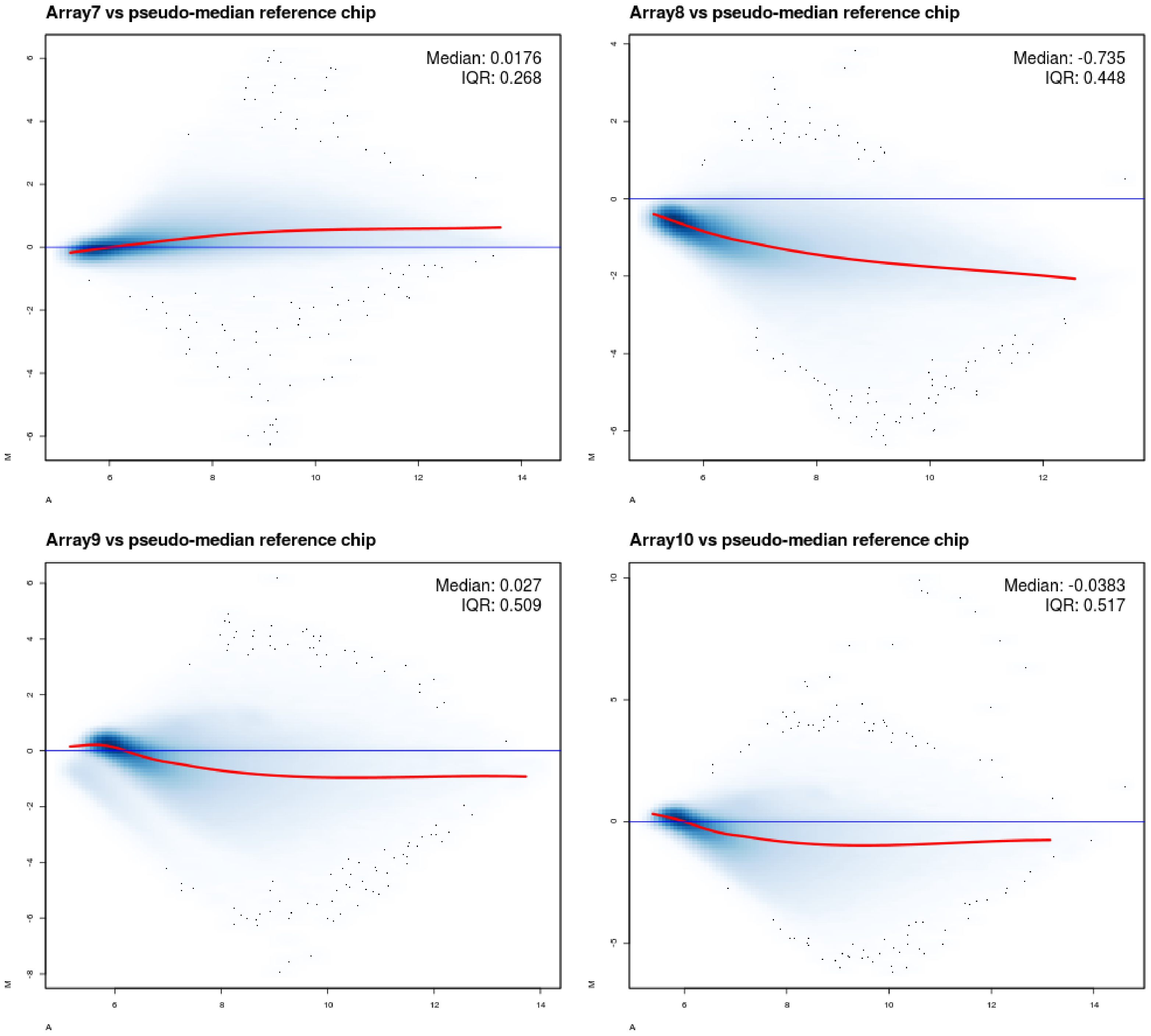

2D virtual PLM image for model characteristic: resids 1 / 2

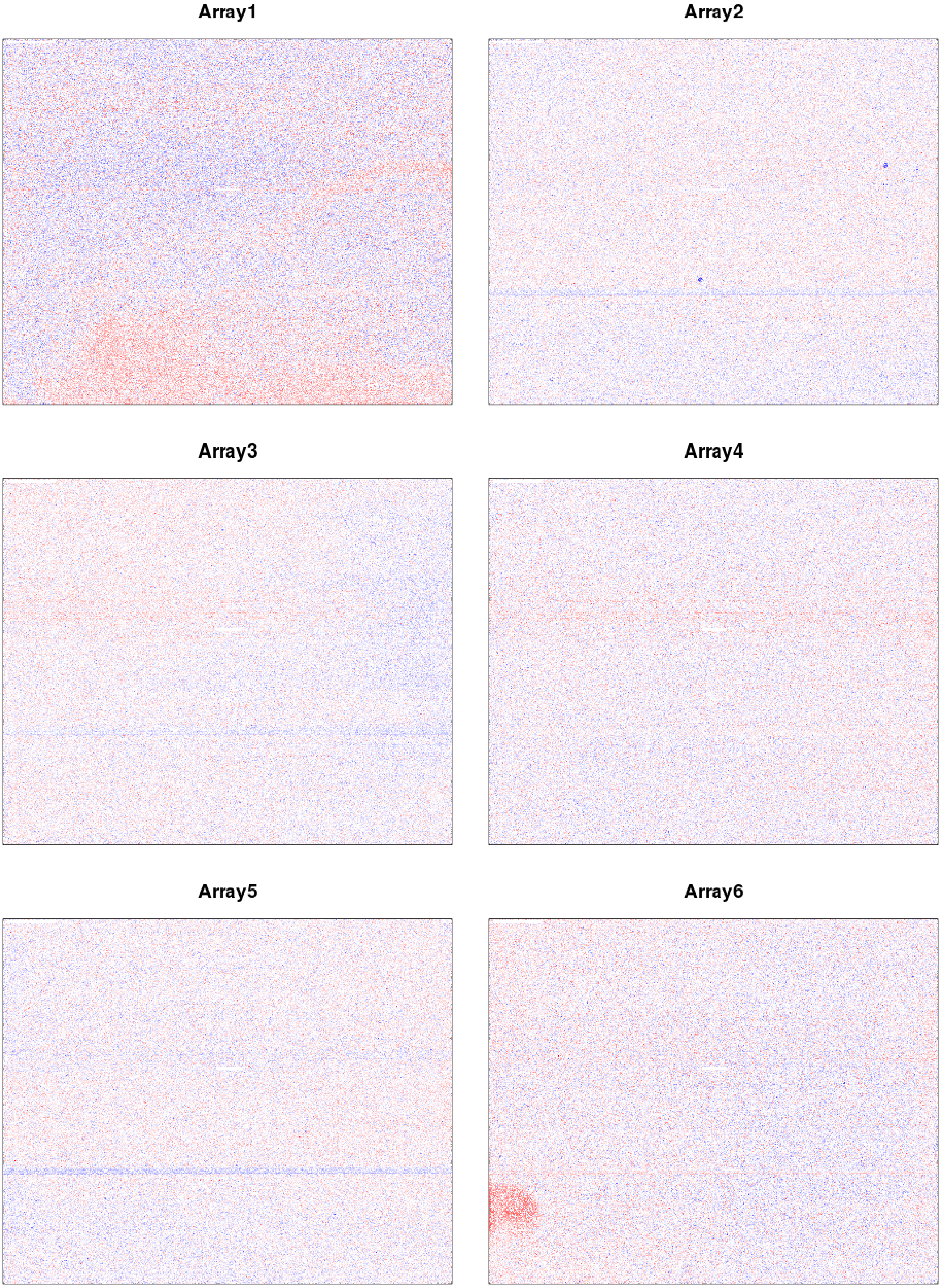

2D virtual PLM image for model characteristic: resids 2 / 2

Array7

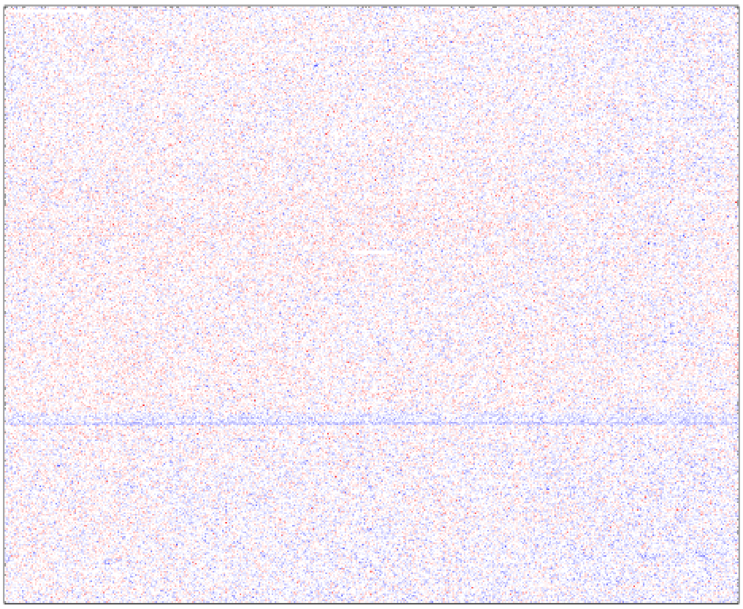

Array8

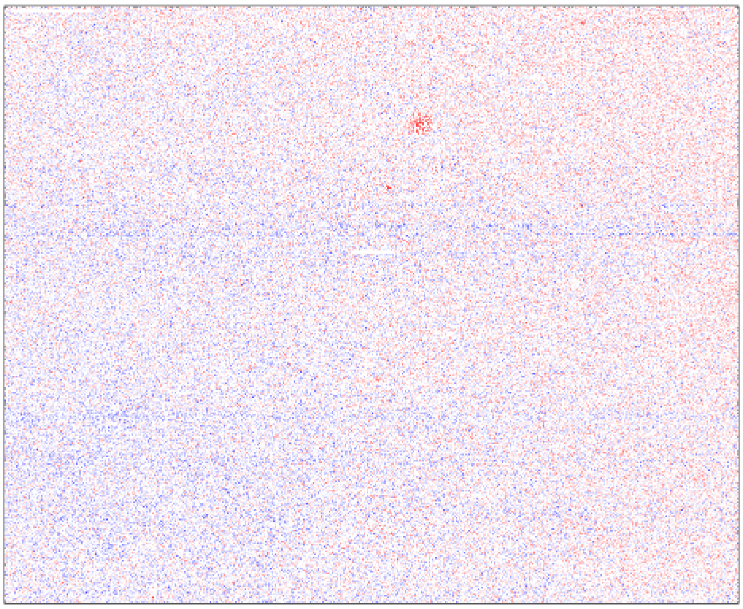

Array9

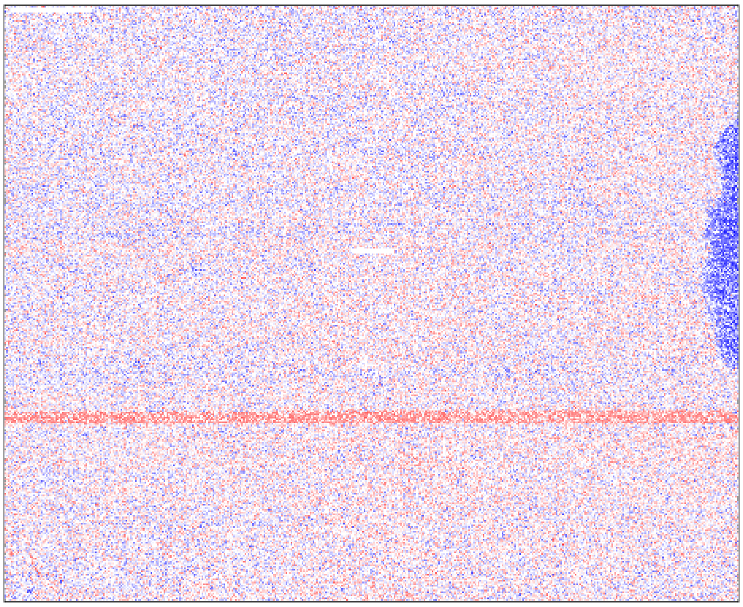

Array10

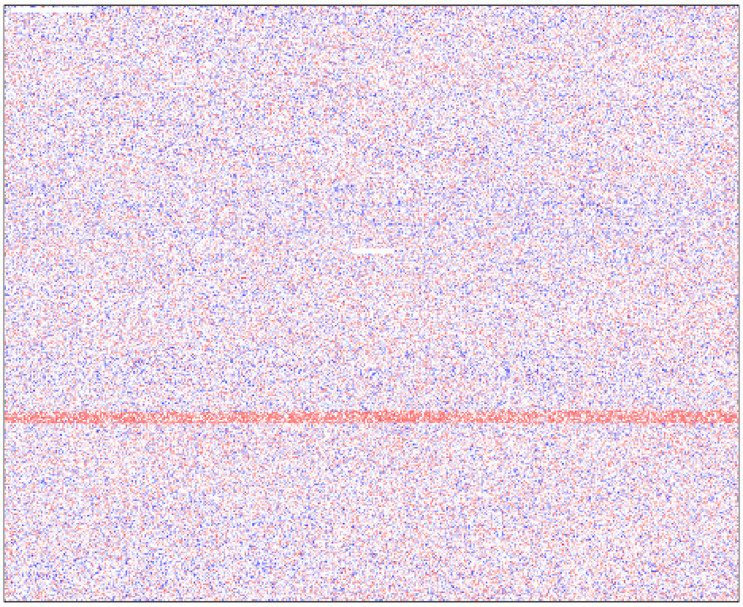

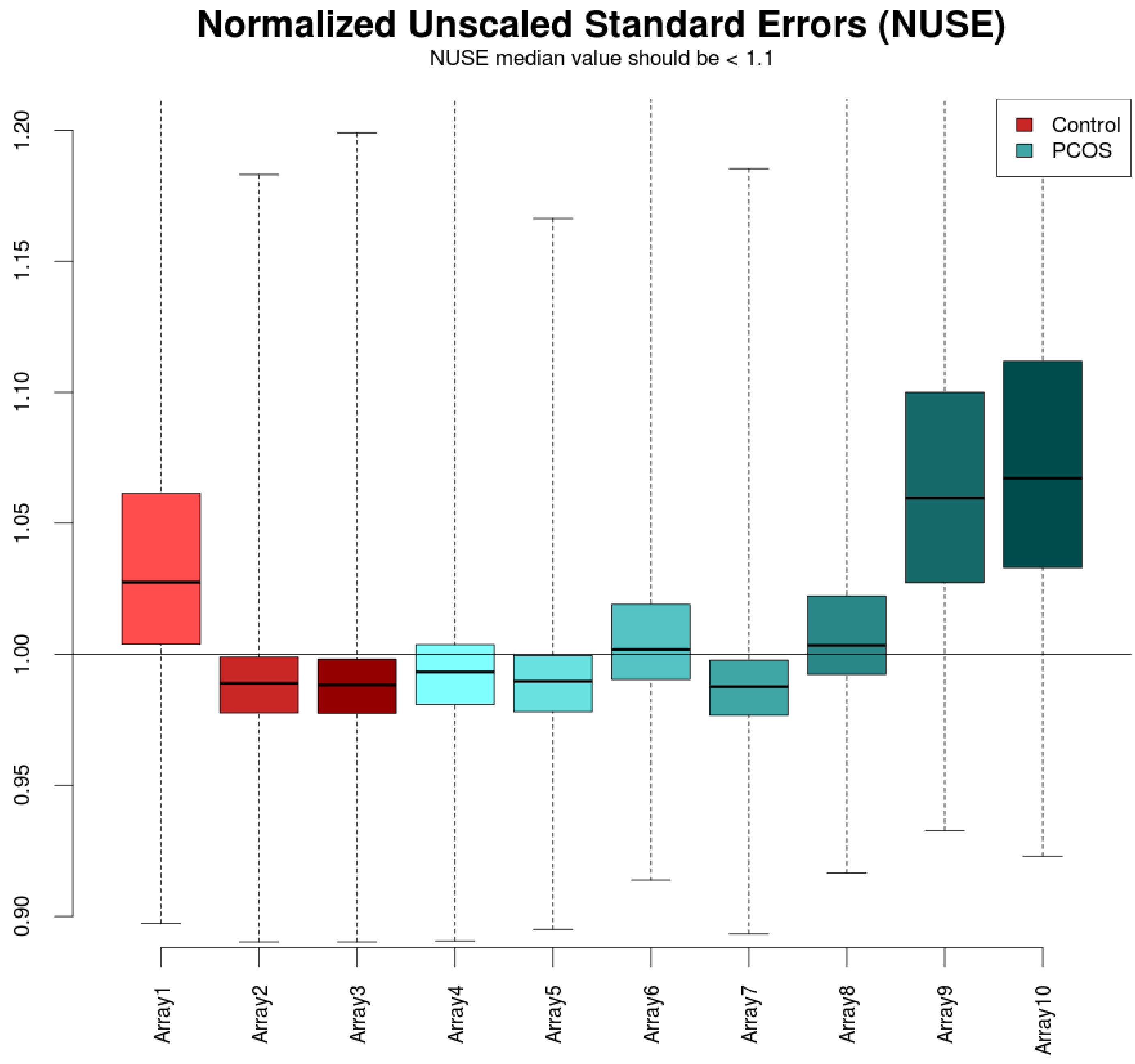
Normalized Unscaled Standard Errors (NUSE)
NUSE median value should be < 1.1
Control
PCOS
1.20
1.15
1.10
1.05
1.00
0.95
0.90
Array1
Array2
Array3
Array4
Array5
Array6
Array7
Array8
Array9
Array10

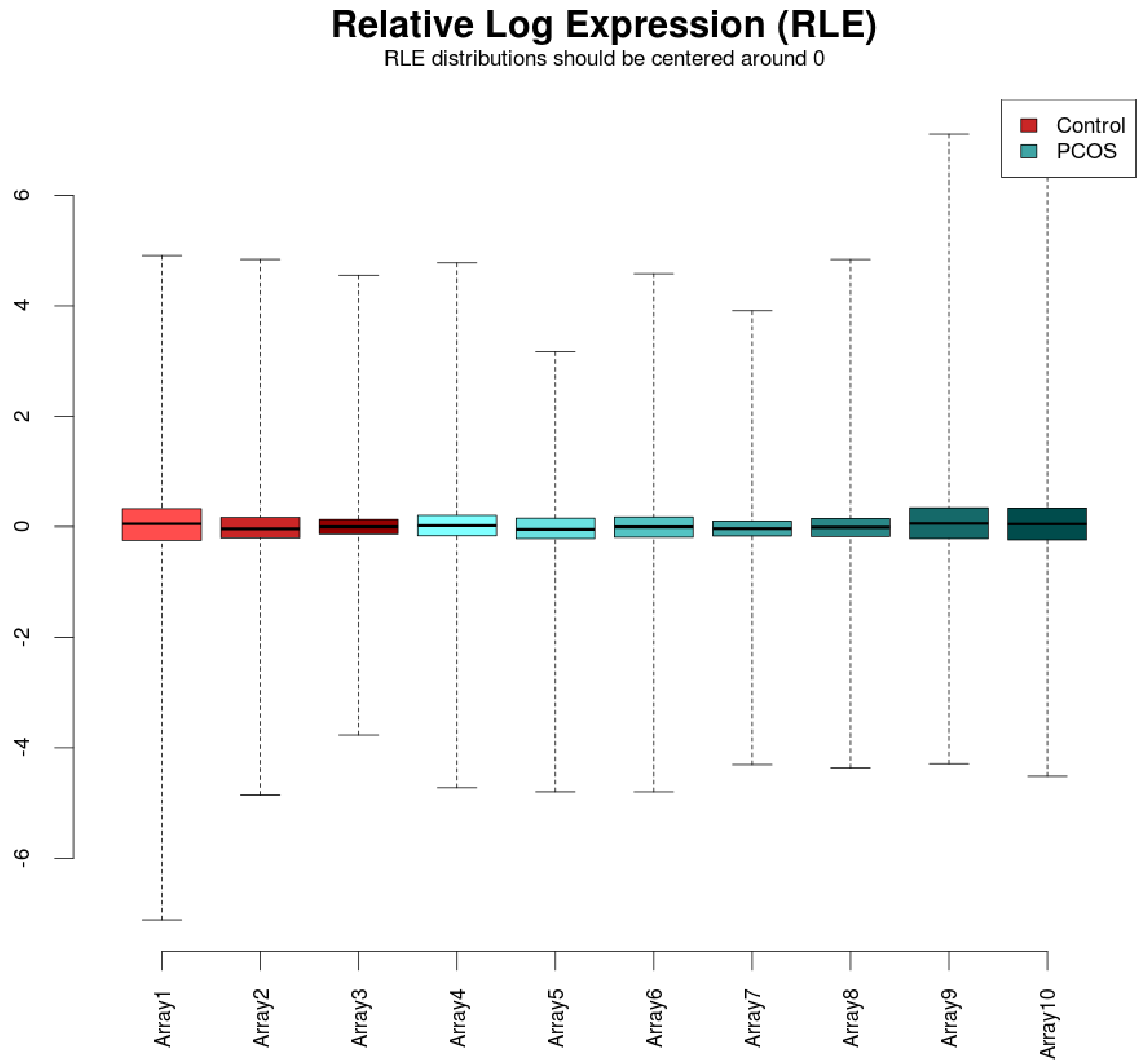

Relative Log Expression (RLE)
RLE distributions should be centered around 0
Control
PCOS
6
4
2
0
-2
-4
-6
Array1
Array2
Array3
Array4
Array5
Array6
Array7
Array8
Array9
Array10

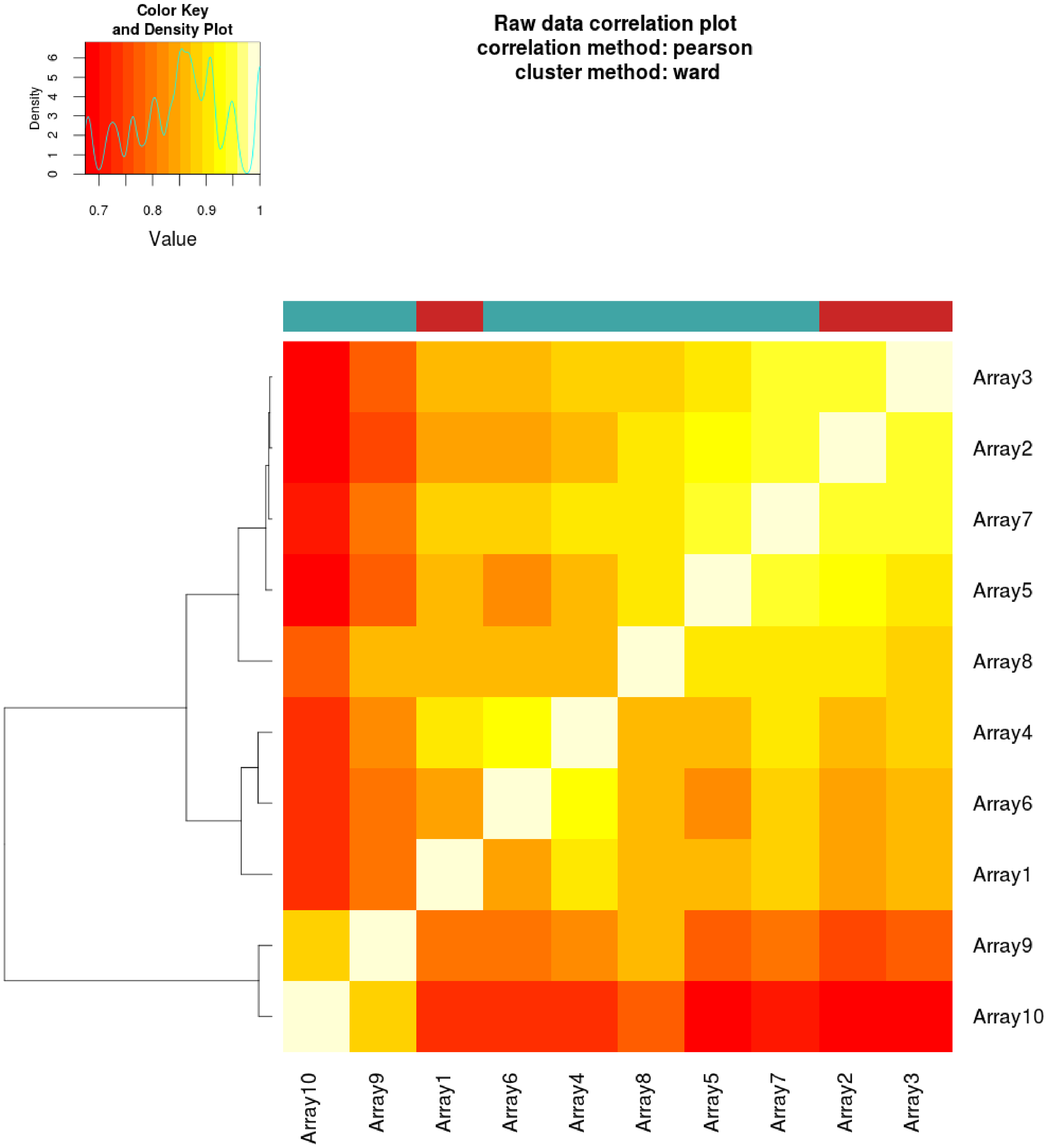

Color Key
and Density Plot
Density
0 1 2 3 4 5 6
0.7 0.8 0.9 1
Value
Raw data correlation plot
correlation method: pearson
cluster method: ward
Array3
Array2
Array7
Array5
Array8
Array4
Array6
Array1
Array9
Array10
Array10
Array9
Array1
Array6
Array4
Array8
Array5
Array7
Array2
Array3

PCA analysis of Raw data

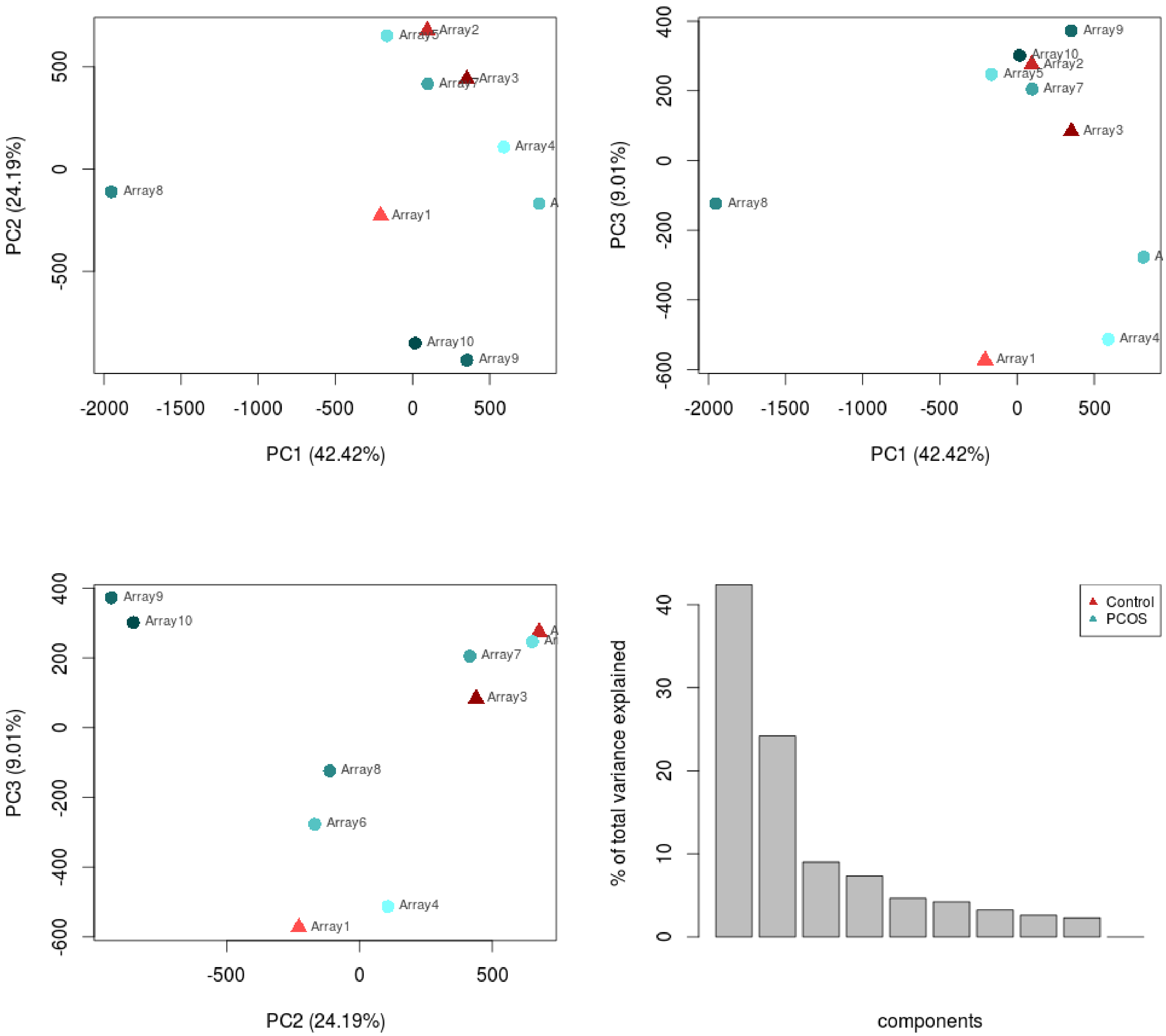

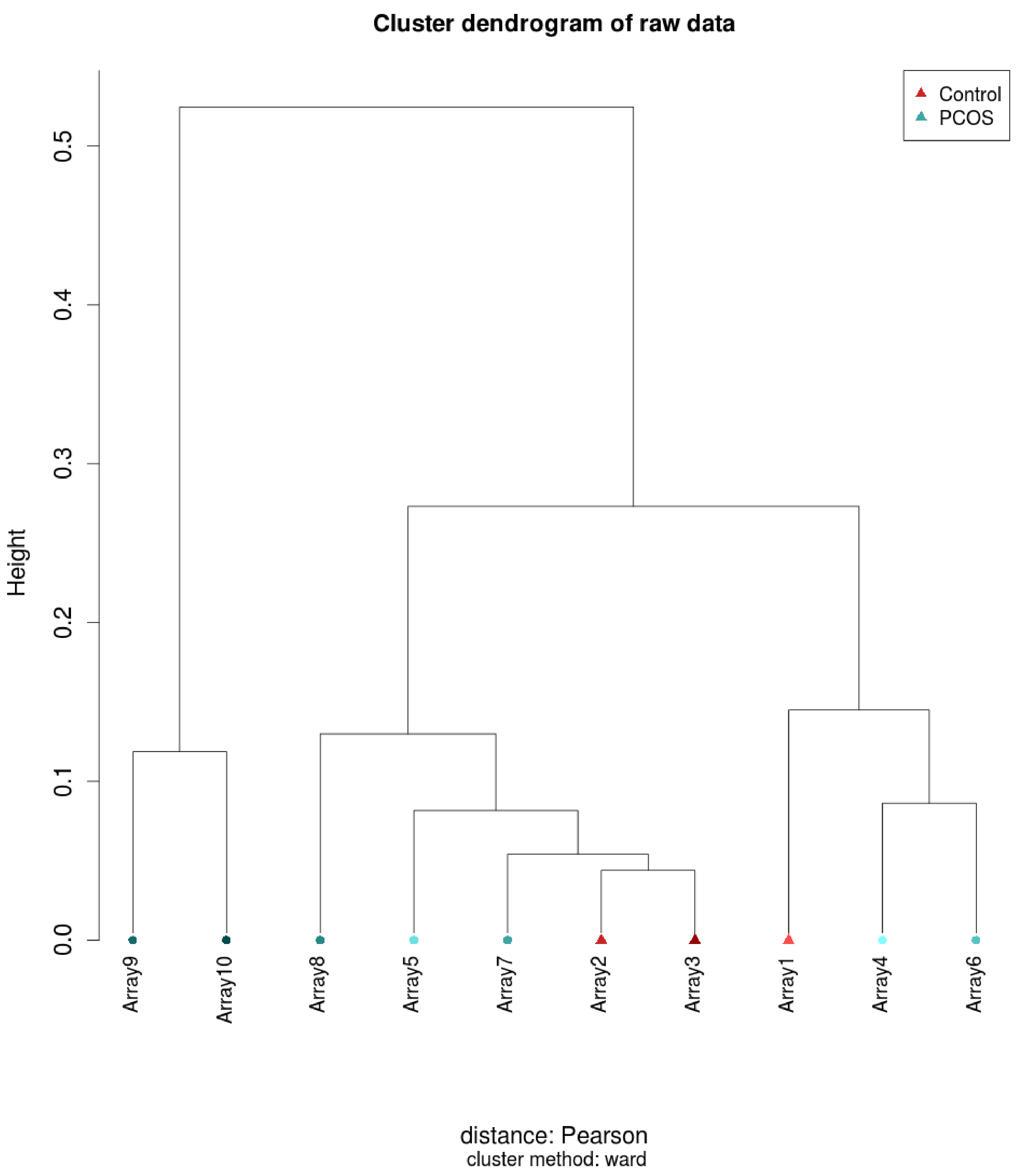

Cluster dendrogram of raw data
Control
PCOS
Height
0.0
0.1
0.2
0.3
0.4
0.5
Array9
Array10
Array8
Array5
Array7
Array2
Array3
Array1
Array4
Array6
distance: Pearson
cluster method: ward

Pre-processing of Raw Data

Method: GCRMA
Annotation: hgu133plus2_Hs_ENSG

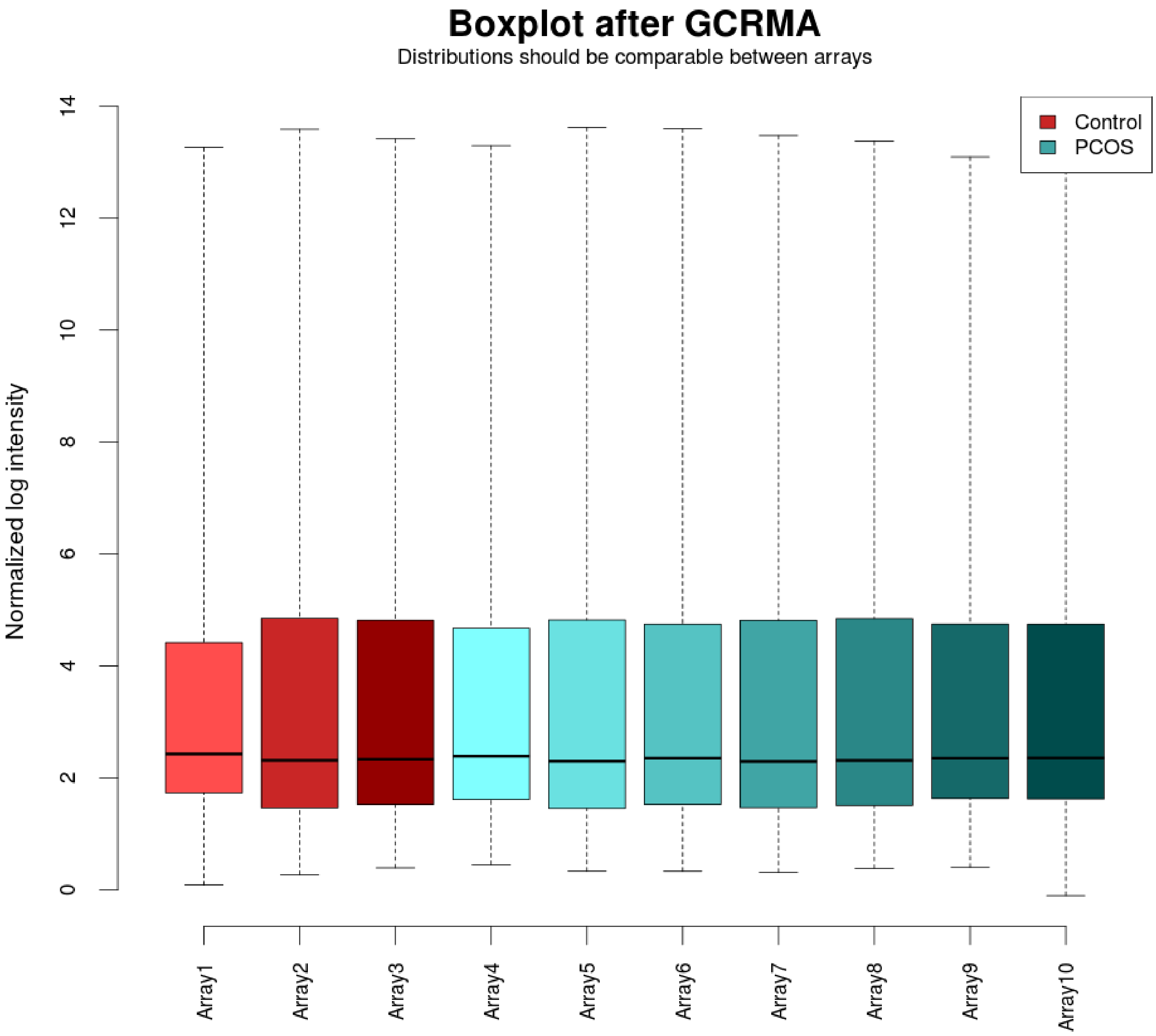
Boxplot after GCRMA
Distributions should be comparable between arrays
Normalized log intensity
0
2
4
6
8
10
12
14
Control
PCOS
Array1
Array2
Array3
Array4
Array5
Array6
Array7
Array8
Array9
Array10

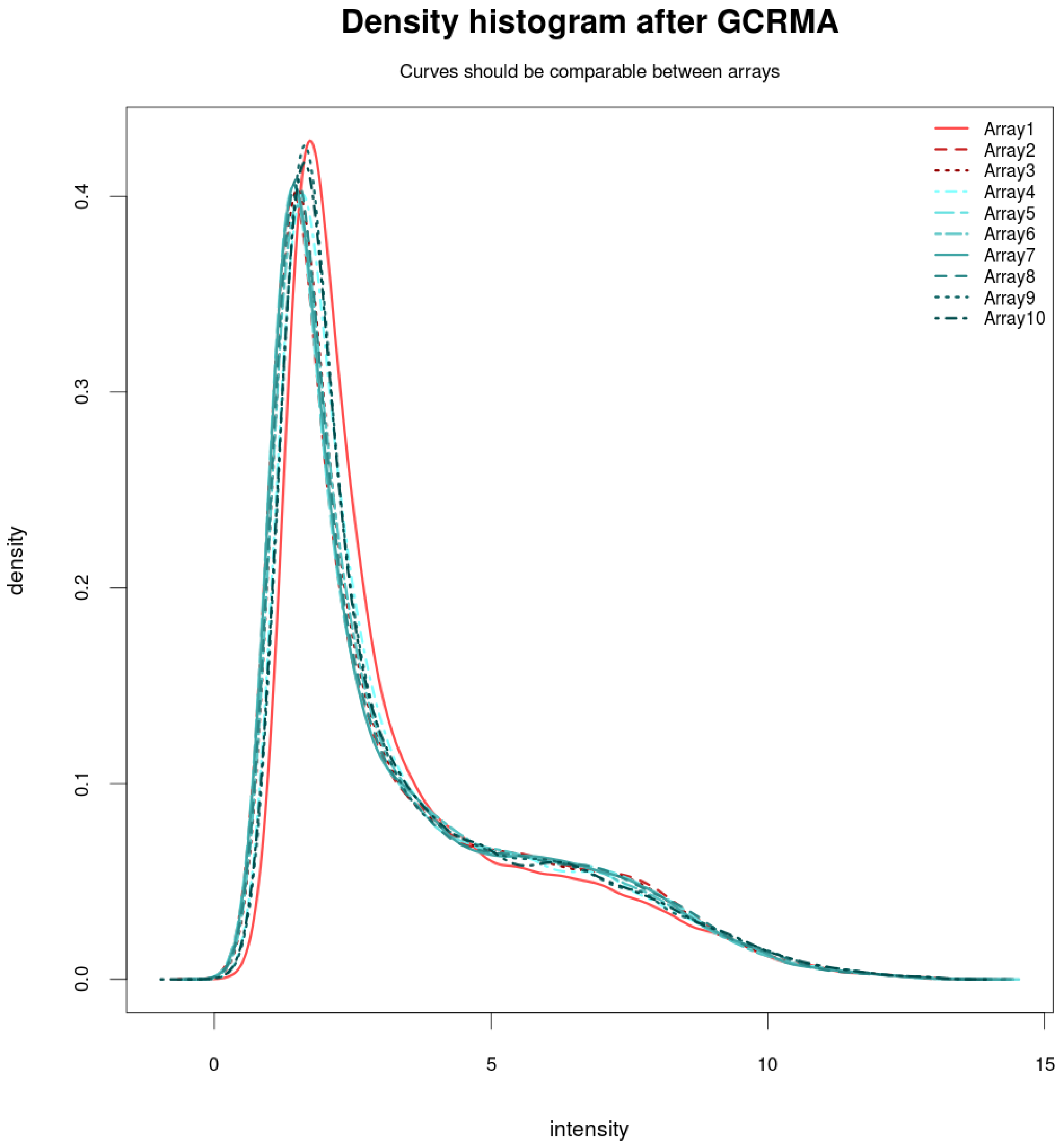

Density histogram after GCRMA
Curves should be comparable between arrays
density
intensity
0.0
0.1
0.2
0.3
0.4
0
5
10
15
Array1
Array2
Array3
Array4
Array5
Array6
Array7
Array8
Array9
Array10

MA plots afterGCRMAnormalization 1 / 2

Array1 vs pseudo-median reference chip

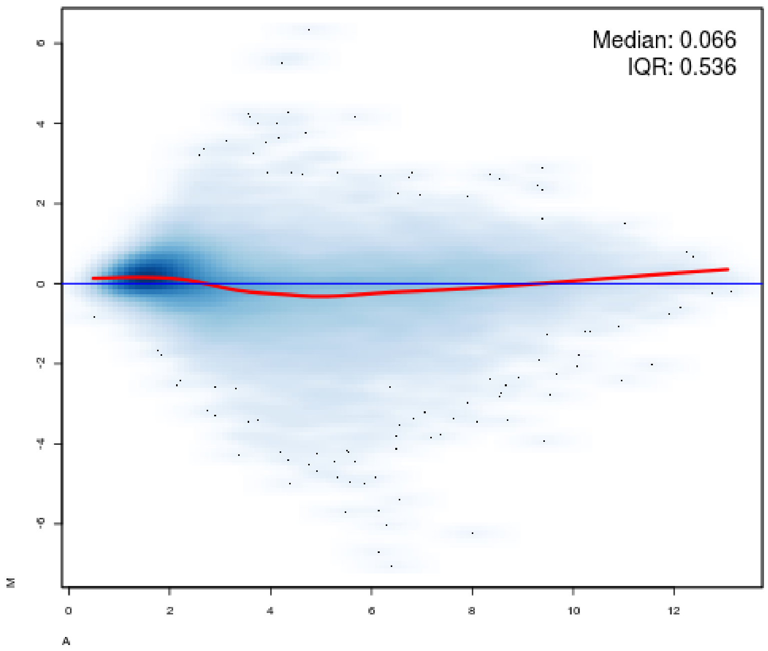

Array2 vs pseudo-median reference chip

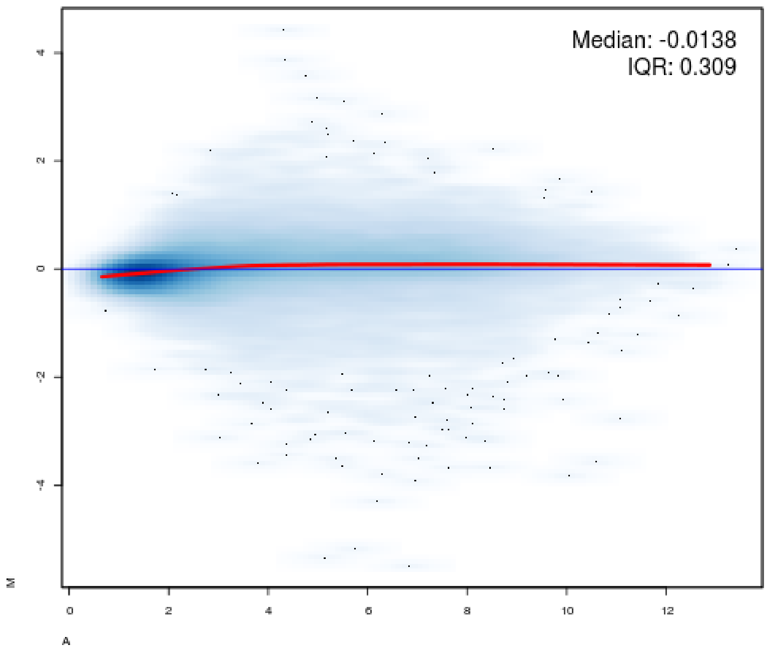

Array3 vs pseudo-median reference chip

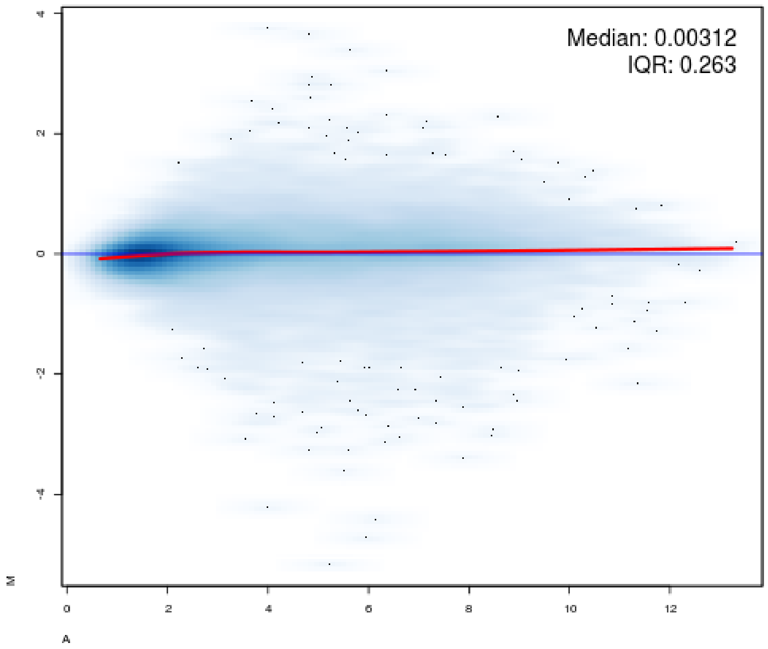

Array4 vs pseudo-median reference chip

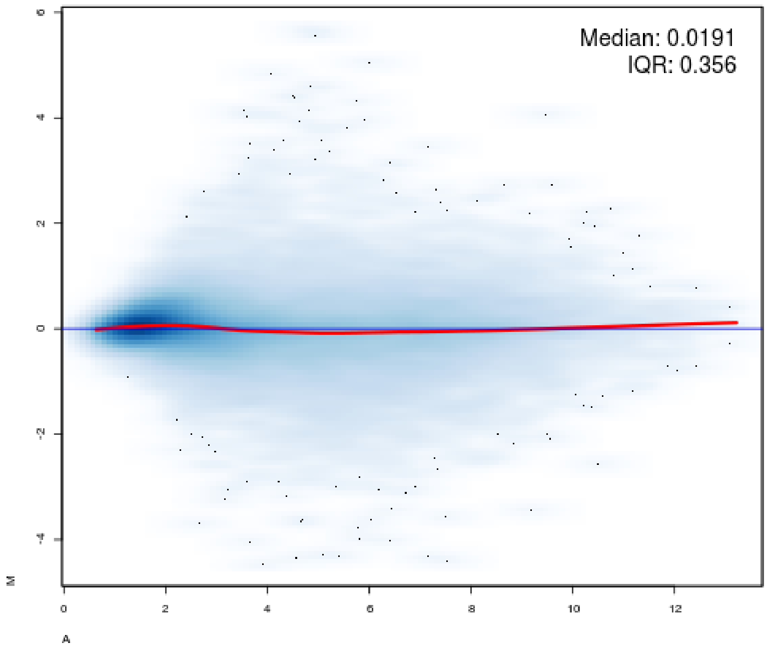

Array5 vs pseudo-median reference chip

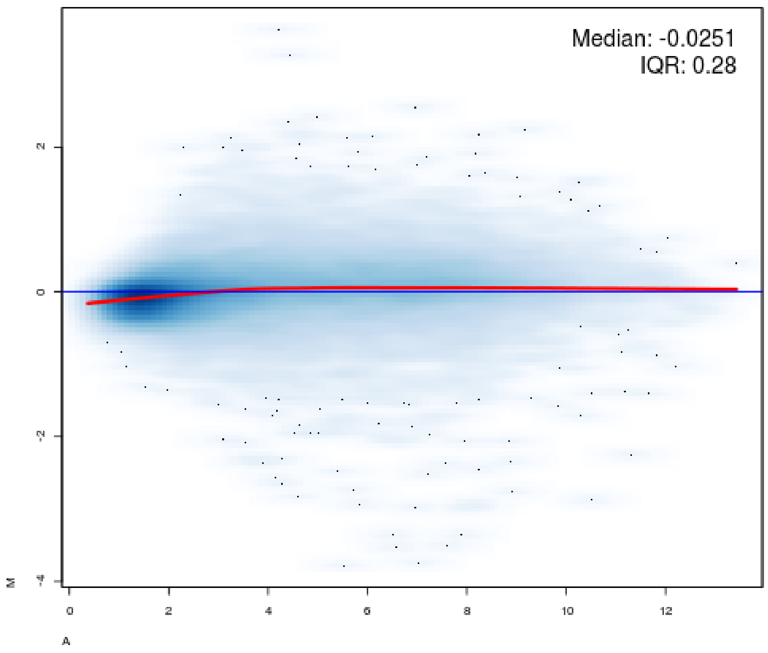

Array6 vs pseudo-median reference chip

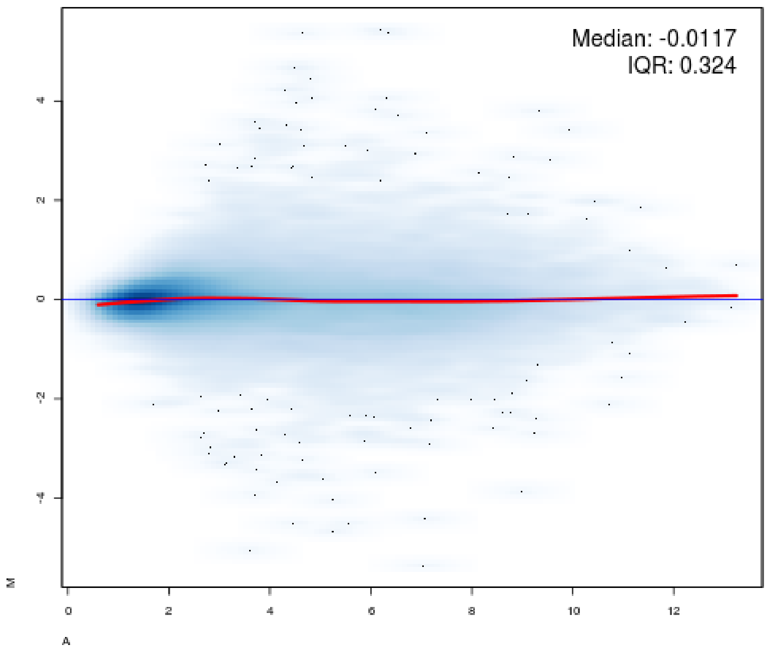

MA plots afterGCRMAnormalization 2 / 2

Array7 vs pseudo-median reference chip

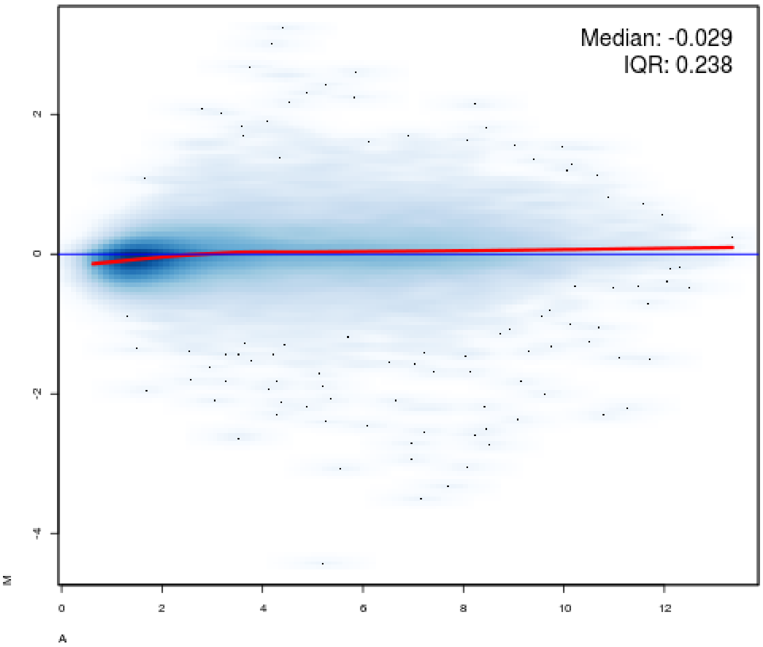

Array8 vs pseudo-median reference chip

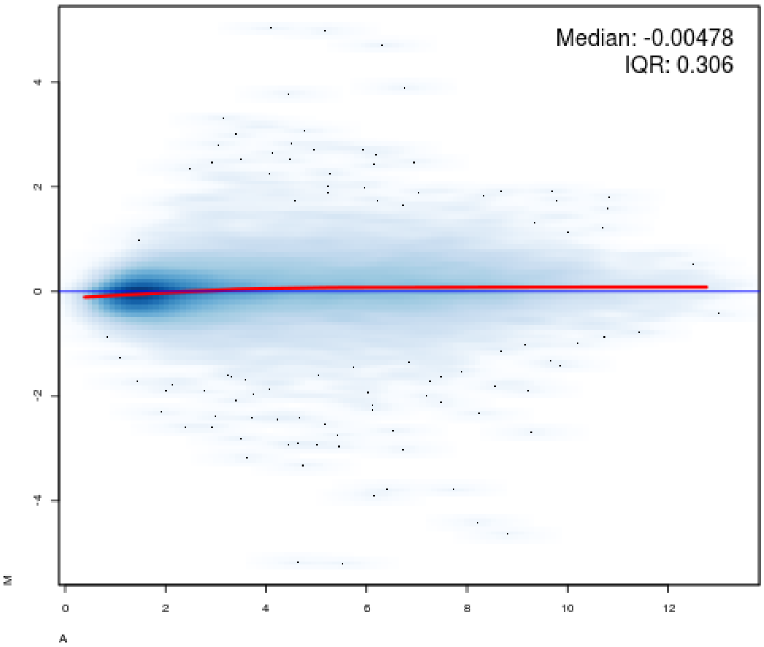

Array9 vs pseudo-median reference chip

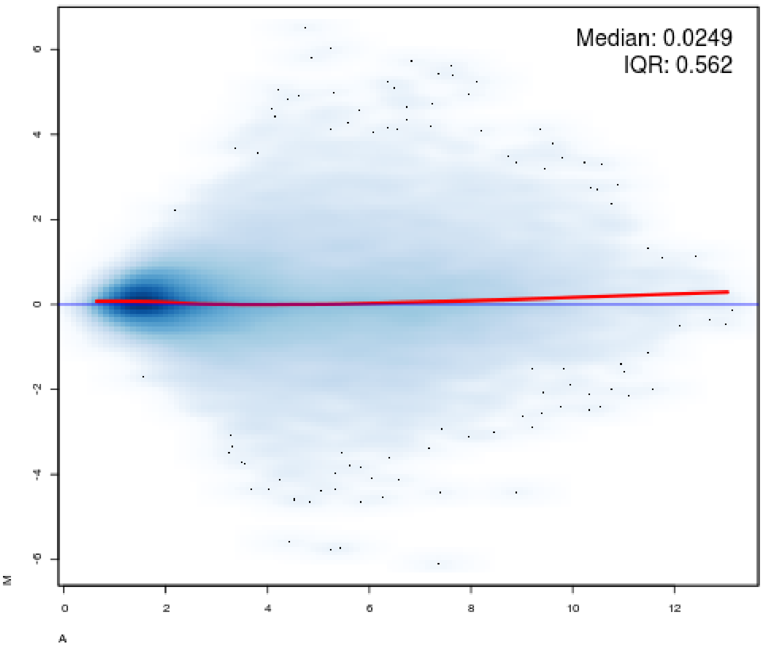

Array10 vs pseudo-median reference chip

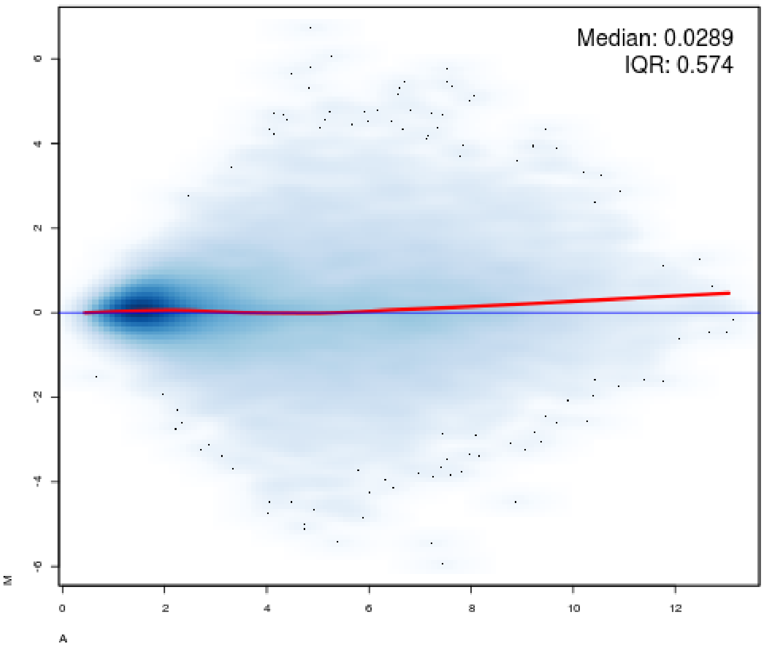

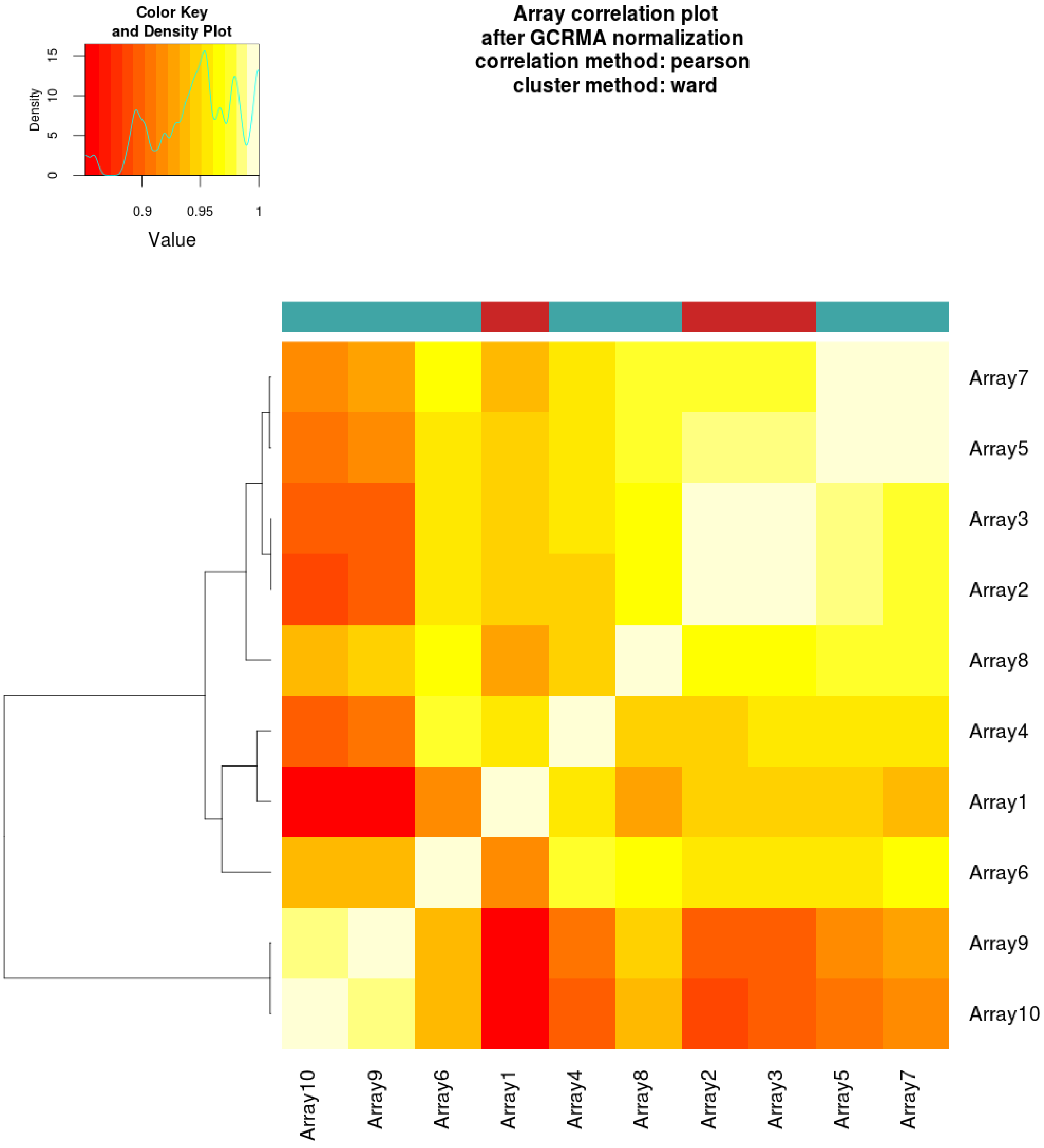

Color Key
and Density Plot
Density
0
5
10
15
0.9
0.95
1
Value
Array correlation plot
after GCRMA normalization
correlation method: pearson
cluster method: ward
Array7
Array5
Array3
Array2
Array8
Array4
Array1
Array6
Array9
Array10
Array10
Array9
Array6
Array1
Array4
Array8
Array2
Array3
Array5
Array7

PCA analysis after GCRMA normalization

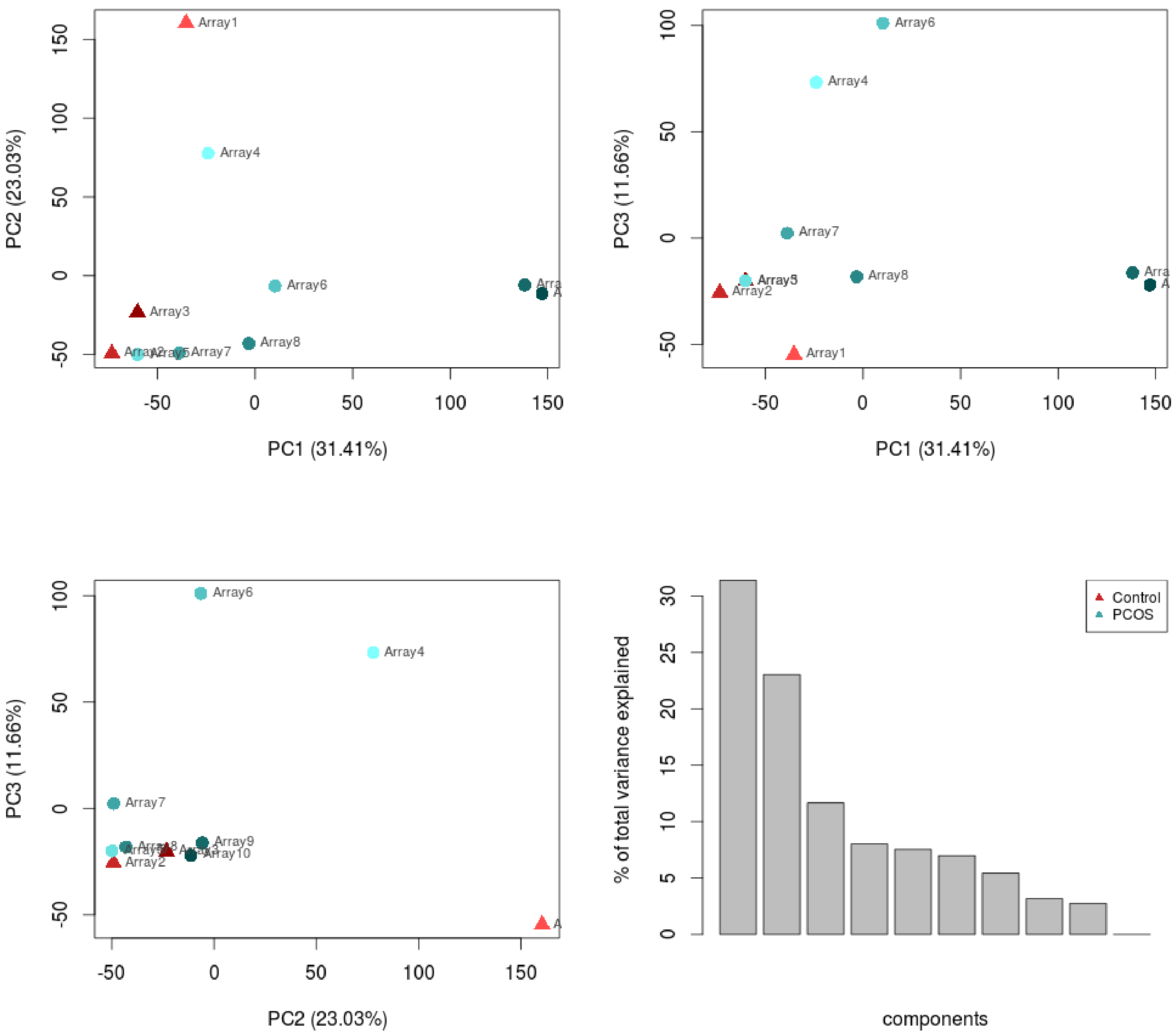

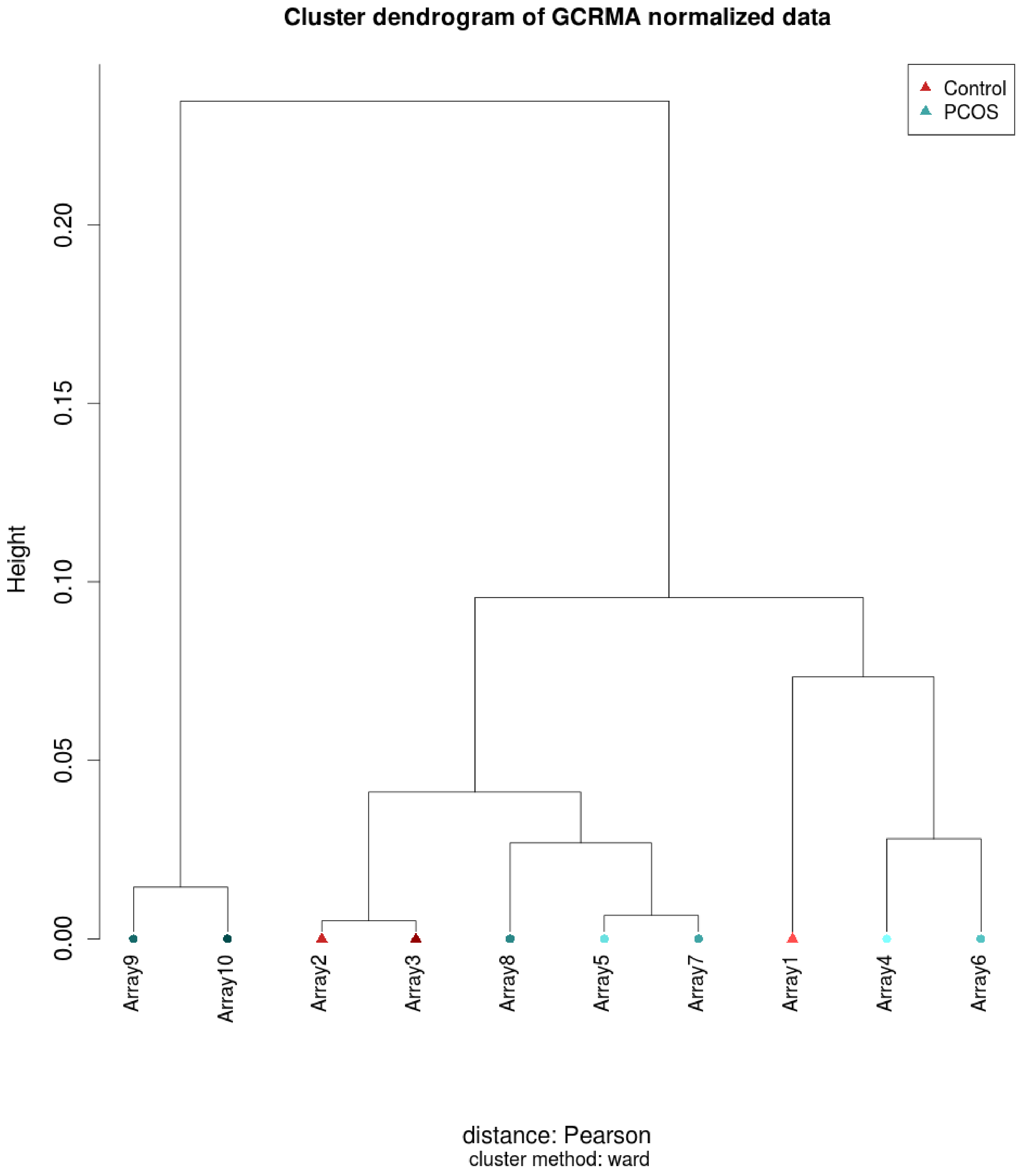
Cluster dendrogram of GCRMA normalized data
Control
PCOS
Height
0.00
0.05
0.10
0.15
0.20
Array9
Array10
Array2
Array3
Array8
Array5
Array7
Array1
Array4
Array6
distance: Pearson
cluster method: ward
