# Supplementary material for: Reactome from a WikiPathways Perspective
Source: PLoS Comput Biol. 2016 May 20;12(5):e1004941. doi: 10.1371/journal.pcbi.1004941 (PMC4874630; doi:10.1371/journal.pcbi.1004941)
Supplement: S2 Text — This guide describes the functionalities of the ComplexViz plugin and how it can be used in PathVisio. (PDF) [file pcbi.1004941.s007.pdf]

COMPLEXVIZ PLUGIN

User Guide

INSTALLATION

The plugin can be installed from the Plugin Manager in PathVisio (Fig. 1).

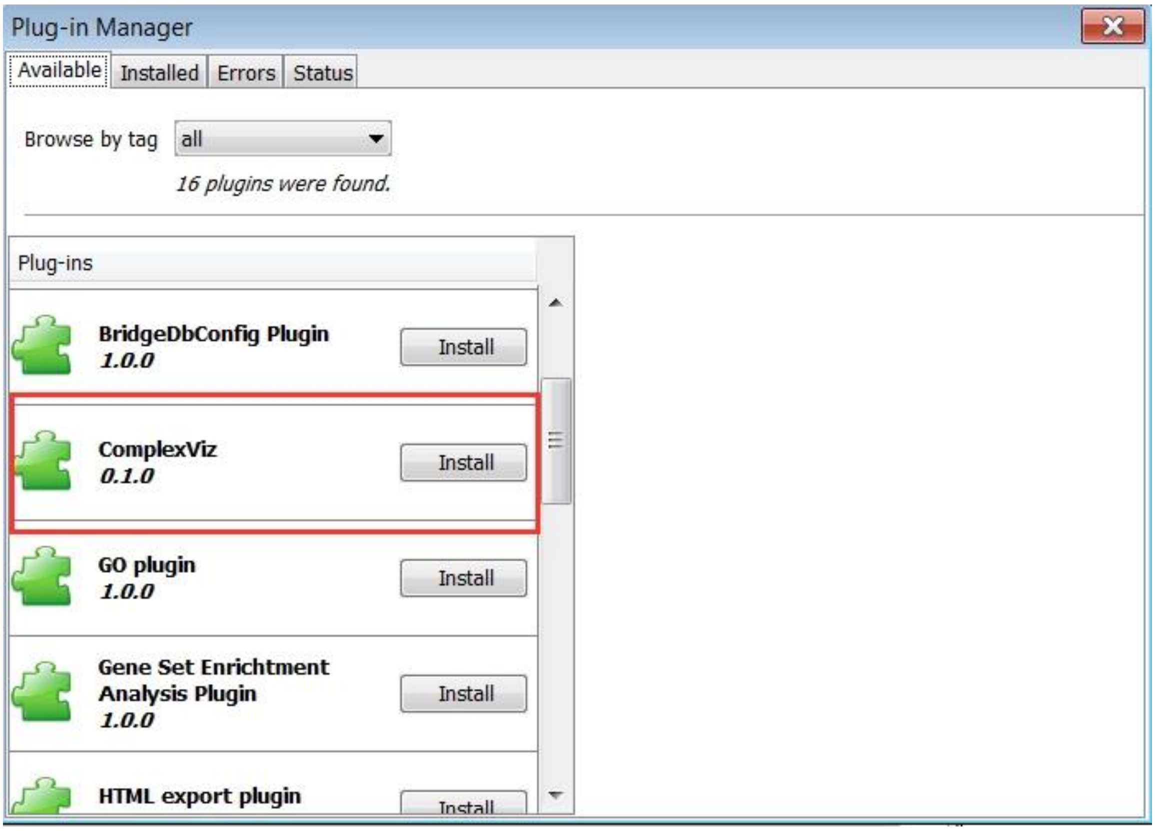

Fig. 1 Upon clicking "Plugins → Plugin Manager" in the PathVisio menu bar the plugin manager dialog box containing a list of plugins that can be installed is displayed. The ComplexViz plugin can be installed by clicking the button install beside the plugin.

The plugin adds a tab "Components" in the side panel and a menu item "ComplexViz Options" to the Data tab of the menu bar. The menu item is disabled and only gets enabled when expression data is loaded.

FUNCTIONALITY

Highlighting Complex Components

COMPONENTS TAB IN THE SIDE PANEL

When a pathway is opened in PathVisio, clicking on any complex node displays the complex components of the clicked complex node in the side tab "Components" (Fig. 2). When data is uploaded, the data is also visualized on these complex component nodes. Next to the complex components nodes is a list of buttons with the names of the complex components. Clicking these buttons displays the back page with the cross references of the clicked complex component on the bottom half of the side panel from all mapped databases, and the data if so loaded.

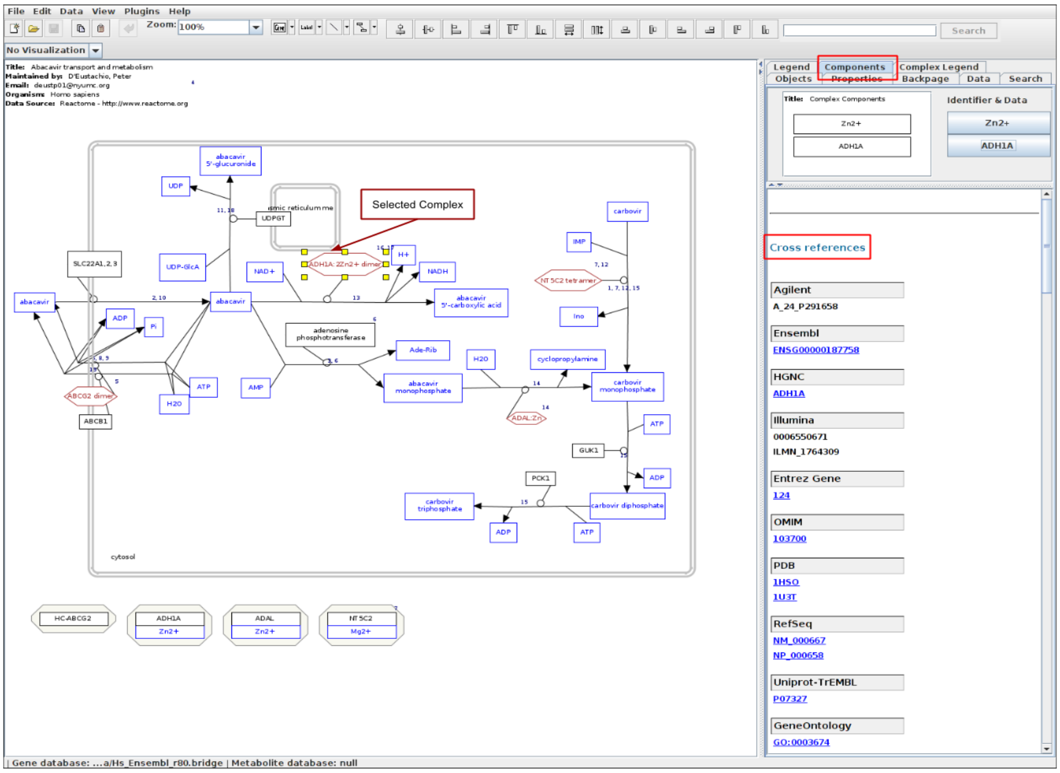

Fig. 2 The components of the complex selected in the pathway diagram are shown in the upper part of the Components side panel. On clicking on the buttons next to the complex components the cross references for that complex components is shown in the lower part of the same side panel.

COMPLEX NODE CONTEXT MENU

Complex component nodes for each complex node are arranged on the lower edge of the main pathway. Right clicking on a complex node in the main pathway displays a context menu which contains the option “Highlight complex / components”. Clicking this option highlights the complex components of that complex on the bottom of the pathway (Fig. 3).

Right clicking on a complex component node in the bottom of the pathway also displays the same context menu containing the option “Highlight complex / components”. Clicking this option highlights the complex node to which this component belongs in the main pathway.

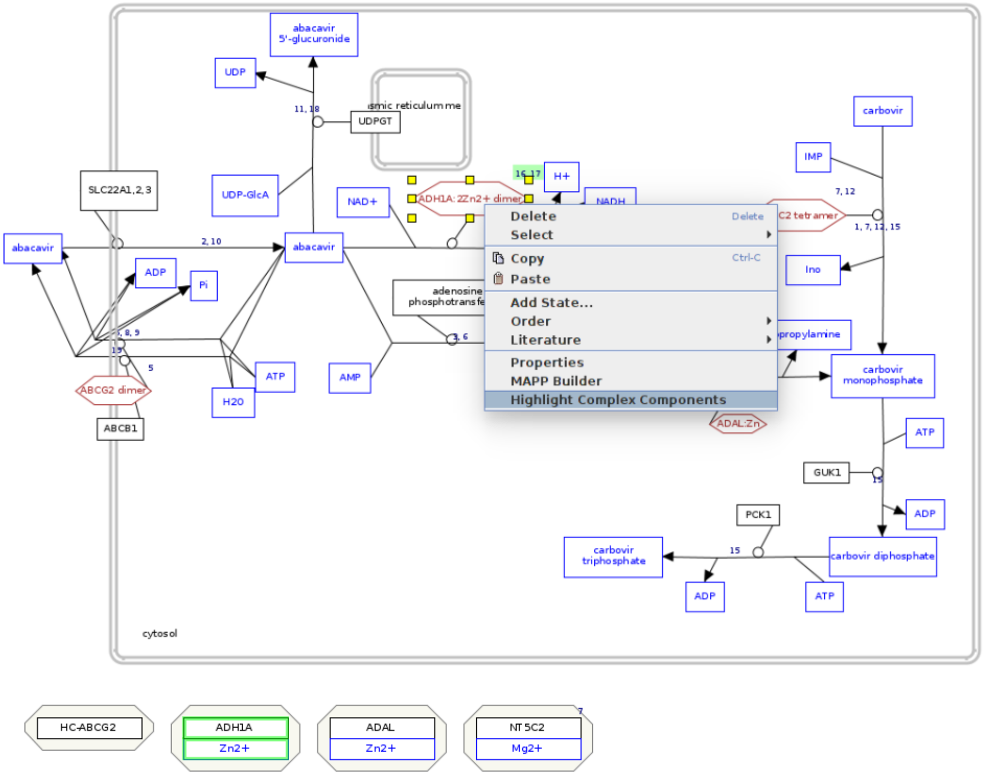

Fig. 3 Right clicking on a complex node in the pathway diagram and selecting the option "Highlight Complex/components" highlights the components of the complex at the bottom of the pathway diagram.

Visualizing data

LOAD DATA

For instructions on how to load data in PathVisio, consult the main PathVisio Users guide.

Link: http://www.pathvisio.org/documentation/using-experimental-data-in-pathvisio

After data is loaded, the "ComplexViz options" option becomes available in the **Data** tab of the menu bar. Clicking it opens a window with the visualization styles for complexes (Fig. 4).

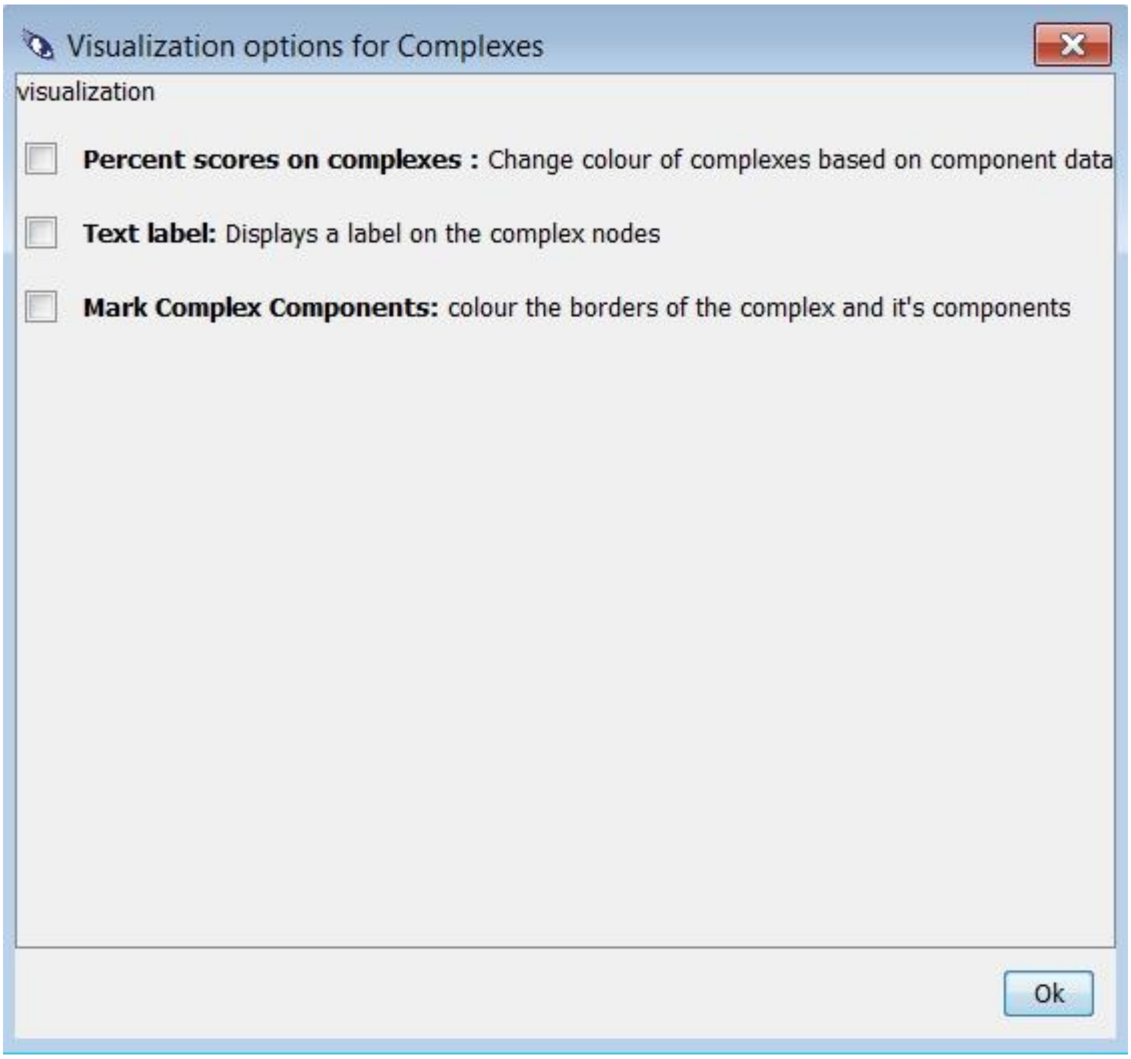

Fig. 4 Dialog box showing complex visualization styles available

PERCENT SCORE CALCULATION AND VISUALIZATION

Calculate the percentage of complex components fulfilling user defined criteria for each complex (Fig. 5). These criteria can for instance be p-value or fold change.

Table 1. Mock data for complex components

ID	logFC	P.value	Parent Complex
1234	1.5	0.01	Complex A
7653	0.5	0.06	Complex A
3452	0.9	0.03	Complex B
7546	1.7	0.02	Complex B

User defined criterion: logFC >=1.5 AND P.value <= 0.05

Percentage calculation

Complex A
x = 1
y = 3
Percentage
= (1/3)* 100
= 33%

Complex B
x = 1
y = 2
Percentage
= (1/2)* 100
= 50%

8465	1.0	0.08	Complex B

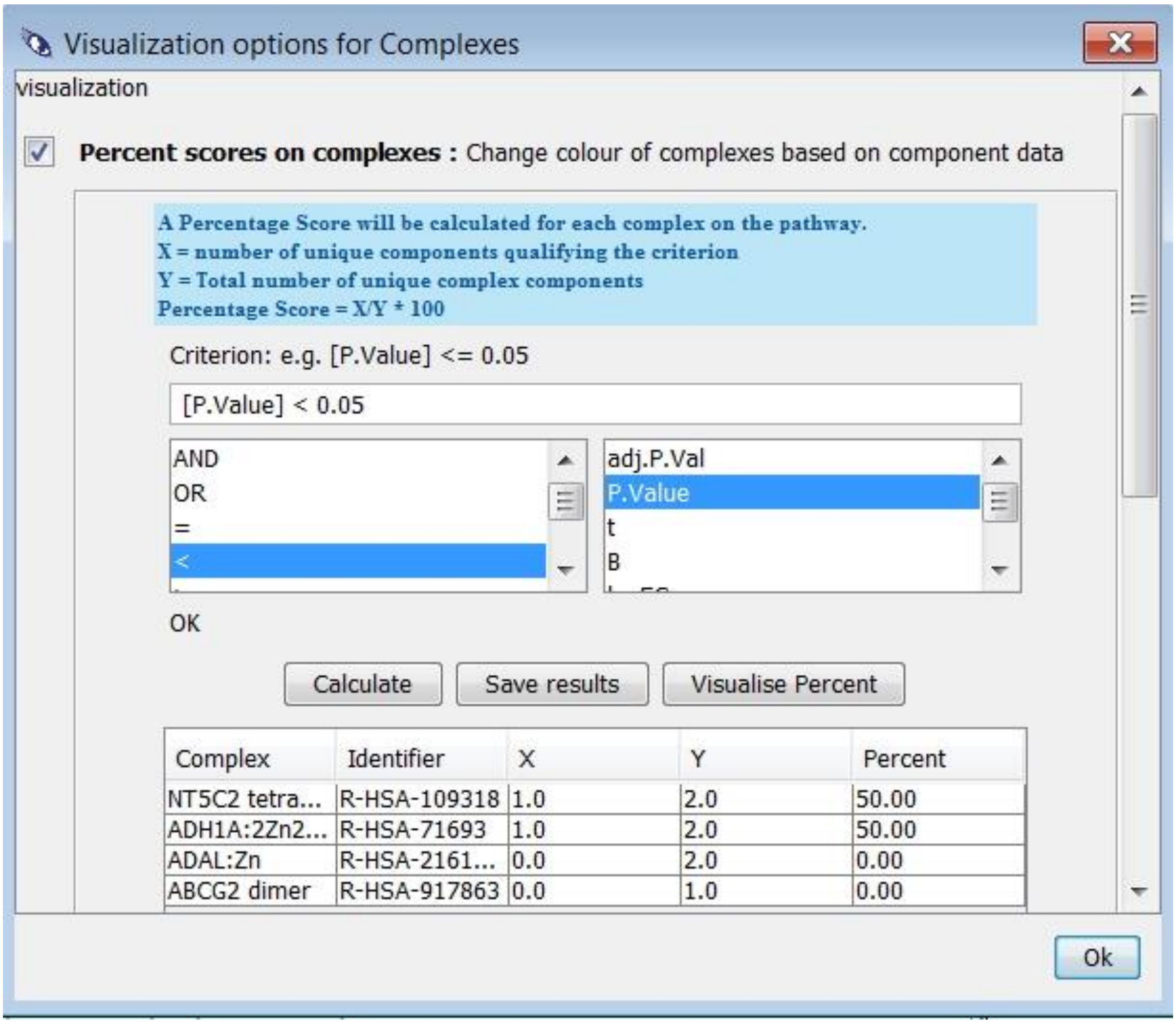

Fig. 5 Extension of dialog box for visualization options upon checking the "percent score visualization" option. The extension shows explanation for the score calculation, contains a text field for entering the criterion for calculating the scores for the complexes, shows the results as a table that can be saved, these results can then be visualized on the complex nodes in the pathway.

A table is displayed that shows the calculation for each complex. The data can be saved as text file. The percentage calculated for each complex can be visualized on the complex node using color rule or color gradient (Fig. 6).

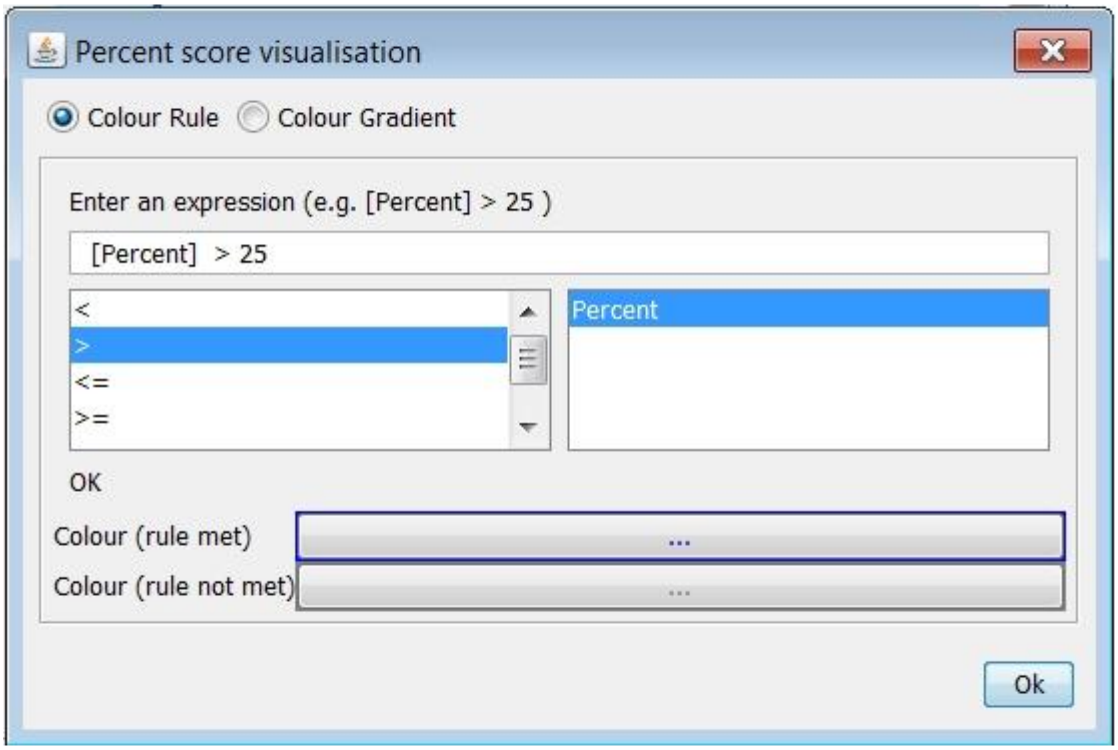

Fig. 6 Dialog box for selecting visualization styles for visualizing percent scores on complexes in the pathway. A color rule or gradient can be selected.

MARK COMPLEX COMPONENTS

Choosing this option allows the user to choose a color for the border of each complex node (Fig. 7). The same color border appears around the corresponding complex components along the bottom edge of the pathway.

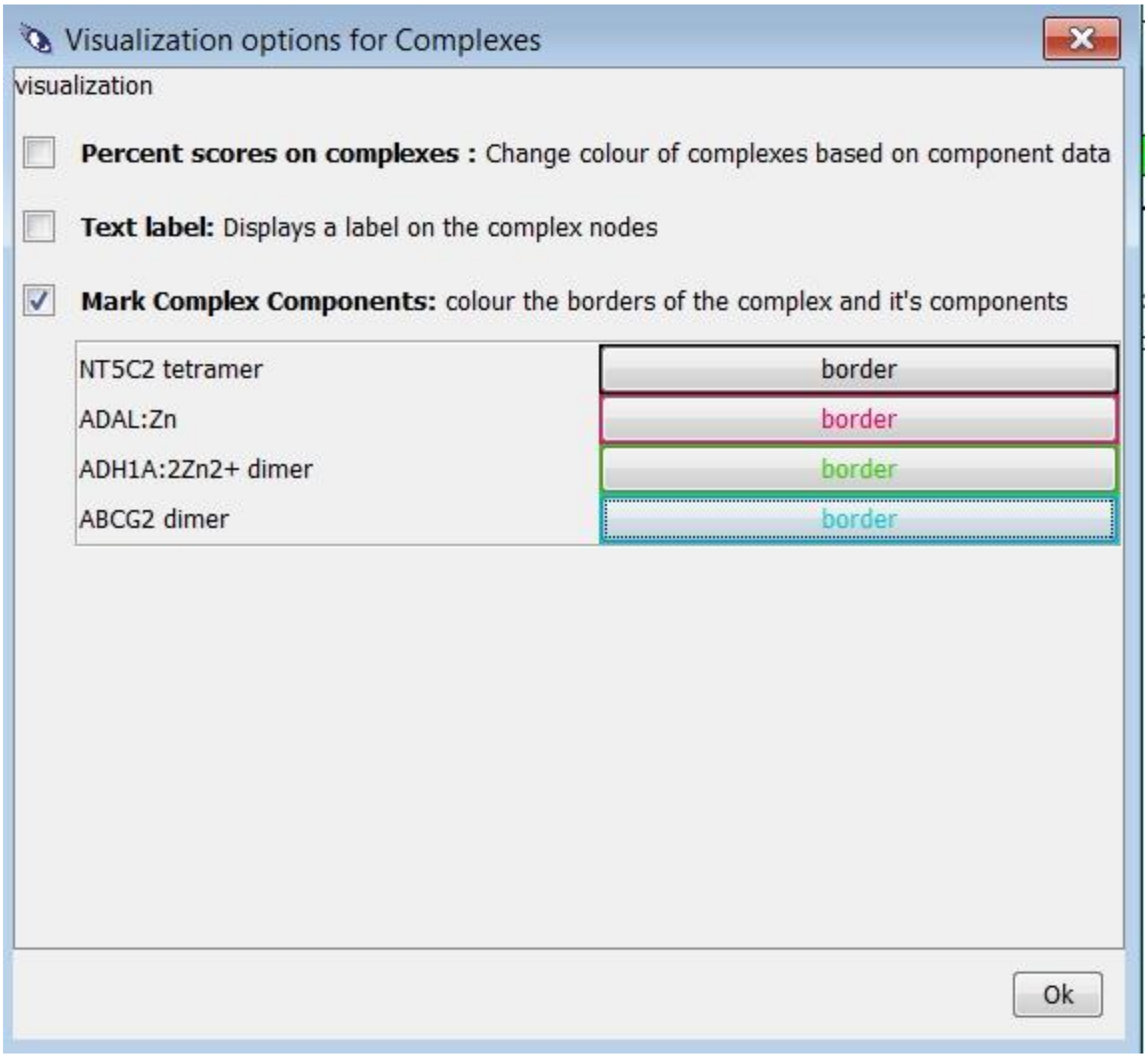

Fig. 7 Dialog box showing additional options for selecting border color for each complex on the pathway on selecting the "Mark complex components".

TEXT LABEL

Ticking this option displays text labels on complex nodes (Fig. 8). The text label can be the name or the ID of the complex and the font is fully customizable.

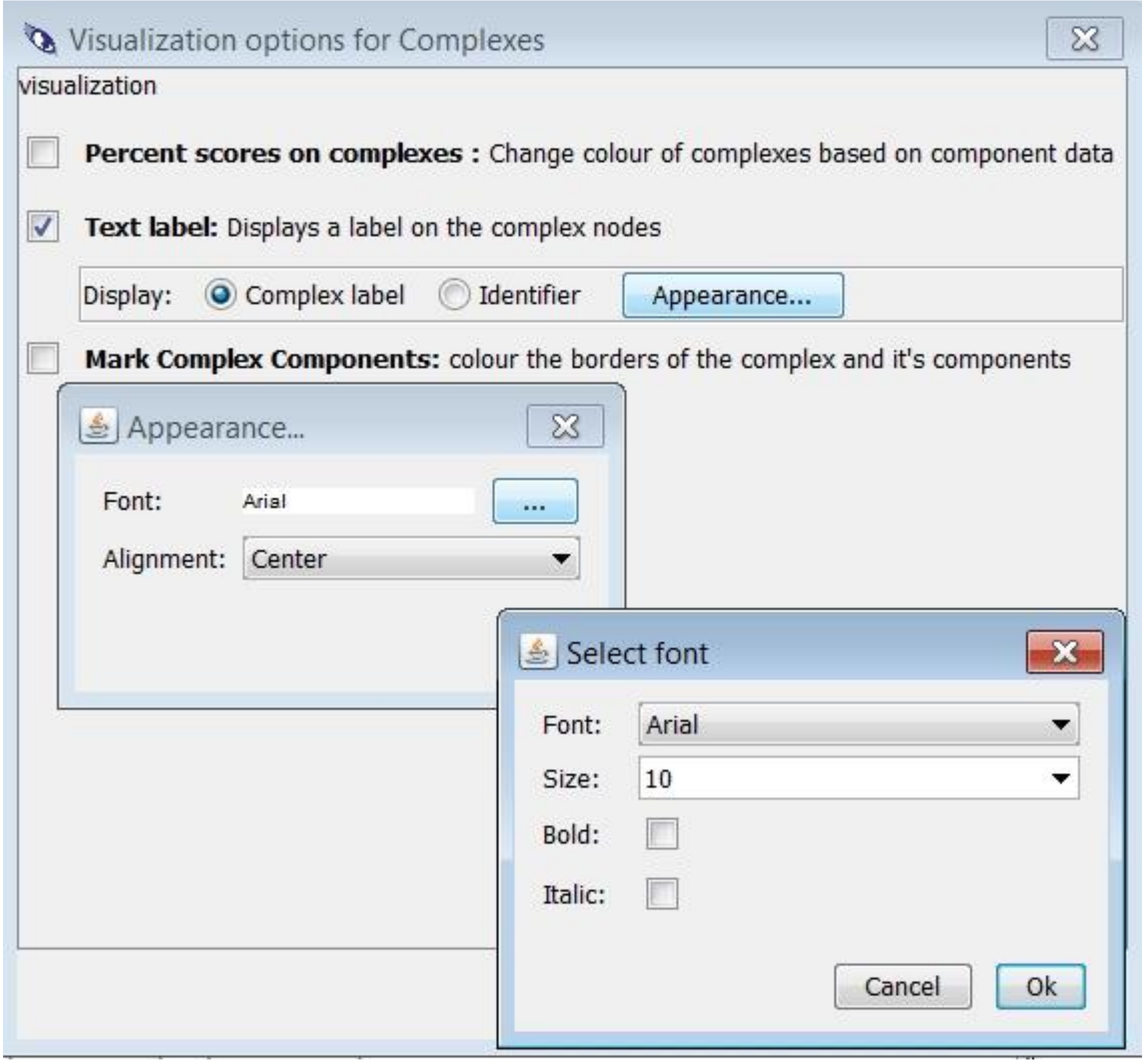

Fig. 8 Dialog box showing additional options for selecting the style, size and alignment of the text label for each complex on the pathway on selecting the "Text Label".

EXAMPLE USAGE

An example dataset was visualized on the Abacavir transport and metabolism pathway from the reactome_approved collection of WikiPathways to demonstrate the use of the ComplexViz plugin (Fig. 9).

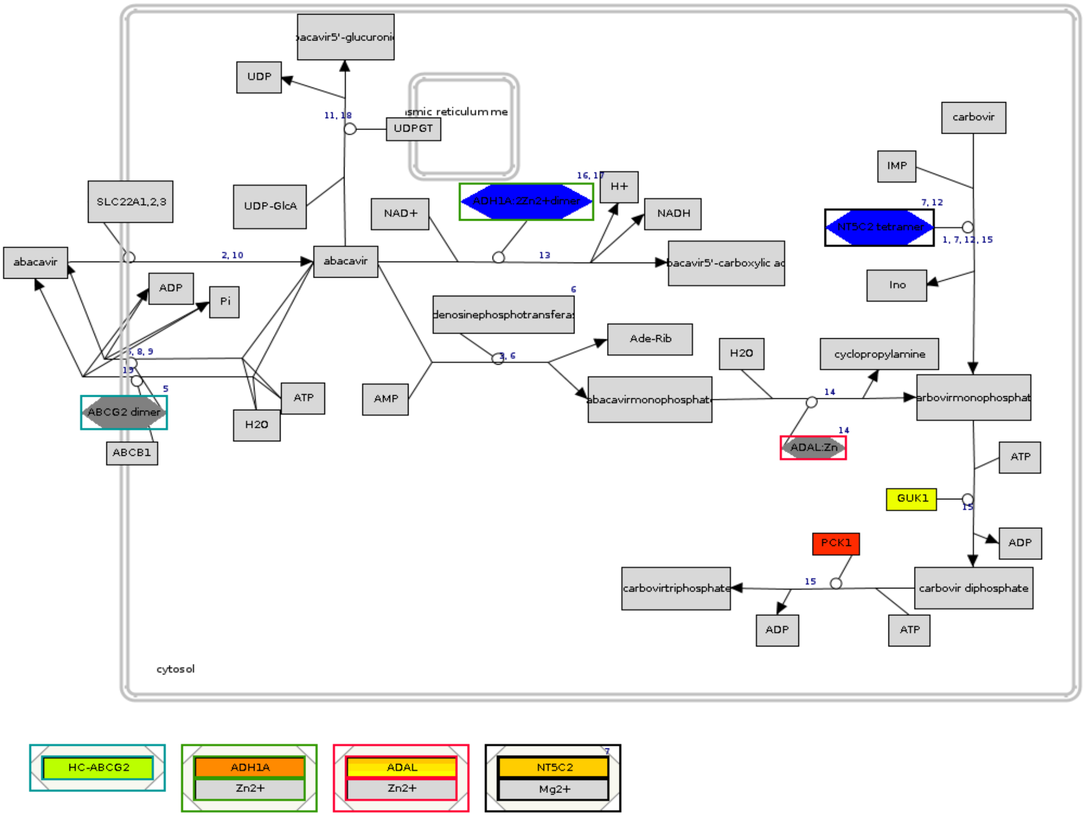

Fig. 9 An example dataset is visualized on the pathway. Complexes and their components highlighted with the same border color and percent scores visualized on complex nodes. Complexes with a percent score higher than 25 are colored in blue, the rest dark gray.
